# Unveiling patterns in spatial transcriptomics data: a novel approach utilizing graph attention autoencoder and multiscale deep subspace clustering network

**DOI:** 10.1093/gigascience/giae103

**Published:** 2025-01-13

**Authors:** Liqian Zhou, Xinhuai Peng, Min Chen, Xianzhi He, Geng Tian, Jialiang Yang, Lihong Peng

**Affiliations:** School of Computer Science, Hunan University of Technology, Zhuzhou 412007, Hunan, China; School of Computer Science, Hunan University of Technology, Zhuzhou 412007, Hunan, China; School of Computer Science, Hunan Institute of Technology, Hengyang 421002, Hunan, China; School of Computer Science, Hunan University of Technology, Zhuzhou 412007, Hunan, China; Geneis (Beijing) Co. Ltd., Beijing 100102, China; Geneis (Beijing) Co. Ltd., Beijing 100102, China; School of Computer Science, Hunan University of Technology, Zhuzhou 412007, Hunan, China; College of Life Science and Chemistry, Hunan University of Technology, Zhuzhou 412007, Hunan, China

**Keywords:** spatial transcriptomics, graph attention autoencoder, deep subspace clustering, multiscale self-expression, self-supervised learning, latent embedding feature learning, cell type–aware spatial neighbor network, differential expression analysis, trajectory inference

## Abstract

**Background:**

The accurate deciphering of spatial domains, along with the identification of differentially expressed genes and the inference of cellular trajectory based on spatial transcriptomic (ST) data, holds significant potential for enhancing our understanding of tissue organization and biological functions. However, most of spatial clustering methods can neither decipher complex structures in ST data nor entirely employ features embedded in different layers.

**Results:**

This article introduces STMSGAL, a novel framework for analyzing ST data by incorporating graph attention autoencoder and multiscale deep subspace clustering. First, STMSGAL constructs ctaSNN, a cell type–aware shared nearest neighbor graph, using Louvian clustering exclusively based on gene expression profiles. Subsequently, it integrates expression profiles and ctaSNN to generate spot latent representations using a graph attention autoencoder and multiscale deep subspace clustering. Lastly, STMSGAL implements spatial clustering, differential expression analysis, and trajectory inference, providing comprehensive capabilities for thorough data exploration and interpretation. STMSGAL was evaluated against 7 methods, including SCANPY, SEDR, CCST, DeepST, GraphST, STAGATE, and SiGra, using four 10x Genomics Visium datasets, 1 mouse visual cortex STARmap dataset, and 2 Stereo-seq mouse embryo datasets. The comparison showcased STMSGAL’s remarkable performance across Davies–Bouldin, Calinski–Harabasz, S_Dbw, and ARI values. STMSGAL significantly enhanced the identification of layer structures across ST data with different spatial resolutions and accurately delineated spatial domains in 2 breast cancer tissues, adult mouse brain (FFPE), and mouse embryos.

**Conclusions:**

STMSGAL can serve as an essential tool for bridging the analysis of cellular spatial organization and disease pathology, offering valuable insights for researchers in the field.

Key Points

- A graph attention autoencoder is fully utilized to effectively integrate spatial locations and gene expression information by collectively incorporating information between neighboring spots.
- A multiscale self-expression module is explored to learn the associations between node representations in all encoder layers and further obtain a more distinct self-expression coefficient matrix for mapping these features into a more precise subspace.
- A self-supervised learning method is designed to help spot latent feature learning by utilizing the clustering label as a supervisor.

## Background

The tissues in the human body comprise various cell types, where each cell type implements a particular function [1]. The activation of a cell is mainly affected by its surrounding environment [2–5]. Exploring relative positions of these cells contributes to analyzing cell–cell communication [6–9] and their spatial organization and disease pathology [10–13]. The rapid advance of single-cell RNA sequencing (scRNA-seq) technologies enables us to investigate the gene expression patterns of various cells within a tissue/organ [14–22]. However, scRNA-seq technologies fail to provide spatial location information [23]. In contrast, spatial transcriptomics (ST) technologies provide a large number of gene expression data and cellular location information for a tissue and have witnessed tremendous development in the past several years [24–26]. Based on data generation methods, ST technologies mainly contain image-based methods and next-generation sequencing (NGS)–based methods [27].

Image-based methods use *in situ* sequencing or *in situ* hybridization to retain spatial locations of cells and further obtain RNA transcripts based on images from the stained tissues. MERFISH [28] can detect gene expression information of about 40,000 human cells in a single 18-hour measurement. STARmap [29] can capture more than 1,000 genes in the mouse cortex through an error-robust sequencing-by-ligation approach. seqFISH+ [30] combined sequential hybridization and standard confocal microscope–based imaging technique to obtain super-resolution imaging and multiplexing data for 10,000 genes.

NGS-based methods depend on the number of spatial barcodes before library preparation [31]. Slide-seq [32, 33] obtained randomly barcoded positions through *in situ* indexing and captured mRNAs through depositing onto a slide. High-definition ST (HDST) [34] replaced the glass slides using beads deposited in wells. The DBiT-seq [35] technique utilized polyT barcodes in the tissue section based on microfluidics. Stereo-seq [36] obtained nanoscale resolution through randomly barcoded DNA nanoballs. 10x Genomics Visium [37] demonstrated increased resolution with a diameter of 55 µm and a 100-µm center–center, as well as improved sensitivity in more than 10,000 transcripts per spot. It detected more unique molecules for each spot compared with Slide-seq and HDST.

One main challenge in ST data analysis is to capture spatial domains with similar expression patterns. For example, the laminar organization in human cerebral cortex has a close relationship with its biological functions. In this tissue, cells within different cortical layers have different expressions, morphology, and physiology [38]. One efficient way to identify spatial domains is to cluster ST data. These clustering methods mainly fall into 2 categories. The first category adopts conventional clustering methods, for example, *K*-means clustering [39] and Louvain algorithms [40]. These algorithms are susceptible to the small size of spots and sparsity data, and the detected clusters may be discontinuous in sections. The other category uses cell-type labels obtained from scRNA-seq data to deconvolute spots [41, 42], but these types of methods cannot analyze ST data from the perspective of cellular or subcellular resolution.

It is crucial to learn a discriminative representation for each spot by combining gene expression and spatial contexts when clustering ST data. Recently, several clustering algorithms have been developed to identify spatial domains. For example, BayesSpace [43] assumed that spots belonging to the same cell type may be closer to each other and built a Markov random field model with Bayesian approach. stLearn [44] first proposed a spatial morphological gene expression normalization algorithm to normalize ST data and then employed a standard Louvain clustering approach to partition broad clusters into several subclusters. SEDR [45] exploited a deep autoencoder network to learn gene representations and adopted a variational graph autoencoder to embed spatial information. CCST [46] explored a graph convolutional network to transfer gene expression information as cellular embedding vectors and trained a neural network to encode cell embedding features for clustering. STAGATE [47] developed a adaptive graph attention autoencoder (GATE) [48] to accurately identify spatial domains by integrating gene expression information and spatial neighbor network. DeepST [49] incorporated gene expression, spatial context, and histology to model spatially embedded representation and further capture spatial domains. GraphST [50] integrated graph self-supervised contrastive learning and a graph neural network [51, 52] for spatial clustering, multisample integration, and cell-type deconvolution. ConGI [53] adopted gene expression with histopathological images to accurately capture spatial domains based on contrastive learning. STGIC is a graph- and image-based spatial clustering method. It can generate pseudo-labels for spatial clustering but does not depend on any trainable parameters. SPACEL [54] deconvoluted cell-type composition based on a multiple-layer perceptron, identified spatial domains via a graph convolutional network and adversarial learning, and constructed a 3-dimensional architecture for each tissue. PRECAST [55] integrated a few ST datasets that have complex batch effects and biological effects. SRTsim [56] is spatially resolved transcriptomics-specific simulator for spatial clustering and expression pattern analysis. Tang et al. [57] developed an image-augmented graph transformer for spatial elucidation. The methods mentioned above have significantly promoted the studies of tissue physiology from cell centroid to structure centroid and are state-of-the-art spatial clustering methods. In prticular, Yuan et al. [58] considered that current computation-based ST clustering is a lack of a comprehensive benchmark and systematically benchmarked a collection of 13 spatial clustering methods on 7 ST datasets (34 ST data). Their work has provided guidance for future progress in the ST data analysis field.

Although the aforementioned clustering methods obtained impressive performance, their learned latent node representation failed to achieve the most useful information because they did not use current clustering labels. In addition, some methods, including SEDR and CCST, only used the representation in the final hidden layer of an encoder for clustering ST data, which failed to consider helpful features in the other layers. Although graph attention autoencoder-based methods [59, 60] have elucidated better performance in integrating node attributes and graph structure information, they could not decipher the complex structures in ST data or did not entirely employ features embedded in different layers. Moreover, some models did not utilize a clustering-oriented loss function, while others did not fully use the clustering labels for node representation learning. The problems produced the suboptimal clustering results. Here, we introduce STMSGAL, an ST analysis framework by combining a graph attention autoencoder and multiscale deep subspace clustering network.

## Materials and Methods

### Overview of STMSGAL

**Figure 1:**
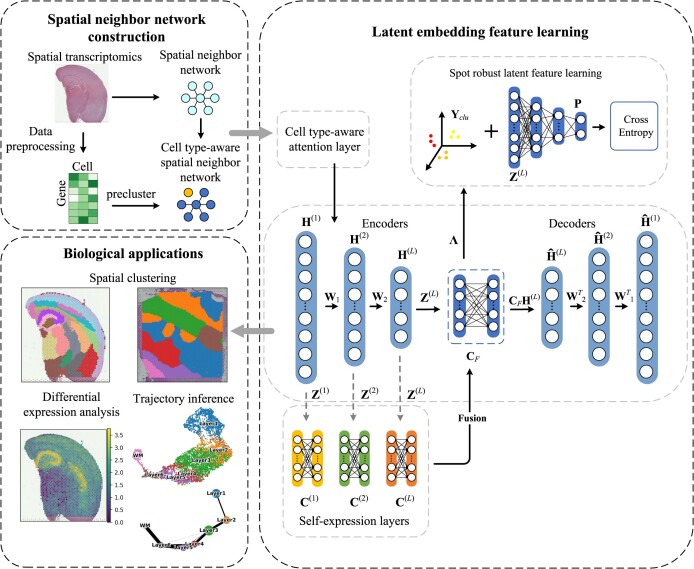
Pipeline for clustering ST data based on GATE and deep subspace clustering network. (i) Spatial neighbor network construction. (ii) Latent embedding feature learning. (iii) Biological applications.

As shown in Fig. 1, STMSGAL is composed of 3 main steps: (i) Spatial neighbor network construction. STMSGAL constructs a spatial neighbor network (SNN) based on spatial contexts and obtains a cell type–aware SNN called ctaSNN through Louvain clustering exclusively based on gene expression data. (ii) Latent embedding feature learning. This mainly comprises spot embedding feature matrix construction, subspace clustering combining multiscale self-expression coefficient learning and affinity matrix construction, and spot robust latent feature learning based on self-supervised learning. (iii) Biological applications. ST data are clustered, and differential expression analysis and trajectory inference are implemented. Similar to STAGATE [47], STMSGAL still constructs a ctaSNN and embedding feature matrix using GATE. However, different from STAGATE, STMSGAL adopts the multiscale deep subspace clustering algorithm to obtain cluster labels based on multiscale information from each encoder layer for spots and then adopts a self-supervised module to learn robust latent features of spots with clustering information.

### Datasets

Four available 10x Genomics Visium datasets, 1 mouse visual cortex STARmap dataset, and 2 Stereo-seq datasets are used to evaluate the STMSGAL performance. The former four 10x Genomics datasets are from Adult Mouse Brain (FFPE) [61], Human Breast Cancer (Block, A Section 1) [62], Human Breast Cancer (Ductal Carcinoma In Situ [DCIS]) [63], and Human Dorsolateral Prefrontal Cortex (DLPFC) tissues [64]. The former 2 datasets have no clustering labels, and the latter 2 datasets are known to be labeled. The Adult Mouse Brain (FFPE) dataset contains 2,264 spots and 19,465 genes. The Human Breast Cancer (DCIS) dataset includes 3,798 spots and 36,601 genes. The Human Breast Cancer (Block A, Section 1) dataset detects 2,518 spots and 19,743 genes. The DLPFC dataset contains 12 tissue slices. It captures 33,538 genes with different spot numbers ranging from 3,460 to 4,789 in each slice. Each slice contains 5 to 7 regions by manual annotation [38]. The mouse visual cortex STARmap dataset provides the expression information of 1,020 genes from 1,207 cells [29]. The Stereo-seq dataset [65] from mouse embryos at E9.5 is obtained based on high-resolution full-transcriptome coverage technologies (i.e., Stereo-seq technology). The number of spots and one of genes are 5,913 and 25,568 (E9.5_E1S1), as well as 4,356 and 24,107 (E9.5_E2S2), respectively.

### Spatial neighbor network construction

#### Data preprocessing

To preprocess ST data, first, spots outside main tissue regions are removed. Next, raw gene expressions are log-transformed and normalized based on library size through the SCANPY package [66]. Finally, multiple highly variable genes are selected as inputs.

#### Cell type–aware SNN construction

To integrate the similarity between a neighboring spot and a given spot, similar to STAGATE [47], STMSGAL constructs an undirected neighbor network based on a predefined radius *r* and spatial contexts. Let $\boldsymbol {A}$ denote an adjacency matrix of the constructed SNN, and $\boldsymbol {A}_{i j}$ = 1 when the Euclidean distance between 2 spots *i* and *j* is less than *r*. For 10x Genomics Visium data, an SNN where each spot contains 6 nearest neighbors is built. Next, self-loops are added to each spot. Finally, the SNN is pruned based on preclustering and a ctaSNN is constructed. In particular, the preclustering of spots is conducted by Louvain clustering [40] exclusively based on gene expression profiles. The edges where 2 spots linking them belong to different clusters are pruned.

### Latent embedding feature learning

Wang et al. [67] presented a multiscale graph attention subspace clustering model and obtained superior performance on 3 graph datasets and 2 real-world datasets. The clustering model fully explored the associations between node representations in all encoder layers and obtained a more accurate self-expression coefficient matrix. To more accurately cluster spots, in this section, we utilize the multiscale graph attention subspace clustering model [67] to learn latent embedding features of spots. First, a spot embedding feature matrix in each encoder layer is constructed via GATE. Second, spot cluster labels are obtained through subspace clustering. Finally, spot robust latent features are learned by self-supervised learning.

#### Embedding feature matrix construction

Similar to STAGATE [47], we use GATE to construct an embedding feature matrix. For spot *i*, an encoder with *L* layers takes its normalized gene expressions $\boldsymbol {x}_i$ as inputs to generate its embedding features by collectively incorporating information from its neighbors. Taking gene expressions as initial spot embeddings, that is, $\boldsymbol {h}_i^{(0)} = \boldsymbol {x}_i$, $\forall i \in \left\lbrace 1,2,\cdots , N \right\rbrace $, the embedding of *i* in the *k*th ($k\in \left\lbrace 1,2, \cdots , L-1 \right\rbrace$) encoder layer is denoted by Eq. (1):


(1) \begin{eqnarray*}
\boldsymbol {h}_i^{(k)}=\sigma \left(\sum _{j \in S_i} \boldsymbol {a t t}_{i j}^{(k)}\left(\boldsymbol {W}_k \boldsymbol {h}_j^{(k-1)}\right)\right)
\end{eqnarray*}

where $\boldsymbol {W}_k$, $\sigma$, $S_i$, and $\boldsymbol {a t t}_{ij}^{(k)}$ denote the trainable weight matrix, nonlinear activation function, a spot set that includes neighbors of *i* in SNN and $i$ itself, and weight of the edge between spot $i$ and spot $j$ in the $k$th graph attention layer, respectively. The output $\boldsymbol {z}_i^{(k)} = \boldsymbol {h}_i^{(k)}$ of the encoder is taken as the final spot embedding in the encoder part. The $L$th layer in the encoder does not use the attention layer by Eq. (2):


(2) \begin{eqnarray*}
\boldsymbol {h}_i^{(L)}=\sigma \left(\boldsymbol {W}_L \boldsymbol {h}_i^{(L-1)}\right)
\end{eqnarray*}

In the decoder part, a decoder transforms the learned latent embedding back into a normalized expression profile to reconstruct the spot features. Suppose that $\widehat{\boldsymbol {h}}_i^{(L)} = \boldsymbol {C} \boldsymbol {z}_i^{(L)}$, where $\boldsymbol {C}$ denotes a self-expression matrix, and $\boldsymbol {z}_i^{(L)}$ denotes the embedding of $i$ in the $L$th encoder layer. Next, $\boldsymbol {C} \boldsymbol {z}_i^{(L)}$ is fed into the decoder to reconstruct the spot embeddings. In the $k$th decoder layer, the embedding features of spot $i$ are constructed by Eq. (3):


(3) \begin{eqnarray*}
\widehat{\boldsymbol {h}}_i^{(k-1)}=\sigma \left(\sum _{j \in S_i} \widehat{\boldsymbol {a t t}}_{i j}^{(k-1)}\left(\widehat{\boldsymbol {W}}_k \widehat{\boldsymbol {h}}_j^{(k)}\right)\right)
\end{eqnarray*}

The *L* layer in the decoder is denoted by Eq. (4):


(4) \begin{eqnarray*}
\widehat{\boldsymbol {h}}_i^{(0)}=\sigma \left(\widehat{\boldsymbol {W}}_1 \widehat{\boldsymbol {h}}_i^{(1)}\right)
\end{eqnarray*}

Its output is the reconstructed normalized expressions. In addition, we set $\widehat{\boldsymbol {W}}_{k} = \boldsymbol {W}_k{^T}$ and $\widehat{\boldsymbol {att}}^{(k)} = \boldsymbol {att}^{(k)}$ to avoid overfitting.

The attention mechanism is a 1-layer feed-forward neural network that is parametrized by a weight vector. A self-attention mechanism [68] is used to compute the similarity between neighboring spots in an adaptive way. In the *k*th decoder layer, the edge weight between spot $i$ and its neighbor spot $j$ is computed by Eq. (5):


(5) \begin{eqnarray*}
e_{i j}^{(k)}=\operatorname{Sigmoid}(\boldsymbol {v}_s^{(k)^T}(\boldsymbol {W}_k \boldsymbol {h}_i^{(k-1)})+\boldsymbol {v}_r^{(k)^T}(\boldsymbol {W}_k \boldsymbol {h}_j^{(k-1)}))
\end{eqnarray*}

where $\boldsymbol {v}_s^{(k)}$ and $\boldsymbol {v}_r^{(k)}$ are 2 trainable weight vectors. Next, the similarity weights between spots are normalized by a softmax function by Eq. (6):


(6) \begin{eqnarray*}
att_{i j}^{(k)}=\frac{\exp \left(e_{i j}^{(k)}\right)}{\sum _{j \in {S}_i} \exp \left(e_{i j}^{(k)}\right)}
\end{eqnarray*}

The obtained weights are applied to further update the latent embedding of spots in the encoder and decoder.

In addition, STMSGAL adopts a self-attention mechanism and constructs a ctaSNN. Let $\boldsymbol {att}_{i j}^{spatial}$ and $\boldsymbol {att}_{i j}^{aware}$ denote the learned spot similarity using SNN and ctaSNN, respectively, and the final spatial similarity is computed by combining the above 2 similarities by Eq. (7):


(7) \begin{eqnarray*}
\boldsymbol {att}_{i j} = \left(1- \alpha \right)\boldsymbol {att}_{i j}^{\text{spatial}} + \alpha \boldsymbol {att}_{i j}^{\text{aware}}
\end{eqnarray*}

where $\alpha$ is a hyperparameter used to weigh the importance of SNN and ctaSNN.

The reconstructed loss is minimized based on the residual sum of squares by Eq. (8):


(8) \begin{eqnarray*}
\mathcal {L}_{att }=\min \frac{1}{2} \sum _{i=1}^n\left\Vert \boldsymbol {x}_i-\hat{\boldsymbol {h}}_i^{(0)}\right\Vert _F^2
\end{eqnarray*}

In particular, weight decay equally imposes a penalty to the $L_2$ norm; thus, the regularized loss is minimized. The total loss is represented as Eq. (9):


(9) \begin{eqnarray*}
\begin{split} \mathcal {L}_{1}=\mathcal {L}_{att}+\frac{1 }{2} \cdot \sum _{k=1}^{L-1}\left\Vert \boldsymbol {W}_k\right\Vert ^{2}_{F} \end{split}
\end{eqnarray*}

#### Multiscale deep subspace clustering

Different from STAGATE [47], in this section, we adopt the multiscale deep subspace clustering algorithm to obtain cluster labels based on multiscale information from each encoder layer for spots. The self-expression property of data greatly influences the performance of subspace clustering. In a union subspace, each datum can be represented as a linear combination of the other data. Thus, we use a multiscale self-expressive module to obtain the final self-expression coefficient matrix based on the spot embedding feature matrix: $\boldsymbol {H}^{(k)} = \left\lbrace \boldsymbol {h}_1^{(k)}, \boldsymbol {h}_2^{(k)}, \cdots , \boldsymbol {h}_n^{(k)} \right\rbrace $.

In deep subspace clustering network [69], a self-expression layer is a full connection layer without bias and activation. Its objection function is represented by Eq. (10):


(10) \begin{eqnarray*}
\min _{\boldsymbol {C}}\Vert \boldsymbol {C}\Vert _p+\frac{1}{2}\Vert \boldsymbol {Z}-\boldsymbol {C Z}\Vert _F^2 \quad \textit { s.t. } \quad (\operatorname{diag}(\boldsymbol {C})=0)
\end{eqnarray*}

where $\boldsymbol {C}$ indicates a self-expression coefficient matrix used to build an affinity matrix $\boldsymbol {\mathit {\Lambda }}$ for the following spectral clustering, $\boldsymbol {Z}$ indicates the output feature matrix in the encoder, and $\Vert \cdot \Vert _{p}$ indicates an arbitrary regularization norm.

Although deep subspace clustering obtains better clustering performance, it fails to consider the multiscale features existing in the other encoder layers. Here, we integrate the multiscale features into the original self-expression module. Given the input normalized gene expressions in the *i*th encoder layer $\boldsymbol {Z}^{(k)} \left(k = 1,2, \cdots , L \right)$, the self-expression coefficient matrix $\boldsymbol {C}^{(k)}$ in the *k*th encoder layer can be computed by Eq. (11):


(11) \begin{eqnarray*}
\min _{\boldsymbol {C}^{(k)}} \frac{1}{2}\left\Vert \boldsymbol {Z}^{(k)}-\boldsymbol {C}^{(k)} \boldsymbol {Z}^{(k)}\right\Vert _{F}^{2}
\end{eqnarray*}

Next, the multiscale self-expression matrix $\boldsymbol {C}^{(k)}$ in different layers is fused based on an adaptive approach by Eq. (12):


(12) \begin{eqnarray*}
\boldsymbol {C}_{F}=\frac{\sum _{k=1}^{L} \tau _{k} \cdot \boldsymbol {C}^{(k)}}{\sum _{k=1}^{L} \tau _{k}}
\end{eqnarray*}

where $\tau _{k}$ denotes a trainable variable used to balance the importance of each self-expression matrix.

Based on the obtained final self-expression matrix $\boldsymbol {C}_F$, a deep subspace clustering model builds an affinity matrix $\boldsymbol {\mathit {\Lambda }}$ for spectral clustering [70] by Eq. (13):


(13) \begin{eqnarray*}
\boldsymbol {\mathit {\Lambda }}=\frac{1}{2}\left(\left|\boldsymbol {C}_{F}\right|+\left|\boldsymbol {C}_{F}^{\mathrm{T}}\right|\right)
\end{eqnarray*}

Consequently, the clustering result $\boldsymbol {Y}_{clu}$ can be obtained by spectral clustering based on $\boldsymbol {\mathit {\Lambda }}$.

In particular, the multiscale self-expression loss is represented as Eq. (14):


(14) \begin{eqnarray*}
\begin{split} &\mathcal {L}_{m s s}=\min _{\boldsymbol {C}^{(k)}} \frac{1}{2 L} \cdot \sum _{k=1}^{L}\left\Vert \boldsymbol {Z}^{(k)}-\boldsymbol {C}^{(k)} \boldsymbol {Z}^{(k)}\right\Vert _{F}^{2} \\
&\text{ s.t. } \quad \left(\operatorname{diag}\left(\boldsymbol {C}^{(k)}\right)=0\right) \end{split}
\end{eqnarray*}

Besides, a regularization loss is introduced to avoid $\boldsymbol {C}^{(k)}$ being too sparse:


(15) \begin{eqnarray*}
\begin{split} &\mathcal {L}_{\text{reg }}=\min _{\boldsymbol {C}^{(k)}} \frac{1}{L} \cdot \sum _{k=1}^{L}\left\Vert \boldsymbol {C}^{(k)}\right\Vert _{p} \\
&\text{ s.t. } \quad \left(\operatorname{diag}\left(\boldsymbol {C}^{(k)}\right)=0\right) \end{split}
\end{eqnarray*}

Thus, the total loss in the multiscale self-expression module is denoted as Eq. (16):


(16) \begin{eqnarray*}
\mathcal {L}_{2}=\mathcal {L}_{mss}+\mathcal {L}_{reg}
\end{eqnarray*}

#### Spot robust latent feature learning

Furthermore, distinct from [47], we employ a self-supervised module to learn spot robust latent features. First, spots are classified based on 3 full connection layers. Let the dimensions of all full connection layers be denoted as $\left\lbrace d_{L} \times D_{1} \times D_{2} \times D_{3} \times m\right\rbrace $, where $d_{L}$ denotes the dimension of $\boldsymbol {Z}^{(L)}$, and $D_1$, $D_2$, and $D_3$ denote the dimensions of 3 full connection layers, respectively. We obtain the classification results $\boldsymbol {P}\in R^{n \times m}$ of *n* spots based on the 3 full connection layers.

Next, we use the cross-entropy loss between the classification results $\boldsymbol {P}$ and the clustering results $\boldsymbol {Y}_{clu}$ to constrain a self-supervised learning module by Eq. (17):


(17) \begin{eqnarray*}
\mathcal {L}_{3}=\mathcal {L}_{{sup}}=\min _{\boldsymbol {P}}-\sum _{i=1}^{n} \sum _{j=1}^{m} \boldsymbol {P}(i, j) \log \boldsymbol {Y}_{c l u}(i, j)
\end{eqnarray*}

where $\boldsymbol {Y}_{c l u}(i, j)$ denotes the *j*th clustering label of spot *i* obtained from spectral clustering, and $\boldsymbol {P}(i, j)$ denotes the *j*th classification label of spot *i* based on 3 full connection layers.

Finally, by integrating Eqs. (9), (14), (15), and (17), the total loss function of multiscale GATE is denoted as Eq. (18):


(18) \begin{eqnarray*}
\begin{split} \mathcal {L}_{total }=\min _{(\boldsymbol {C}, \boldsymbol {P}, \boldsymbol {Z})} \mathcal {L}_{1}+ \mathcal {L}_{reg }+\lambda \cdot \mathcal {L}_{mss }+\mathcal {L}_{sup } \end{split}
\end{eqnarray*}

where $\lambda$ is a trade-off parameter used to measure the importance of $\mathcal {L}_{mss}$.

### Biological application

STMSGAL first identifies spatial domains using Leiden clustering [71], Louvain clustering [40] or mclust clustering [72] based on the obtained spot embedding feature matrix. Second, it implements differential expression analysis using the *t*-test in the Scanpy package. Finally, it conducts trajectory inference.

#### Spatial clustering

Based on the learned spot embedding feature matrix, we use different strategies to identify spatial domains. For the DLPFC dataset, mclust clustering [72] is applied to spatial clustering. For other datesets, Louvain or Leiden clustering [40, 71] is used to implement ST clustering.

In addition, although the spot embedding feature matrix is obtained by integrating both gene expressions and spatial contexts, several spots may be incorrectly assigned to spatially diametrical domains, which may cause noise and influence downstream analysis. To solve this problem, an optional optimization step is used to further optimize spatial clustering results obtained from Louvain clustering on the DLPFC dataset: for a given spot *i*, its surrounding spots within an *r* radius circle are taken as its neighbors. Next, we reassign *i* to a spatial domain with the most frequent label of its neighbors. In addition, the clustering results are visualized using UMAP [73].

#### Differential expression analysis

Differential expression analysis is one primary downstream analysis method on transcriptomic data [74–76]. It helps identify biomarkers for novel cell types or detect gene signatures for cellular heterogeneity, and it further provides data for other secondary analyses (such as gene set or pathway analysis and network analysis). We use the *t*-test implemented in the SCANPY package [66] to identify differentially expressed genes for spatial domains.

#### Trajectory inference

ST technologies help depict tissues and organisms in great detail. Tracking the transcriptomic profiles of cells over time and studying their dynamic cellular process contribute to the computational reconstruction of cellular developmental processes. Trajectory inference enables us to better study the potential dynamics of a query biological process, for example, cellular development, differentiation, and immune responses [77]. It can detect a graph-like structure existing in the dynamic process from the sampled cells. Properties of cells are compared over pseudotime [78] by mapping them to the captured structure. Trajectory inference allows us analyze how cells evolve from one cell state to another, as well as when and how cells should make cell fate decisions. In this section, the PAGA algorithm [79] in the SCANPY package [66] is employed to depict the spatial trajectory. The obtained trajectory figures are visualized using the *scanpy.pl.paga_compare*() function.

## Results

### Experimental setting

In STMSGAL, both the encoder and the decoder with the activation function of the exponential linear unit (ELU) [80] included neural networks with 2 graph attention layers, where the number of neurons was 512 and 30, respectively. The Adam optimizer [81] was employed to minimize their reconstruction loss. In the self-supervised module, the activation function was set to rectified linear units (ReLu) [82]. For Louvain clustering, the radius *r* was set to 50 when STMSGAL obtained the best clustering performance on the DLPFC dataset.

STMSGAL adopted the same data preprocessing as those of SCANPY. Both used log-normalization and constructed the nearest neighbor network. SCANPY obtained spatial clustering with the *scanpy.tl.louvain*() function. Table 1 shows parameter settings of STMSGAL on 5 ST datasets. For each dataset with labels, the resolution parameter was tuned manually to ensure the cluster number was equal to the ground truth. Thus, the cluster number in each method was set to the same as one of ground truth layers. For other clustering methods, we adopted their default settings.

**Table 1:** Parameter settings

Datesets	Parameter settings
DLPFC	rad_cutoff = 150
	cost_ssc = 0.1
	$\alpha$ = 0
	method = ‘Louvain’
Human Breast Cancer	rad_cutoff = 300
(Block A, Section 1)	cost_ssc = 1
	$\alpha$ = 0.7
	method = ‘leiden’
Adult Mouse Brain	rad_cutoff = 300
(FFPE)	cost_ssc = 0.1
	$\alpha$ = 0.5
	method = ‘Louvain’
Human Breast Cancer	rad_cutoff = 300
(DCIS)	cost_ssc = 1
	$\alpha$ = 0.5
	method = ‘Louvain’
Mouse visual cortex	rad_cutoff = 400
	cost_ssc = 0.1
	$\alpha$ = 0
	method = ‘mclust’
Stereo-seq mouse embryo	rad_cutoff = 3
	cost_ssc = 0.1
	$\alpha$ = 0
	method = ‘Louvain’

### Evaluation metrics

For 3 datasets with labels (Human Breast Cancer [Block A, Section 1], DLPFC, and mouse visual cortex STARmap), we employed adjusted Rand index (ARI) [83] to evaluate the performance of different spatial clustering algorithms. ARI computes the similarity between the predicted clustering labels and reference cluster labels by Eq. (19):


(19) \begin{eqnarray*}
ARI=\frac{RI-E[RI]}{\mathrm{max}(RI)-E[RI]}
\end{eqnarray*}

where the unadjusted Rand index is $RI=(a+b) / C_{n}^{2}$, where $a$ and $b$ indicate the number of pairs correctly labeled in the same dataset and not in the same dataset, respectively. $C_{n}^{2}$ indicates the total number of possible pairs. $E[RI]$ indicates the expected $RI$ based on random labeling. A higher $\mathrm{ARI}$ score denotes better performance.

For 2 datasets whose spatial domain annotations are unavailable (Adult Mouse Brain [FFPE] and Human Breast Cancer [DCIS]), we evaluated the performance of spatial clustering algorithms based on 3 clustering metrics: Davies–Bouldin (DB) score [84], Calinski–Harabasz (CH) score [85], and S_Dbw score [86, 87]. DB was computed by averaging all cluster similarities, where the similarity between each cluster and its most similar cluster was taken as its cluster similarity. The similarity was computed by the ratio of within-cluster distances to between-cluster distances. CH is used to measure the cluster validity by averaging the squares of within- and between-cluster distance sum of all spots. S_Dbw evaluates intraclass compactness and interclass density of each spot. Small DB and S_Dbw and large CH indicate the optimal cluster clustering.

### Performance comparison of STMSGAL with 6 other methods on 2 datasets without labels

To investigate the clustering performance of STMSGAL, we compared it with 6 other clustering algorithms—that is, SCANPY [66], SEDR [45], CCST [46], STAGATE [47], DeepST [49], and GraphST [50]—on two 10x Genomics Visium datasets without labels (i.e., Adult Mouse Brain [FFPE] and Human Breast Cancer [DCIS]). The former one method obtained broad applications in single-cell clustering, and the remaining 5 methods were widely applied to spatial clustering. Table 2 shows the DB, CH, and S_Dbw scores computed by STMSGAL and other methods on the above 2 datasets. The best performance in each column was denoted using the bold font. The results demonstrated that STMSGAL computed the smallest DB and S_Dbw and the highest CH on Adult Mouse Brain (FFPE) and the highest CH and the smallest S_Dbw on Human Breast Cancer (DCIS), suggesting its optimal clustering performance.

**Table 2:** Performance comparison of STMSGAL with 6 other clustering methods on Adult Mouse Brain (FFPE) and Human Breast Cancer (Ductal Carcinoma In Situ [DCIS])

		Metrics		
Datasets	Methods	DB	CH	S_Dbw
Adult Mouse Brain	SCANPY	1.442	358.67	0.481
(FFPE)	SEDR	1.951	84.569	0.652
	CCST	1.173	507.421	0.453
	DeepST	1.166	842.033	0.328
	STAGATE	1.467	495.547	0.427
	GraphST	1.470	310.860	0.501
	STMSGAL	**1.155**	**1,010.724**	**0.311**
Human Breast Cancer	SCANPY	2.069	379.084	0.593
(DCIS)	SEDR	2.627	54.778	0.742
	CCST	1.469	507.421	0.453
	DeepST	**1.263**	611.567	0.48
	STAGATE	1.916	430.630	0.587
	GraphST	1.951	369.594	0.610
	STMSGAL	1.451	**1,190.850**	**0.332**

* The bold font indicates the best performance in each column. Lower Davies–Bouldin (DB) and S_Dbw and higher Calinski–Harabasz (CH) denote better performance.

### STMSGAL demonstrates robust clustering performance across ST datasets with different spatial resolutions

To evaluate the STMSGAL performance on spatial domain identification, we compared it with existing 7 state-of-the-art methods on 4 DLPFC sections. Particularly, in complex networks, nodes are clustered into relatively dense communities through the clustering algorithm. Louvain clustering is a nonspatial clustering algorithm. It assigns each spot to a significantly differential community and achieves the desired clusters by iteratively merging and splitting communities. It exhibits powerful clustering performance compared with spectral clustering when clustering ST data, such as DLPFC. Thus, we used the Louvian clustering for performing clustering again on DLPFC.

**Figure 2:**
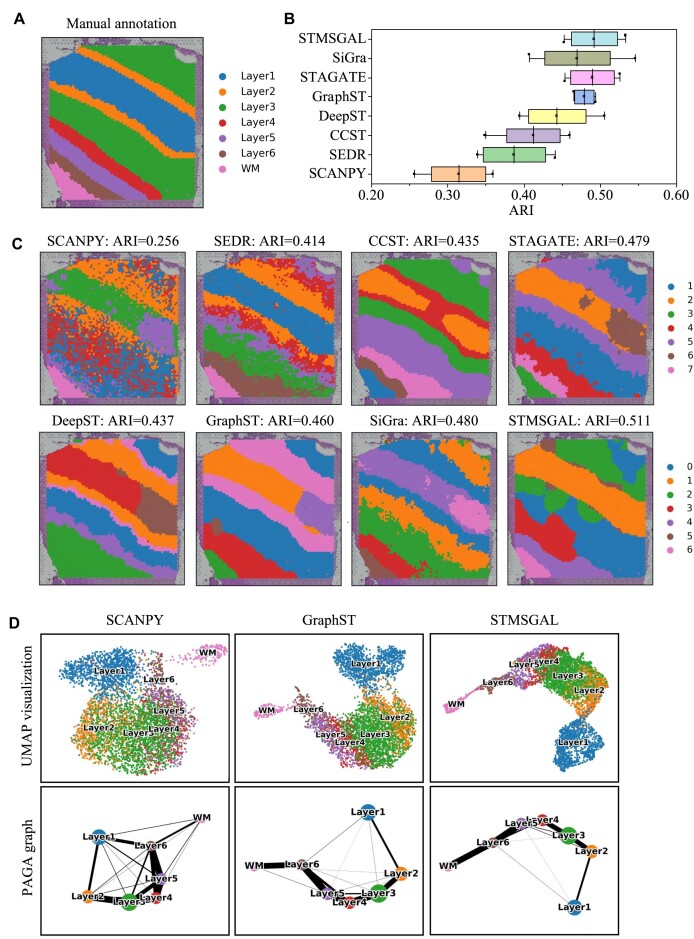
STMSGAL improves the identification of layer structures in the DLPFC tissue. (A) Ground-truth segmentation of 6 cortical layers and 1 white matter layer in the DLPFC section 151509. (B) Boxplots of ARI computed by STMSGAL and other 7 methods in the DLPFC sections, from 151507 to 151510. (C) Cluster assignments generated by SCANPY, SEDR, CCST, STAGATE, DeepST, GraphST, SiGra, and STMSGAL in the DLPFC section 151509. (D) UMAP visualizations and PAGA graphs generated by SCANPY, GraphST, and STMSGAL embeddings in the DLPFC section 151509.

Moreover, the DLPFC dataset provides high-resolution images and satisfies the need of spatial clustering methods, including SiGra, that must combine high-resolution images for clustering ST data. The results elucidated that spatial domains captured by STMSGAL were consistent with manual annotation on human DLPFC sections and the definition of cortical stratification in neuroscience (Fig. 2).

In addition, STMSGAL effectively captured the expected cortical layer structures and significantly improved spatial clustering performance in comparison with SCANPY, SEDR, CCST, STAGATE, SiGra, DeepST, and GraphST (Fig. 2 and Supplementary Fig. S1). For average ARIs, STMSGAL achieved the best performance (Fig. 2B). In the DLPFC section 151509, STMSGAL clearly depicted the layer borders and obtained the best average ARI of 0.511. In the section, although the clustering results of SCANPY roughly adhered to the expected layer structures, its cluster boundary was discontinuous with many noises, which greatly influenced its clustering accuracy. Moreover, SCANPY is a nonspatial clustering algorithm, and SEDR, CCST, DeepST, STAGATE, SiGra, and GraphST are spatial clustering algorithms. Interestingly, the performance of the above 6 spatial clustering algorithms, especially STMSGAL, is better than the clustering method, elucidating STMSGAL’s powerful spatial domain identification ability (Fig. 2C).

STMSGAL manifested the distance between spatial domains and characterized the spatial trajectory in a UMAP plot [73] by integrating spatial contexts. For example, in the DLPFC section 151509, the UMAP plots delineated by STMSGAL embeddings elucidated well-organized cortical layers and consistent spatial trajectories, which was in accord with functional similarity between adjacent cortical layers and the chronological order [88]. Furthermore, in the UMAP plots delineated by SCANPY embeddings, spots that belong to different layers were not clearly divided while GraphST and STMSGAL could well divide most spots into different layers (Fig. 2D). Finally, we used a trajectory inference approach named PAGA [79] to verify the inferred trajectory. The PAGA graphs depicted by both STMSGAL and GraphST embeddings had a approximately linear development trajectory from layer 1 to layer 6. In addition, the identified adjacent layers by STMSGAL and GraphST showed similarity while ones from SCANPY embeddings were mixed (Fig. 2D).

**Figure 3:**
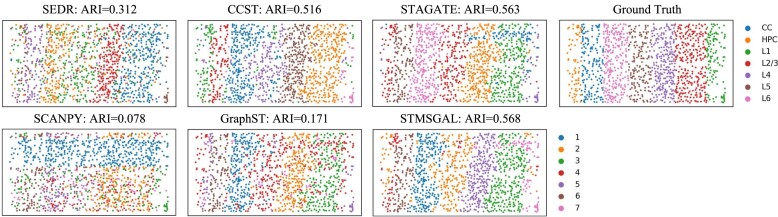
Spatial domains identified by SCANPY, SEDR, CCST, GraphST, STAGATE, and STMSGAL in the mouse visual cortex STARmap dataset.

We further evaluated the performance STMSGAL on the mouse visual cortex STARmap dataset, which is an image-based ST dataset at single-cell resolution and is generated by the STARmap technique [29]. mclust is a widely used R package applied to model-based clustering through finite Gaussian mixture modeling. It is more suitable to single-cell resolution data with fewer samples, such as mouse visual cortex STARmap dataset. Thus, we used mclust for performing clustering again on STARmap. Using the gold standard annotated by experts, as shown in Fig. 3, STMSGAL obtained the best ST clustering performance with an ARI of 0.568 compared to SCANPY, SEDR, CCST, STAGATE, and GraphST, while STAGATE achieved the second-best ranking with ARI of 0.563 (Fig. 3).

We also validated the performance of STMSGAL for identifying tissue structures on the Stereo-seq dataset from mouse embryos at E9.5. Tissue domain annotations of mouse embryos were obtained from [65].

**Figure 4:**
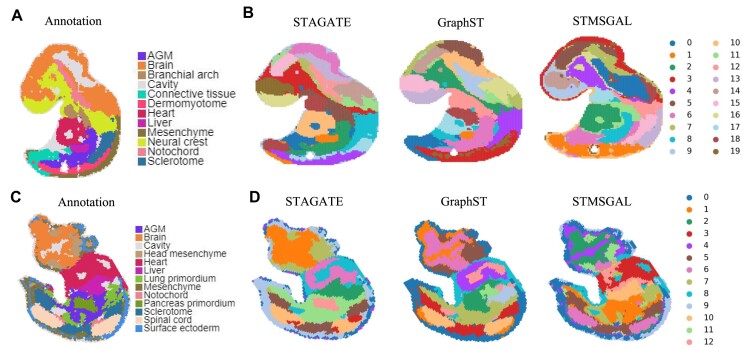
STMSGAL improves accurately identification of different organs in the Stereo-seq mouse embryo. (A) Tissue domain annotations of the E9.5_E1S1 mouse embryo data. (B) Cluster assignments generated by STAGATE, GraphST, and STMSGAL on E9.5_E1S1 mouse embryo data. (C) Tissue domain annotations of the E9.5_E2S2 mouse embryo data. (D) Cluster assignments generated by STAGATE, GraphST, and STMSGAL on the E9.5_E2S2 mouse embryo data.

We investigated the clustering results of STAGATE, GraphST, and STMSGAL on the E9.5_E1S1 embryo. As shown in Fig. 4A, although the original annotation had 12 reference clusters, we set the number of clusters in our testing to 20 to acquire a higher resolution of tissue segmentation. The clusters identified by both STAGATE and STMSGAL matched the annotation well (Fig. 4B). As shown in Table 3, however, compared to STAGATE, STMSGAL computed the smallest DB and S_Dbw and the highest CH.

**Table 3:** Performance comparison of STMSGAL with STAGATE and GraphST on the Stereo-seq mouse embryos

		Metrics		
Datasets	Methods	DB	CH	S_Dbw
E9.5_E1S1	STAGATE	1.579	582.733	0.585
	GraphST	**1.396**	686.177	0.549
	STMSGAL	1.957	**1,915.695**	**0.355**
E9.5_E2S2	STAGATE	1.708	603.290	0.608
	GraphST	**1.686**	563.371	0.632
	STMSGAL	1.861	**1,171.402**	**0.488**

* The bold font indicates the best performance in each column. Lower Davies–Bouldin (DB) and S_Dbw and higher Calinski–Harabasz (CH) denote better performance.

Moreover, we compared the clustering results of STAGATE, GraphST, and STMSGAL on the E9.5_E2S2 mouse embryo. Here, we set the number of clusters to 13, matching the original annotation (Fig. 4C). The results demonstrated that STMSGAL computed the smallest DB and S_Dbw and the highest CH (Table 3). STAGATE produced more smoother clusters but failed to reveal any fine-grained tissue complexity (Fig. 4D). For example, STAGATE failed to identify cavity in the brain (domain 2). In contrast, STMSGAL’s clusters better matched the annotated regions.

### STMSGAL can accurately dissect spatial domains on 2 breast cancer tissues

Differed from the cerebral cortex with clear and known morphological boundaries, breast cancer tissues are remarkably heterogeneous and consist of a complex tumor microenvironment. Consequently, manually labeling cancer data only via tumor morphology cannot fully depict the complexity. Thus, we utilized STMSGAL to find spatial domains on two 10x Genomics Visium datasets with respect to Human Breast Cancer (Block A, Section 1) and Human Breast Cancer (DCIS).

Particularly, Louvain clustering may produce arbitrarily badly connected communities. In the worst case, the obtained communities may even be discontinuous, especially when performing clustering iteratively. Moreover, due to the limitation of resolution, smaller communities may be clustered into larger communities. That is, smaller communities may be hidden, resulting in obtained communities containing significant substructures.

Leiden clustering is a modified version of Louvain clustering and can yield well-connected communities based on the smart local move strategy. Cancer tissues with tumor heterogeneity contain many small substructures. Thus, we used the Leiden clustering for cancer tissues with tumor heterogeneity, such as human breast cancer.

**Figure 5:**
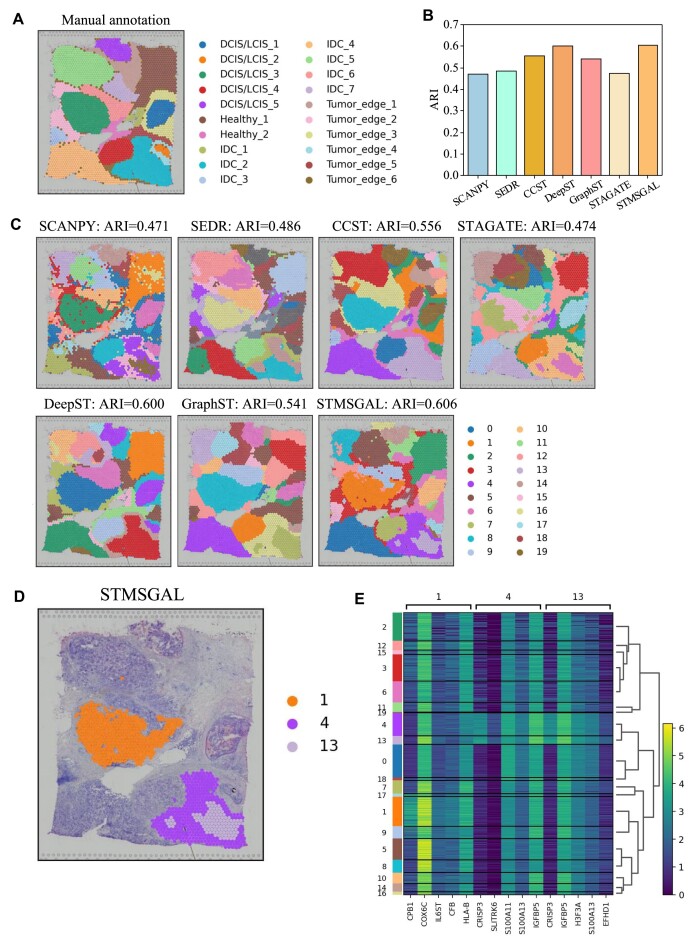
STMSGAL can accurately dissect spatial domains on Human Breast Cancer (Block A, Section 1). (A) Manual pathology labeling via hematoxylin and eosin staining. (B) The average ARI values computed by SCANPY, SEDR, CCST, STAGATE, DeepST, GraphST, and STMSGAL on Human Breast Cancer (Block A, Section 1). (C) Cluster assignments generated by SCANPY, SEDR, CCST, STAGATE, DeepST, GraphST, and STMSGAL on Human Breast Cancer (Block A, Section 1). (D) Spatial domains identified by STMSGAL. (E) Heatmap of the top 5 differentially expressed genes of domains 1, 4, and 13 on Human Breast Cancer (Block A, Section 1).

Human Breast Cancer (Block A, Section 1) data have obvious intratumoral and intertumoral differences. It was manually annotated by SEDR [45] (Fig. 5A) and divided into 20 regions. It contains 4 main morphotypes: ductal carcinoma in situ/lobular carcinoma in situ (DCIS/LCIS), invasive ductal carcinoma (IDC), tumor surrounding regions with low features of malignancy (tumor edge), and healthy tissue (healthy).

We compared the clustering accuracy of STMSGAL with SCANPY [66], SEDR [45], CCST [46], STAGATE [47], DeepST [49], and GraphST [50] in terms of average ARI. The results show that STMSGAL computed the best ARI, significantly outperforming 5 other clustering methods (Fig. 5B).

Figure 5C shows spatial domains identified by SCANPY, SEDR, CCST, STAGATE, DeepST, GraphST, and STMSGAL. The results demonstrate that the identified domains by STMSGAL were highly consistent with manual annotations in Fig. 5A and had more regional continuity. In addition, compared with other methods, STMSGAL obtained the best clustering accuracy with an ARI of 0.606. Furthermore, STMSGAL identified several subclusters within the tumor regions, such as spatial domains 4 and 13 (Fig. 5D). Furthermore, STMSGAL identified some spatial domains with low heterogeneity (i.e., healthy regions) that were remarkably consistent with the manual annotations in Fig. 5A.

We also analyzed intratumoral transcriptional differences among domains 1 (DCIS/LCIS), 4, and 13 (IDC) based on differential expression analysis (Fig. 44E). In domain 1, we identified 3 differentially expressed genes, that is, *CPB1, COX6X*, and *IL6ST. CPB1* can obviously differentiate DCIS from the other subtypes of breast cancer [89]. *COX6X* may help the differentiation between estrogen receptor–positive and estrogen receptor–negative subtypes [90]. The expression of *IL6ST* is closely associated with a lower risk of invasion, metastasis, and recurrence [91]. In domains 4 and 13, 2 differentially expressed genes, *IGFBP5* and *CRISP3*, have dense linkages with the treatment of mammary carcinoma [92, 93]. The knockdown of *CRISP3* can greatly inhibit the migration and invasion of mammary carcinoma cells and the *ERK1/2 MAPK* signaling pathway. *CRISP3* was also considered a marker for clinical outcomes in patients with mammary carcinoma [92]. *IGFBP5* helps manage tamoxifen resistance in breast cancer [93]. The above results suggested that STMSGAL can accurately identify spatial regions with different biological functions.

**Figure 6:**
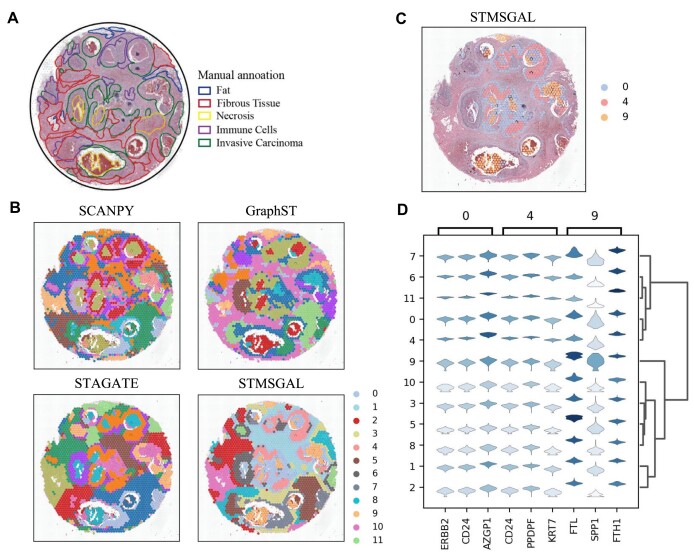
STMSGAL can accurately dissect spatial domains on Human Breast Cancer (DCIS). (A) Hematoxylin and eosin staining figures annotated by Agoko’s telepathology platform on Human Breast Cancer (DCIS). (B) Spatial domains identified by SCANPY, GraphST, STAGATE, and STMSGAL on Human Breast Cancer (DCIS). (C) Spatial domains 0, 4, and 9 identified by STMSGAL. (D) Stacked violin plots illustrate the top 3 differentially expressed genes on spatial domains 0, 4, and 9 and their expressions on all spatial domains.

We further investigated ST data on Human Breast Cancer (DCIS). Figure 6A gives its manually annotated areas. STMSGAL identified more fluent and continuous regions than other algorithms and better matched the annotated areas (Fig. 6B, Supplementary Fig. S2, and Table 2). Figure 6D lists the top 3 differentially expressed genes (i.e., *AZGP1, CD24*, and *ERBB2*) in domain 0 (Fig. 6C). The expression of *AZGP1* determines the histologic grade of tumors in breast cancer [94]. *CD24* is a key indicator of triple-negative breast cancer [95–97]. In particular, the overexpression of *ERBB2* categorizes *ERBB2/HER2*-positive, a subclass of breast cancer. The subclass accounts for about 20–30% among all types of breast malignancies and is usually linked to poor prognosis [98]. Targeting *ERBB2* contributes to the treatment of *ERBB2*-positive breast cancers [99].

### STMSGAL helps to better delineate the similarity between neighboring spots on Adult Mouse Brain (FFPE)

**Figure 7:**
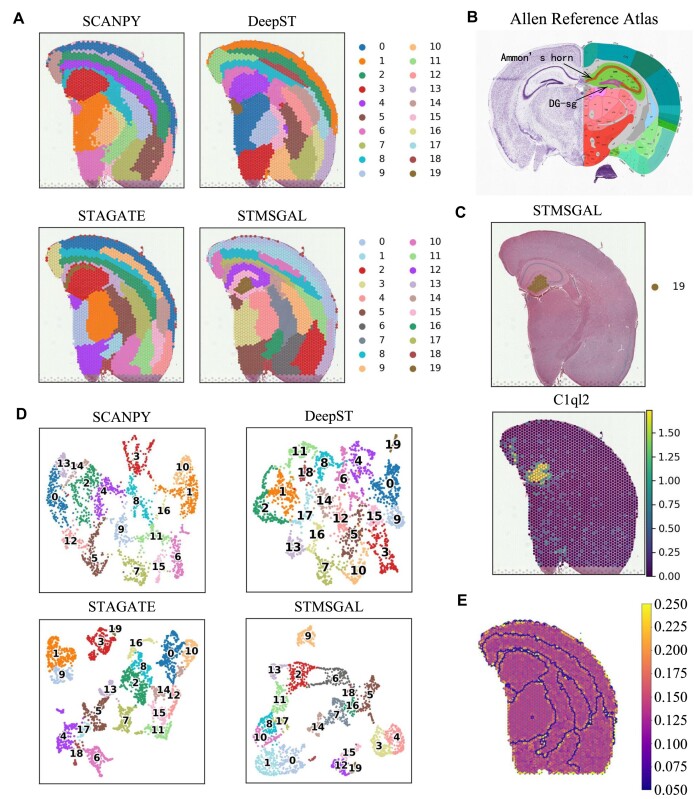
STMSGAL reveals spatial domains on Adult Mouse Brain (FFPE). (A) Spatial domains identified by SCANPY, DeepST, STAGATE, and STMSGAL. (B) The annotation of hippocampus structures from the Allen Reference Atlas on mouse brain. (C) Visualization of domains identified by STMSGAL and the corresponding marker genes. (D) UMAP visualization generated by SCANPY, DeepST, STAGATE, and STMSGAL embeddings, respectively. (E) Visualization of all attention layers of STMSGAL with the ctaSNN module. In each attention layer, nodes were arranged based on spatial contexts of spots, and edges were colored by corresponding weights.

STMSGAL was still applied to provide insights into more complex tissues on a 10x Genomics Visium dataset from Adult Mouse Brain (FFPE) (Fig. 7 and Supplementary Fig. S3). Figure 7A shows spatial domains identified by SCANPY, DeepST, STAGATE, and STMSGAL. In the hippocampal region, the clustering results generated by SCANPY roughly separated the brain tissue structures composed of different cell types but failed to capture small spatial domains. SCANPY did not observe the “cord-like” structure (i.e., Ammon’s horn) and the “arrow-like” structure (i.e., dentate gyrus) within the hippocampus. DeepST only smoothed the spatial domain boundaries but failed to delineate small spatial domains. STMSGAL without ctaSNN captured Ammon’s horn but did not characterize smaller spatial domains. However, STMSGAL with ctaSNN clearly identified both Ammon’s horn and dentate gyrus structures in the hippocampus, in accord with annotations about the hippocampus structures from the Allen Reference Atlas [100] (Fig. 7B). The above results suggested that STMSGAL significantly improved spatial domain identification. Furthermore, even for ST data composed of heterogeneous cell types with low spatial resolution, STMSGAL with ctaSNN can still accurately decipher the spatial similarity.

Additionally, the expressions of multiple known gene markers validated the cluster partitions of STMSGAL (Fig. 7C and Supplementary Fig. S4). For example, *C1ql2* was highly expressed on the identified DG-sg region [101]. *Hpca*, which mediates calcium-dependent translocation of brain-type creatine kinase in hippocampal neurons, was highly expressed in Ammon’s horn region [102]. Notably, STMSGAL also captured several well-separated spatial domains and deciphered their spatial expression patterns based on differential expression analysis. Domain 15 within the hippocampus, except for the “cord-like” and “arrow-like” structures, delineated high expressions of 2 astrocyte gene markers ${\it Mt2}$ and ${\it Gfap}$ [103]. The spatial domain 14 surrounding the hippocampus expressed multiple oligodendrocyte-related gene markers, including ${\it Trf}$ and ${\it Mbp}$ [104] (Supplementary Fig. S4). The above results elucidated that STMSGAL can efficiently detect spatial heterogeneity and further decompose spatial expression patterns. Notably, the cell type–aware module obviously boosted the partition of tissue structures on Adult Mouse Brain (FFPE) based on its UMAP plot [73], while those of DeepST were more like a smooth version of the nonspatial method SCANPY (Fig. 7D).

Finally, all attention layers of STMSGAL with ctaSNN were visualized. In each layer, nodes were arranged based on spot spatial locations, and edges were colored by corresponding weights. The results demonstrated that the combination of attention mechanism and ctaSNN boosted the characterization of the boundaries of main tissue structures on Adult Mouse Brain (FFPE) (such as the cortex, hippocampus, and midbrain) (Fig. 7E). Collectively, attention mechanism and ctaSNN contributed to delineating the similarity between neighboring spots (Fig. 7E).

## Ablation Study

In our STMSGAL method, the combination of a graph attention autoencoder (GATE) and multiscale deep subspace clustering aims to obtain multiscale feature information of spots. The self-supervised module aims to learn robust latent features with clustering information for each spot.

To justify the contribution and necessity of these components, we conducted the ablation study to further investigate the effects of GATE, multiscale deep subspace clustering, and the self-supervised module on spatial clustering performance on the DLPFC sections from 151507 to 151510. As shown in Table 4, $\mathcal {L}_{1}$ denotes the reconstruction loss of normalized expressions based on GATE. $\mathcal {L}_{2}$ denotes the loss of the multiscale deep subspace clustering module, which contains regularization loss $\mathcal {L}_{reg}$ and multiscale self-expression loss $\mathcal {L}_{mss}$, and $\mathcal {L}_{3}$ is the loss of the self-supervised module.

**Table 4:** Ablation study on different loss terms

	Loss function			
Datasets	$\mathcal {L}_{1}$	$\mathcal {L}_{2}$	$\mathcal {L}_{3}$	ARI
151507	$\circ$	$\times$	$\times$	0.508
	$\circ$	$\circ$	$\times$	0.518
	$\circ$	$\circ$	$\circ$	**0.533**
151508	$\circ$	$\times$	$\times$	0.405
	$\circ$	$\circ$	$\times$	0.444
	$\circ$	$\circ$	$\circ$	**0.473**
151509	$\circ$	$\times$	$\times$	0.392
	$\circ$	$\circ$	$\times$	0.447
	$\circ$	$\circ$	$\circ$	**0.511**
151510	$\circ$	$\times$	$\times$	0.437
	$\circ$	$\circ$	$\times$	0.442
	$\circ$	$\circ$	$\circ$	**0.452**

* The bold type indicates the best performance in each column.

From Table 4, we found that both the multiscale deep subspace clustering module and the self-supervised module cooperated well with GATE and greatly improved the clustering performance. The results demonstrated that the self-supervised module, which utilized the clustering labels to self-supervise the learning of spot embeddings, obtained more accurate clustering ability. The multiscale deep subspace clustering module fully utilized the embedded multiscale information and manifested an obvious effect on spatial clustering, suggesting that a proper clustering-oriented loss function can efficiently enhance the clustering performance.

Moreover, to analyze the effect of the multiscale strategy on spatial clustering performance, we compared the difference between individual self-expression layers and multiscale self-expression layers. Table 5 gives the ARI values of STMSGAL with or without the multiscale strategy for the DLPFC sections from 151507 to 151510. We applied a controlled variable approach to make the rest of the modules the same. The results indicated that the performance of STMSGAL with the multiscale strategy was better than one from a single self-expression layer on the 4 DLPFC sections, verifying that the multiscale strategy fully utilized the embedding features in different layers. In addition, the adaptive fusion method still significantly improved the spatial clustering performance.

**Table 5:** Ablation study on the multiscale strategy

Datasets	Strategy	ARI
151507	Without the multiscale strategy	0.485
	With the multiscale strategy	**0.533**
151508	Without the multiscale strategy	0.394
	With the multiscale strategy	**0.473**
151509	Without the multiscale strategy	0.437
	With the multiscale strategy	**0.511**
151510	Without the multiscale strategy	0.407
	With the multiscale strategy	**0.452**

* The bold type indicates the best performance in each column.

Since some spots could be erroneously assigned to spatially diametrical domains and cause noise during spot embedding feature learning, we used an additional optimization step to further optimize spatial clustering results obtained from Louvain clustering on the DLPFC dataset.

To further investigate the effect of the additional optimization step on the spatial clustering performance, we compared the performance of STMSGAL with or without the additional optimization step for sections 151507 to 151510 of DLPFC. Table 6 gives the ARI values of STMSGAL with or without the additional optimization step for DLPFC. The results demonstrated that STMSGAL with the additional optimization step significantly outperformed STMSGAL without the step. Thus, the additional optimization step could help spatial clustering.

**Table 6:** Ablation study on the additional optimization step

Datasets	Strategy	ARI
151507	Without the additional optimization step	0.509
	With the additional optimization step	**0.533**
151508	Without the additional optimization step	0.450
	With the additional optimization step	**0.473**
151509	Without the additional optimization step	0.484
	With the additional optimization step	**0.511**
151510	Without the additional optimization step	0.430
	With the additional optimization step	**0.452**

* The bold type indicates the best performance in each column.

When performing clustering again, we used Louvian clustering on DLPFC, Leiden clustering on Human Breast Cancer, and mclust on STARmap. To analyze why different clustering algorithms were used on different datasets, we conducted ablation experiments on the above 3 datasets. Tables 7 and 8 demonstrated ablation analysis results based on different clustering methods when performing clustering again on DLPFC 10x Genomics Visium datasets, STARmap, and Human Breast Cancer (Block A, Section 1), respectively. The results demonstrated that STMSGAL significantly improved ST clustering accuracy when using Louvian clustering on DLPFC, Leiden clustering on Human Breast Cancer, and mclust on STARmap.

**Table 7:** Ablation analysis under different clustering methods on DLPFC 10x Genomics Visium datasets

	Datasets				
Methods	151507	151508	151509	151510	Average ARI
Louvain clustering	**0.533**	0.473	**0.511**	**0.452**	**0.492**
mclust	0.520	0.475	0.354	0.403	0.438
Leiden clustering	0.511	**0.489**	0.471	0.393	0.469
Subspace clustering	0.216	0.325	0.395	0.284	0.294

* The bold type indicates the best performance in each column.

**Table 8:** Ablation analysis under different clustering methods on STARmap and Human Breast Cancer (Block A, Section 1)

Datasets	Methods	ARI
STARmap	Louvain clustering	0.282
	mclust	**0.568**
	Leiden clustering	0.273
	Subspace clustering	0.067
Human Breast Cancer	Louvain clustering	0.534
(Block A, Section 1)	mclust	0.512
	Leiden clustering	**0.606**
	Subspace clustering	0.588

* The bold type indicates the best performance in each column.

## Discussion

Accurately detecting spatial domains and identifying differentially expressed genes can greatly boost our understanding about tissue organization and biological functions. In this article, we developed a spatial domain identification framework called STMSGAL based on GATE and multiscale deep subspace clustering. STMSGAL can been accurately incorporated to the standard analysis pipeline by using the “anndata” object in the SCANPY package [66] as inputs.

Different from classical autoencoders, STMSGAL utilized an attention mechanism in multiple hidden layers of the encoder and decoder. First, it constructed ctaSNN through Louvain clustering exclusively based on gene expression profiles. The weights of edges in the ctaSNN depicted the similarity between neighboring spots and were adaptively learned. Next, it integrated expression profiles and the constructed ctaSNN to form spot latent embedding representation based on GATE. It mainly includes spot embedding feature matrix construction, subspace clustering combining self-expression coefficient learning and affinity matrix construction, and spot robust latent feature learning based on self-supervised learning. Finally, it implemented biological applications, including spot clustering, differential expression analysis, and trajectory inference.

In the STMSGAL method, the multiscale self-expression module was used to fully explore the associations between spot representations in all encoder layers. The deep subspace clustering module was utilized to obtain the clustering labels for each spot through a clustering-oriented loss function. The self-supervised module was introduced to effectively learn spot latent representation. The combination of the above 3 modules helps to learn more discriminative features with clustering information for each spot. The more discriminative features obtained with clustering information were used as the input of spectral clustering and conducted the final clustering.

Traditional subspace clustering mainly contains 2 procedures: constructing an affinity matrix through representation learning and spectral clustering. However, spectral clustering is sensitive to the construction of a similarity matrix and the selection of various parameters, but the Leiden/Louvain/mclust clustering methods are more appropriate to biological data and exhibit a powerful spatial clustering performance. Consequently, Leiden/Louvain/mclust clustering has been widely used in the field of spatial clustering. Thus, our proposed STMSGAL framework used Leiden/Louvain/mclust for performing clustering again to identify spatial domains after obtaining more discriminative features with clustering information based on multiscale deep subspace clustering.

We compared the performance of STMSGAL with 7 other clustering methods on four 10x Genomics Visium datasets from Adult Mouse Brain (FFPE), Human Breast Cancer (DCIS), Human Breast Cancer (Block A, Section 1), and the DLPFC tissues, as well as 1 mouse visual cortex STARmap dataset. The 7 comparison methods include SCANPY, GraphST, SEDR, CCST, STAGATE, DeepST, and SiGra. The SCANPY has been widely applied to single-cell clustering. The remaining are state-of-the-art spatial clustering methods. The results demonstrated that our proposed STMSGAL method obtained impressive performance over other competing methods in terms of 4 evaluation metrics (i.e., DB, CH, S_Dbw, and ARI). STMSGAL significantly improved the identification of layer structures in 4 DLPFC sections, mouse visual cortex STARmap data, and mouse embryo data; accurately dissected spatial domains on 2 breast cancer tissues; and efficiently depicted the similarity between neighboring spots on Adult Mouse Brain (FFPE).

STMSGAL greatly boosted ST data analysis. It may be mainly attributed to the following features: first, although existing methods (such as stLearn) took histological images as inputs, they achieved limited performance. For example, stLearn adopted a pretrained neural network to obtain spot features from images and further computed their morphological distances via cosine distance. However, the predefined strategy in stLearn was not flexible and resulted in its poor spatial clustering performance. In contrast, STMSGAL adopted an attention mechanism to adaptively integrate spatial locations and gene expression profiles.

Second, a multiscale self-expression module was designed to train a self-expression coefficient matrix in different encoder layers. SEDR and CCST merely adopted the representations in the encoder final hidden layer for spatial clustering tasks, wasting much useful information embedded in its other layers. Comparatively, the multiscale self-expression module fully explored the associations between node representations in all encoder layers. Thus, it fully adopted the embedded multiscale information and obtained a more distinct self-expression coefficient matrix. Furthermore, it mapped these features into a more precise subspace for spatial clustering.

Finally, a deep subspace clustering module was proposed to obtain the clustering labels with a clustering-oriented loss function, and a self-supervised module was introduced to effectively guide spot latent representation learning. Thus, the learned spot latent embedding representation greatly improved the clustering performance.

In summary, STMSGAL is a powerful spatial clustering framework that constructs an integrated representation for spots by aggregating both transcriptomic data and spatial context. STMSGAL derived low-dimensional embedding, enabling to conduct spatial clustering and trajectory inference more accurately. Moreover, STMSGAL facilitates deciphering new principles in a spatially organized context.

Although STMSGAL achieved accurate spatial clustering performance, the deep subspace clustering algorithm can be further developed. In the near future, motivated by the linkages between spatial domain identification and single-cell segmentation used to image-based ST data, we anticipate that STMSGAL can be further extended to a single-cell segmentation task applied to the subcellular resolution technologies. We also hope to enhance its applicability on other datasets generated by new sequencing technologies.

Moreover, self-supervised learning can effectively learn spot representations, but optimizing the spot representations by combining the pseudo labels can affect the convergence of the model. The contrastive learning algorithm is a promising paradigm of the self-supervised learning model. In the future, we will introduce contrastive learning to facilitate spot representation learning and spatial clustering.

Finally, the accumulation of ST data generates spatial omics big data, which pose many technical challenges to data integration and analysis. To enable STMSGAL to deal with larger datasets, we will further alleviate the computational burden of STMSGAL using a graph convolutional network mini-batch or parallel techniques to construct large-scale graphs for spatial omics data.

## Availability of Source Code and Requirements

- Project name: STMSGAL
- Project homepage: https://github.com/plhhnu/STMSGAL
- Operating system(s): Platform independent
- Programming language: Python
- License: MIT license for the code, Creative Commons CC0 1.0 Public Domain Dedication for the filtered spatial transcriptomic data
- RRID: SCR_025422
- biotools: stmsgal

## Supplementary Material

giae103_Supplemental_Files

giae103_GIGA-D-24-00044_Original_Submission

giae103_GIGA-D-24-00044_Revision_1

giae103_GIGA-D-24-00044_Revision_2

giae103_GIGA-D-24-00044_Revision_3

giae103_GIGA-D-24-00044_Revision_4

giae103_Response_to_Reviewer_Comments_Original_Submission

giae103_Response_to_Reviewer_Comments_Revision_1

giae103_Response_to_Reviewer_Comments_Revision_2

giae103_Response_to_Reviewer_Comments_Revision_4

giae103_Reviewer_1_Report_Original_Submission Qianqian Song -- 4/3/2024

giae103_Reviewer_1_Report_Revision_1 Qianqian Song -- 7/24/2024

giae103_Reviewer_2_Report_Original_Submission Zixuan Cang -- 4/8/2024

giae103_Reviewer_2_Report_Revision_1 Zixuan Cang -- 8/7/2024

## Data Availability

Source codes and datasets of STMSGAL are available in the GitHub repository [105]. Specifically, the DLPFC dataset is accessible within the spatialLIBD package [64]. The Adult Mouse Brain (FFPE), Human Breast Cancer (DCIS), and Human Breast Cancer (Block A Section 1) datasets are collected from the 10x Genomics website [61–63]. An archival copy of the code and supporting data are also available via the *GigaScience* repository, GigaDB [106]. DOME-ML (Data, Optimization, Model and Evaluation in Machine Learning) annotations are available via a link in GigaDB [106] and via accession cji1mirt7b in the DOME registry [107].

## Additional Files

**Supplementary Fig. S1**. Comparison of spatial domains identified by SCANPY, SEDR, CCST, DeepST, STAGATE, SiGra, GraphST, and STMSGAL and manual annotations in 3 sections of human DLPFC tissues.

**Supplementary Fig. S2**. Cluster assignments generated by SCANPY, SEDR, CCST, STAGATE, DeepST, GraphST, and STMSGAL on Human Breast Cancer (Ductal Carcinoma In Situ [DCIS]).

**Supplementary Fig. S3**. Cluster assignments generated by SCANPY, SEDR, CCST, STAGATE, DeepST, GraphST, and STMSGAL on Adult Mouse Brain (FFPE).

**Supplementary Fig. S4**. Visualizations of spatial domains and expressions of the corresponding marker genes identified by STMSGAL with Louvain clustering on adult mouse hippocampus tissue.

## Abbreviations

ARI: adjusted Rand index; CH: Calinski–Harabasz; DB: Davies–Bouldin; DCIS: ductal carcinoma in situ; DCIS/LCIS: ductal carcinoma in situ/lobular carcinoma in situ; ELU: exponential linear unit; GATE: graph attention autoencoder; HDST: high-definition spatial transcriptomics; IDC: invasive ductal carcinoma; NGS: next-generation sequencing; ReLu: rectified linear units; scRNA-seq: single-cell RNA sequencing; SNN: spatial neighbor network; ST: spatial transcriptomics.
