# Supplementary material for: Unveiling patterns in spatial transcriptomics data: a novel approach utilizing graph attention autoencoder and multiscale deep subspace clustering network
Source: Gigascience. 2025 Jan 13;14:giae103. doi: 10.1093/gigascience/giae103 (PMC11727722; doi:10.1093/gigascience/giae103)
Supplement: giae103_GIGA-D-24-00044_Revision_3 [file giae103_giga-d-24-00044_revision_3.pdf]

GigaScience

Unveiling Patterns in Spatial Transcriptomics Data: A Novel Approach Utilizing Graph Attention Autoencoder and Multi-Scale Deep Subspace Clustering Network

--Manuscript Draft--

**Manuscript Number:**	GIGA-D-24-00044R3
**Full Title:**	Unveiling Patterns in Spatial Transcriptomics Data: A Novel Approach Utilizing Graph Attention Autoencoder and Multi-Scale Deep Subspace Clustering Network
**Article Type:**	Technical Note

Funding Information:

National Natural Science Foundation of China (62072172)	Dr Liqian Zhou
National Natural Science Foundation of China (62172158)	Dr Min Chen
Natural Science Foundation of Hunan Province (2021JJ30219)	Dr Liqian Zhou
National Natural Science Foundation of China (61803151)	Dr Lihong Peng
Natural Science Foundation of Hunan Province (2023JJ50201)	Dr Lihong Peng

Abstract:

Background: The accurate deciphering of spatial domains, along with the identification of differentially expressed genes and the inference of cellular trajectory based on spatial transcriptomic (ST) data, holds significant potential for enhancing our understanding of tissue organization and biological functions. However, most of spatial clustering methods can neither decipher complex structures in ST data nor entirely employ features embedded in different layers.

Results: This manuscript introduces STMSGAL, a novel framework for analyzing ST data by incorporating graph attention autoencoder and multi-scale deep subspace clustering. Firstly, STMSGAL constructs ctaSNN, a cell type-aware shared nearest neighbor graph, using Louvian clustering exclusively based on gene expression profiles. Subsequently, it integrates expression profiles and ctaSNN to generate spot latent representations using a graph attention auto-encoder and multi-scale deep subspace clustering. Lastly, STMSGAL implements spatial clustering, differential expression analysis, and trajectory inference, providing comprehensive capabilities for thorough data exploration and interpretation. STMSGAL was evaluated against seven methods including SCANPY, SEDR, CCST, DeepST, GraphST, STAGATE, and SiGra, using four 10x Genomics Visium datasets, one mouse visual cortex STARmap dataset, and two Stereo-seq mouse embryo datasets. The comparison showcased STMSGAL's remarkable performance across Davies-Bouldin, Calinski-Harabasz, S_Dbw, and ARI values. STMSGAL significantly enhanced the identification of layer structures across ST data with different spatial resolutions, and accurately delineated spatial domains in two breast cancer tissues, adult mouse brain (FFPE), and mouse embryos.

Conclusion: STMSGAL can serve as an essential tool for bridging the analysis of cellular spatial organization and disease pathology, offering valuable insights for researchers in the field.

**Corresponding Author:**	Lihong Peng<br>Hunan University of Technology<br>Zhuzhou, CHINA
**Corresponding Author Secondary Information:**	
**Corresponding Author's Institution:**	Hunan University of Technology
**Corresponding Author's Secondary Institution:**	

Powered by Editorial Manager® and ProduXion Manager® from Aries Systems Corporation

First Author:	Lihong Peng
First Author Secondary Information:	
Order of Authors:	Lihong Peng
	Liqian Zhou
	Xinhuai Peng
	Min Chen
	Xianzhi He
	Geng Tian
	Jialiang Yang
Order of Authors Secondary Information:	
Response to Reviewers:	We are very grateful to the chief editor and reviewers for patient, meticulous, and professional review work with the aim of improving the quality of our manuscript. The font in the modified area is highlighted in blue. Significantly, Liqian Zhou is the first author, Xinhuai Peng and Liqian Zhou have contributed equally to this work and share first authorship.<br>1) Please include a citation to your new GigaDB dataset (including the DOI link) to your reference list, and cite this in the data availability section.<br>Response: Thank you very much! We have updated it.<br><br>2) Likewise, please also add other data sources to the bibliography as individual numbered items. Please refer to our citation guidelines on the journal homepage for details on the correct format for URL/doi citations. Please cite these datasets from the "data availability" section and also from the section "datasets" (page 3).<br>Response: Thank you very much! We have cite these datasets from the "data availability" section and also from the section "datasets".<br><br>3) In the data availability section, please add a line regarding DOME-ML annotations (e.g. "DOME-ML (Data, Optimization, Model and Evaluation in Machine Learning) annotations are available via a link in GigaDB[xx]."<br>Response: Thank a lot! We have add a line regarding DOME-ML annotations.<br><br>4) Please add ORCIDs for all authors to the title page, where available. We have the following on our files: ORCID iDs: Lihong Peng [0000-0002-2321-3901]; Liqian Zhou; Xinhuai Peng; Min Chen; Xianzhi He; Geng Tian [0000-0001-5752-4436]; Jialiang Yang [0000-0003-4689-8672].<br>Response: Thank you very much! We have updated it.<br><br>5) Minor point: You thank "anonymous" referees in the acknowledgements - please note that GigaScience has named, non-anonymous peer review, you may delete the word "anonymous".<br>Response: Thank you very much! We have deleted it.<br>6) You cite several preprints (e.g. nr 45, 46, 60, 70, 77, 78). Please check if there is meanwhile a published journal version for any of these preprints, and if there is, please cite the journal version instead. (If there is only a preprint version citing the preprint is totally fine though).<br>Response: Thank you very much! We have updated it.<br><br>7) Please upload editable source files for the text to EM - LaTex or WORD, whatever you used. (currently we only have a PDF).<br>Response: Thank you very much! We have uploaded them.<br><br>8) Please also ensure that your revised manuscript conforms to the journal style, which can be found in the Instructions for Authors on the journal homepage.<br>Response: Thank you very much! We have revised manuscript.
Additional Information:	
Question	Response

Are you submitting this manuscript to a special series or article collection?	No
**Experimental design and statistics**<br><br>Full details of the experimental design and statistical methods used should be given in the Methods section, as detailed in our Minimum Standards Reporting Checklist. Information essential to interpreting the data presented should be made available in the figure legends.<br><br>Have you included all the information requested in your manuscript?	Yes
**Resources**<br><br>A description of all resources used, including antibodies, cell lines, animals and software tools, with enough information to allow them to be uniquely identified, should be included in the Methods section. Authors are strongly encouraged to cite Research Resource Identifiers (RRIDs) for antibodies, model organisms and tools, where possible.<br><br>Have you included the information requested as detailed in our Minimum Standards Reporting Checklist?	Yes
**Availability of data and materials**<br><br>All datasets and code on which the conclusions of the paper rely must be either included in your submission or deposited in publicly available repositories (where available and ethically appropriate), referencing such data using a unique identifier in the references and in the "Availability of Data and Materials" section of your manuscript.<br><br>Have you have met the above requirement as detailed in our Minimum	Yes

Standards Reporting Checklist?	

Powered by Editorial Manager® and ProduXion Manager® from Aries Systems Corporation

Manuscript

Click here to access/download;Manuscript;STMSGAL-GigaScience-11-5.pdf

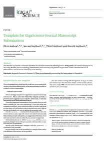

GigaScience, 2024, 1–22

doi: xx.xxxx/xxxx
Manuscript in Preparation
Paper

PAPER

Unveiling Patterns in Spatial Transcriptomics Data: A Novel Approach Utilizing Graph Attention Autoencoder and Multi-Scale Deep Subspace Clustering Network

Liqian Zhou[1,†], Xinhuai Peng[1,†], Min Chen[2,†], Xianzhi He[1], Geng Tian[3], Jialiang Yang[3,*] and Lihong Peng[1,4,*]

[1]School of Computer Science, Hunan University of Technology, Zhuzhou, 412007, Hunan, China and [2]School of Computer Science, Hunan Institute of Technology, Hengyang, 421002, Hunan, China and [3]Geneis (Beijing) Co. Ltd., Beijing, 100102, China and [4]College of Life Science and Chemistry, Hunan University of Technology, Zhuzhou, 412007, Hunan, China

* Correspondence: one email address for each corresponding authorCorresponding author. yangjl@geneis.cn (Jialiang Yang); plhhnu@163.com (Lihong Peng)
[†]These authors have contributed equally to this work and share first authorship.

Abstract

Background: The accurate deciphering of spatial domains, along with the identification of differentially expressed genes and the inference of cellular trajectory based on spatial transcriptomic (ST) data, holds significant potential for enhancing our understanding of tissue organization and biological functions. However, most of spatial clustering methods can neither decipher complex structures in ST data nor entirely employ features embedded in different layers.
Results: This manuscript introduces STMSGAL, a novel framework for analyzing ST data by incorporating graph attention autoencoder and multi-scale deep subspace clustering. Firstly, STMSGAL constructs ctaSNN, a cell type-aware shared nearest neighbor graph, using Louvian clustering exclusively based on gene expression profiles. Subsequently, it integrates expression profiles and ctaSNN to generate spot latent representations using a graph attention auto-encoder and multi-scale deep subspace clustering. Lastly, STMSGAL implements spatial clustering, differential expression analysis, and trajectory inference, providing comprehensive capabilities for thorough data exploration and interpretation. STMSGAL was evaluated against seven methods including SCANPY, SEDR, CCST, DeepST, GraphST, STAGATE, and SiGra, using four 10x Genomics Visium datasets, one mouse visual cortex STARmap dataset, and two Stereo-seq mouse embryo datasets. The comparison showcased STMSGAL's remarkable performance across Davies-Bouldin, Calinski-Harabasz, S_Dbw, and ARI values. STMSGAL significantly enhanced the identification of layer structures across ST data with different spatial resolutions, and accurately delineated spatial domains in two breast cancer tissues, adult mouse brain (FFPE), and mouse embryos.
Conclusion: STMSGAL can serve as an essential tool for bridging the analysis of cellular spatial organization and disease pathology, offering valuable insights for researchers in the field.

Key words: spatial transcriptomics; graph attention autoencoder; deep subspace clustering; multi-scale self-expression; self-supervised learning; latent embedding feature learning; cell type-aware spatial neighbor network; differential expression analysis; trajectory inference.

Background

The tissues in human body comprise various cell types where each cell type implements a particular function [1]. The activation of a

Compiled on: November 6, 2024.
Draft manuscript prepared by the author.

Key Points

- A graph attention autoencoder is fully utilized to effectively integrate spatial locations and gene expression information by collectively incorporating information between neighboring spots.
- A multi-scale self-expression module is explored to learn the associations between node representations in all encoder layers and further obtain a more distinct self-expression coefficient matrix for mapping these features into a more precise subspace.
- A self-supervised learning method is designed to help spot latent feature learning by utilizing the clustering label as a supervisor.

cell is mainly affected by its surrounding environment [2, 3, 4, 5]. Exploring relative positions of these cells contributes to analyzing cell-cell communication [6, 7, 8, 9] and their spatial organization and disease pathology [10, 11, 12, 13]. The rapid advance of single-cell RNA sequencing (scRNA-seq) technologies enables us to investigate the gene expression patterns of various cells within a tissue/organ [14, 15, 16, 17, 18, 19, 20, 21, 22]. However, scRNA-seq technologies fail to provide spatial location information [23]. In contrast, spatial transcriptomics (ST) technologies provide a large number of gene expression data and cellular location information for a tissue and have witnessed tremendous development in the past several years [24, 25, 26]. Based on the data generation ways, ST technologies mainly contain image-based methods and next-generation sequencing-based (NGS-based) methods [27].

Image-based methods use *in situ* sequencing or *in situ* hybridization to retain spatial locations of cells and further obtain RNA transcripts based on images from the stained tissues. MERFISH [28] can detect gene expression information of about 40,000 human cells in a single 18-hour measurement. STARmap [29] can capture more than 1,000 genes in the mouse cortex through an error-robust sequencing-by-ligation approach. seqFISH+ [30] combined sequential hybridization and standard confocal microscope-based imaging technique to obtain super-resolution imaging and multiplexing data for 10,000 genes.

NGS-based methods depend on the number of spatial barcodes before library preparation [31]. Slide-seq [32, 33] obtained randomly barcoded positions through *in situ* indexing and captured mRNAs through depositing onto a slide. High-definition ST (HDST) [34] replaced the glass slides using beads deposited in wells. The DBiT-seq [35] technique utilized polyT barcodes in the tissue section based on microfluidics. Stereo-seq [36] obtained nanoscale resolution through randomly barcoded DNA nanoballs. 10x Genomics Visium [37] demonstrated increased resolution with 55μm in diameter and 100μm center-center and sensitivity more than 10,000 transcripts per spot. It detected more unique molecules for each spot compared with Slide-seq and HDST.

One main challenge in ST data analysis is to capture spatial domains with similar expression patterns. For example, the laminar organization in human cerebral cortex has close relationship with its biological functions. In this tissue, cells within different cortical layers have different expressions, morphology and physiology [38]. One efficient way to identify spatial domains is to cluster ST data. These clustering methods are mainly fall into two categories. The first category adopts conventional clustering methods, for example, K-means clustering [39] and Louvain algorithms [40]. These algorithms are susceptible to small size of spots and sparsity data, and the detected clusters may be discontinuous in sections. The other category uses cell-type labels obtained from scRNA-seq data to deconvolute spots [41, 42]. But this type of methods can not analyze ST data from the perspective of the cellular or subcellular resolution.

It is crucial to learn a discriminative representation for each spot by combining gene expression and spatial contexts when clustering ST data. Recently, several clustering algorithms have been developed to identify spatial domains. For example, BayesSpace [43] assumed that spots belonging to the same cell type may be closer each other and built a Markov random field model with Bayesian approach. stLearn [44] first proposed a spatial morphological gene expression normalization algorithm to normalize ST data, and then employed standard Louvain clustering approach to partition broad clusters into several sub-clusters. SEDR [45] exploited a deep autoencoder network to learn gene representations and adopted a variational graph autoencoder to embed spatial information. CCST [46] explored a graph convolutional network to transfer gene expression information as cellular embedding vectors, and trained a neural network to encode cell embedding features for clustering. STAGATE [47] developed a adaptive graph attention autoencoder (GATE) [48] to accurately identify spatial domains by integrating gene expression information and spatial neighbor network. DeepST [49] incorporated gene expression, spatial context, and histology to model spatially embedded representation and further capture spatial domains. GraphST [50] integrated graph self-supervised contrastive learning and graph neural network [51, 52] for spatial clustering, multi-sample integration, and cell-type deconvolution. ConGI [53] adopted gene expression with histopathological images to accurately capture spatial domains based on contrastive learning. STGIC [54] is a graph and image-based spatial clustering method. It can generate pseudo-labels for spatial clustering while does not depend on any trainable parameters. SPACEL [55] deconvoluted cell type composition based on a multiple-layer perceptron, and identified spatial domains via graph convolutional network and adversarial learning, lastly constructed a 3D architecture for each tissue. PRECAST [56] integrated a few ST datasets that have complex batch effects and biological effects. SRTsim [57] is spatially resolved transcriptomics-specific simulator for spatial clustering and expression pattern analysis. Tang et al. [58] developed an image-augmented graph transformer for spatial elucidation. The methods mentioned above have significantly promoted the studies of tissue physiology from cell-centroid to structure-centroid and are state-of-the-art spatial clustering methods. Particularly, Yuan et al. [59] considered that current computation-based ST clustering is lack of a comprehensive benchmark and have systematically benchmarked a collection of 13 spatial clustering methods on 7 ST datasets (34 ST data). Their work has provided guidance for future progresses in the ST data analysis field.

Although the aforementioned clustering methods obtained impressive performance, their learned latent node representation failed to achieve the most useful information because they did not use current clustering labels. In addition, some methods including SEDR and CCST only used the representation in the final hidden layer of an encoder for clustering ST data, which failed to consider helpful features in the other layers. Although graph attention autoencoder-based methods [60, 61] have elucidated better performance in integrating node attributes and graph structure information, they can not decipher the complex structures in ST data or did not entirely employ features embedded in different layers. Moreover, some models did not utilize a clustering-oriented loss function while others did not fully use the clustering labels for node representation learning. The problems produced the suboptimal clustering results. Here, we introduce STMSGAL, an ST anal-

ysis framework by combining graph attention autoencoder and multi-scale deep subspace clustering network.

Materials and methods

Overview of STMSGAL

As shown in Figure 1, STMSGAL is composed of three main steps. (i) Spatial neighbor network construction. STMSGAL constructs a spatial neighbor network (SNN) based on spatial contexts, and obtains a cell type-aware SNN called ctaSNN through Louvain clustering exclusively based on gene expression data. (ii) Latent embedding feature learning. It mainly comprises spot embedding feature matrix construction, subspace clustering combining multi-scale self-expression coefficient learning and affinity matrix construction, and spot robust latent feature learning based on self-supervised learning. (iii) Biological applications. ST data are clustered and differential expression analysis and trajectory inference are implemented. Similar to STAGATE [47], STMSGAL still constructs a ctaSNN and embedding feature matrix using GATE. However, differed from STAGATE, STMSGAL adopt the multi-scale deep subspace clustering algorithm to obtain cluster labels based on multi-scale information from each encoder layer for spots, and then adopt a self-supervised module to learn robust latent features of spots with clustering information.

Datasets

Four available 10x Genomics Visium datasets, one mouse visual cortex STARmap dataset, and two Stereo-Seq datasets are used to evaluate the STMSGAL performance. The former four 10x Genomics datasets are from Adult Mouse Brain (FFPE) [62], Human Breast Cancer (Block A Section 1) [63], Human Breast Cancer (Ductal Carcinoma In Situ (DCIS)) [64], and Human dorsolateral prefrontal cortex (DLPFC) tissues [65]. The former two datasets have no clustering labels and the latter two datasets are known to be labeled. The Adult Mouse Brain (FFPE) dataset contains 2,264 spots and 19,465 genes. Human Breast Cancer (DCIS) dataset includes 3,798 spots and 36,601 genes. Human Breast Cancer (Block A Section 1) dataset detects 2,518 spots and 19,743 genes. The DLPFC dataset contains 12 tissue slices. It captures 33,538 genes with different spot numbers ranged from 3,460 to 4,789 in each slice. Each slice contains 5 to 7 regions by manually annotation [38]. The mouse visual cortex STARmap dataset provides the expression information of 1020 genes from 1207 cells [29]. The Stereo-Seq dataset [66] from mouse embryos at E9.5 is obtained based on high-resolution full-transcriptome coverage technologies (i.e., Stereo-Seq technology). The number of spots and one of genes are 5,913 and 25,568 (E9.5_E1S1) as well as 4,356 and 24,107 (E9.5_E2S2), respectively.

Spatial neighbor network construction

Data preprocessing

To preprocess ST data, first, spots outside main tissue regions are removed. Next, raw gene expressions are log-transformed and normalized based on library size through the SCANPY package [67]. Finally, multiple highly variable genes are selected as inputs.

Cell type-aware SNN construction

To integrate the similarity between spots neighbor to a given spot, similar to STAGATE [47], STMSGAL constructs an undirected neighbor network based on a pre-defined radius r and spatial contexts. Let $\boldsymbol{A}$ denote an adjacency matrix of the constructed SNN, $A_{ij} = 1$ when the Euclidean distance between two spots i and j is less than r. For 10x Genomics Visium data, an SNN where each spot contains six nearest neighbors is built. Next, self-loops are added to each spot. Finally, the SNN is pruned based on pre-clustering and a ctaSNN is constructed. Particularly, the pre-clustering of spots is conducted by Louvain clustering [40] exclusively based on gene expression profiles. The edges where two spots linking them belong to different clusters are pruned.

Latent embedding feature learning

Wang et al. [68] presented a multi-scale graph attention subspace clustering model and obtained superior performance on three graph datasets and two real-world datasets. The clustering model fully explored the associations between node representations in all encoder layers and obtained more accurate self-expression coefficient matrix. To more accurately cluster spots, in this section, we utilize the multi-scale graph attention subspace clustering model [68] to learn latent embedding features of spots. First, spot embedding feature matrix in each encoder layer is constructed via GATE. Second, spot cluster labels are obtained through subspace clustering. Finally, spot robust latent features are learned by self-supervised learning.

Embedding feature matrix construction

Similar to STAGATE [47], we use GATE to construct embedding feature matrix. For spot i, an encoder with L layers takes its normalized gene expressions $\boldsymbol{x}_i$ as inputs to generate its embedding features by collectively incorporating information of its neighbors. Taking gene expressions as initial spot embeddings, that is, $\boldsymbol{h}_i^{(0)} = \boldsymbol{x}_i, \forall i \in \{1, 2, \cdots, N\}$, the embedding of i in the k-th ($k \in \{1, 2, \cdots, L-1\}$) encoder layer is denoted by Eq. (1):

$$\boldsymbol{h}_i^{(k)} = \sigma\left(\sum_{j \in S_i} \boldsymbol{att}_{ij}^{(k)} \left(\boldsymbol{W}_k \boldsymbol{h}_j^{(k-1)}\right)\right) \tag{1}$$

where $\boldsymbol{W}_k$, σ, S_i, and $\boldsymbol{att}_{ij}^{(k)}$ denote the trainable weight matrix, nonlinear activation function, a spot set that includes neighbors of i in SNN and i itself, and weight of the edge between spot i and spot j in the k-th graph attention layer, respectively. The output $\boldsymbol{z}_i^{(k)} = \boldsymbol{h}_i^{(k)}$ of the encoder is taken as the final spot embedding in the encoder part. The L-th layer in the encoder does not use the attention layer by Eq. (2):

$$\boldsymbol{h}_i^{(L)} = \sigma\left(\boldsymbol{W}_L \boldsymbol{h}_i^{(L-1)}\right) \tag{2}$$

In the part of decoder, a decoder transforms the learned latent embedding back into a normalized expression profile to reconstruct the spot features. Suppose that $\widehat{\boldsymbol{h}}_i^{(L)} = \boldsymbol{C}\boldsymbol{z}_i^{(L)}$ where $\boldsymbol{C}$ denote a self-expression matrix, and $\boldsymbol{z}_i^{(L)}$ denotes the embedding of i in the L-th encoder layer. Next, $\boldsymbol{C}\boldsymbol{z}_i^{(L)}$ is feed into the decoder to reconstruct the spot embeddings. In the k-th decoder layer, the embedding features of spot i is constructed by Eq. (3):

$$\widehat{\boldsymbol{h}}_i^{(k-1)} = \sigma\left(\sum_{j \in S_i} \widehat{\boldsymbol{att}}_{ij}^{(k-1)} \left(\widehat{\boldsymbol{W}}_k \widehat{\boldsymbol{h}}_j^{(k)}\right)\right) \tag{3}$$

The L layer in the decoder is denoted by Eq. (4):

$$\widehat{\boldsymbol{h}}_i^{(0)} = \sigma\left(\widehat{\boldsymbol{W}}_1 \widehat{\boldsymbol{h}}_i^{(1)}\right) \tag{4}$$

Its output is the reconstructed normalized expressions. In addition, we set $\widehat{\boldsymbol{W}}_k = \boldsymbol{W}_k{}^T$ and $\widehat{\boldsymbol{att}}^{(k)} = \boldsymbol{att}^{(k)}$ to avoid overfitting.

The attention mechanism is a one-layer feedforward neural

network that is parametrized by a weight vector. A self-attention mechanism [69] is used to compute the similarity between neighboring spots by an adaptive way. In the k-th decoder layer, the edge weight between spot i and its neighbor spot j is computed by Eq. (5):

$$e_{ij}^{(k)} = \text{Sigmoid}(\boldsymbol{v}_s^{(k)^T}(\boldsymbol{W}_k \boldsymbol{h}_i^{(k-1)}) + \boldsymbol{v}_r^{(k)^T}(\boldsymbol{W}_k \boldsymbol{h}_j^{(k-1)})) \tag{5}$$

where $\boldsymbol{v}_s^{(k)}$ and $\boldsymbol{v}_r^{(k)}$ are two trainable weight vectors. Next, the similarity weights between spots are normalized by a softmax function by Eq. (6):

$$att_{ij}^{(k)} = \frac{\exp\left(e_{ij}^{(k)}\right)}{\sum_{j \in S_i} \exp\left(e_{ij}^{(k)}\right)} \tag{6}$$

The obtained weights are applied to further update the latent embedding of spots in the encoder and decoder.

In addition, STMSGAL adopts a self-attention mechanism and constructs a ctaSNN. Let $\boldsymbol{att}_{ij}^{spatial}$ and $\boldsymbol{att}_{ij}^{aware}$ denote the learned spot similarity using SNN and ctaSNN, respectively, the final spatial similarity is computed by combining the above two similarities by Eq. (7):

$$\boldsymbol{att}_{ij} = (1 - \alpha)\, \boldsymbol{att}_{ij}^{\text{spatial}} + \alpha \boldsymbol{att}_{ij}^{\text{aware}} \tag{7}$$

where α is a hyperparameter used to weigh the importance of SNN and ctaSNN.

The reconstructed loss is minimized based on the residual sum of squares by Eq. (8):

$$\mathcal{L}_{att} = \min \frac{1}{2} \sum_{i=1}^{n} \left\| \boldsymbol{x}_i - \hat{\boldsymbol{h}}_i^{(0)} \right\|_F^2 \tag{8}$$

Particularly, weight decay equally imposes a penalty to the L_2 norm, thus, the regularized loss is minimized. And the total loss is represented as Eq. (9):

$$\mathcal{L}_1 = \mathcal{L}_{att} + \frac{1}{2} \cdot \sum_{k=1}^{L-1} \|\boldsymbol{W}_k\|_F^2 \tag{9}$$

Multi-scale deep subspace clustering

Differed from STAGATE [47], in this section, we adopt the multi-scale deep subspace clustering algorithm to obtain cluster labels based on multi-scale information from each encoder layer for spots. The self-expression property of data greatly influences the performance of subspace clustering. In a union subspace, each data can be represented as a linear combination of the other data. Thus, we use a multi-scale self-expressive module to obtain the final self-expression coefficient matrix based on spot embedding feature matrix: $\boldsymbol{H}^{(k)} = \left\{\boldsymbol{h}_1^{(k)}, \boldsymbol{h}_2^{(k)}, \cdots, \boldsymbol{h}_n^{(k)}\right\}$.

In deep subspace clustering network [70], a self-expression layer is a full connection layer without bias and activation. And its objection function is represented by Eq. (10):

$$\min_{\boldsymbol{C}} \|\boldsymbol{C}\|_p + \frac{1}{2}\|\boldsymbol{Z} - \boldsymbol{CZ}\|_F^2 \quad s.t. \quad (\text{diag}(\boldsymbol{C}) = 0) \tag{10}$$

where $\boldsymbol{C}$ indicates a self-expression coefficient matrix used to build an affinity matrix $\boldsymbol{\Lambda}$ for the following spectral clustering, $\boldsymbol{Z}$ indicates the output feature matrix in the encoder, and $\|\cdot\|_p$ indicates an arbitrary regularization norm.

Although deep subspace clustering obtains better clustering performance, it fails to consider the multi-scale features existing in the other encoder layers. Here, we integrate the multi-scale features to the original self-expression module. Given the input normalized gene expressions in the i-th encoder layer $\boldsymbol{Z}^{(k)}$ $(k = 1, 2, \cdots, L)$, the self-expression coefficient matrix $\boldsymbol{C}^{(k)}$ in the k-th encoder layer can be computed by Eq. (11):

$$\min_{\boldsymbol{C}^{(k)}} \frac{1}{2} \left\| \boldsymbol{Z}^{(k)} - \boldsymbol{C}^{(k)} \boldsymbol{Z}^{(k)} \right\|_F^2 \tag{11}$$

Next, the multi-scale self-expression matrix $\boldsymbol{C}^{(k)}$ in different layers is fused based on an adaptive approach by Eq. (12):

$$\boldsymbol{C}_F = \frac{\sum_{k=1}^{L} \tau_k \cdot \boldsymbol{C}^{(k)}}{\sum_{k=1}^{L} \tau_k} \tag{12}$$

where τ_k denotes a trainable variable used to balance the importance of each self-expression matrix.

Based on the obtained final self-expression matrix $\boldsymbol{C}_F$, deep subspace clustering model builds an affinity matrix $\boldsymbol{\Lambda}$ for spectral clustering [71] by Eq. (13):

$$\boldsymbol{\Lambda} = \frac{1}{2}\left(\left|\boldsymbol{C}_F\right| + \left|\boldsymbol{C}_F^{\mathrm{T}}\right|\right) \tag{13}$$

Consequently, the clustering result $\boldsymbol{Y}_{clu}$ can be obtained by spectral clustering based on $\boldsymbol{\Lambda}$.

Particularly, the multi-scale self-expression loss is represented as Eq. (14):

$$\begin{aligned} \mathcal{L}_{mss} &= \min_{\boldsymbol{C}^{(k)}} \frac{1}{2L} \cdot \sum_{k=1}^{L} \left\| \boldsymbol{Z}^{(k)} - \boldsymbol{C}^{(k)} \boldsymbol{Z}^{(k)} \right\|_F^2 \\ \text{s.t.} & \quad \left(\text{diag}\left(\boldsymbol{C}^{(k)}\right) = 0\right) \end{aligned} \tag{14}$$

Besides, a regularization loss is introduced to avoid $\boldsymbol{C}^{(k)}$ too sparse:

$$\begin{aligned} \mathcal{L}_{\text{reg}} &= \min_{\boldsymbol{C}^{(k)}} \frac{1}{L} \cdot \sum_{k=1}^{L} \left\| \boldsymbol{C}^{(k)} \right\|_p \\ \text{s.t.} & \quad \left(\text{diag}\left(\boldsymbol{C}^{(k)}\right) = 0\right) \end{aligned} \tag{15}$$

Thus, the total loss in multi-scale self-expression module is denoted as Eq. (16):

$$\mathcal{L}_2 = \mathcal{L}_{mss} + \mathcal{L}_{reg} \tag{16}$$

Spot robust latent feature learning

Furthermore, distinct from [47], we employ a self-supervised module to learn spot robust latent features. First, spots are classified based on three full connection layers. Let the dimensions of all full connection layers be denoted as $\{d_L \times D_1 \times D_2 \times D_3 \times m\}$, where d_L denotes the dimension of $\boldsymbol{Z}^{(L)}$, and D_1, D_2 and D_3 denote the dimensions of three full connection layers, respectively. We obtain the classification results $\boldsymbol{P} \in R^{n \times m}$ of n spots based on the three full connection layers.

Next, we use the cross-entropy loss between the classification results $\boldsymbol{P}$ and the clustering results $\boldsymbol{Y}_{clu}$ to constrain self-supervised learning module by Eq. (17):

$$\mathcal{L}_3 = \mathcal{L}_{sup} = \min_{\boldsymbol{P}} - \sum_{i=1}^{n} \sum_{j=1}^{m} \boldsymbol{P}(i,j) \log \boldsymbol{Y}_{clu}(i,j) \tag{17}$$

where $\boldsymbol{Y}_{clu}(i,j)$ denotes the j-th clustering label of spot i obtained from spectral clustering, and $\boldsymbol{P}(i,j)$ denotes the j-th classification label of spot i based on three full connection layers.

Finally, by integrating Eqs. (9), (14), (15), and (17), the total

loss function of multi-scale GATE is denoted as Eq. (18):

$$\mathcal{L}_{total} = \min_{(C,P,Z)} \mathcal{L}_1 + \mathcal{L}_{reg} + \lambda \cdot \mathcal{L}_{mss} + \mathcal{L}_{sup} \quad (18)$$

where λ is a tradeoff parameter used to measure the importance of $\mathcal{L}_{mss}$.

Biological application

STMSGAL first identifies spatial domains using Leiden clustering [72], Louvain clustering [40] or mclust clustering [73] based on the obtained spot embedding feature matrix. Second, it implements differential expression analysis using the t-test in the Scanpy package. Finally, it conducts trajectory inference.

Spatial clustering

Based on the learned spot embedding feature matrix, we use different strategies to identify spatial domains. For the DLPFC dataset, mclust clustering [73] is applied to spatial clustering. For other datesets, Louvain or Leiden clustering [40, 72] is used to implement ST clustering.

In addition, although spot embedding feature matrix is obtained by integrating both gene expressions and spatial contexts, several spots may be incorrectly assigned to spatially diametrical domains, which may cause noise and influence downstream analysis. To solve this problem, an optional optimization step is used to further optimize spatial clustering results obtained from Louvain clustering on the DLPFC dataset: for a given spot i, its surrounding spots within an r radius circle are taken as its neighbors. Next, we reassign i to a spatial domain with the most frequent label of its neighbors. In addition, the clustering results are visualized using UMAP [74].

Differential expression analysis

Differential expression analysis is one primary downstream analysis method on transcriptomic data [75, 76, 77]. It helps identify biomarkers for novel cell types or detect gene signatures for cellular heterogeneity, and further provides data for other secondary analyses (such as gene set or pathway analysis, and network analysis). We use the t-test implemented in the SCANPY package [67] to identify differentially expressed genes for spatial domains.

Trajectory inference

ST technologies help depict tissues and organisms in great detail. Tracking the transcriptomic profiles of cells over time and studying their dynamic cellular process contributes to the computational reconstruction of cellular developmental processes. Trajectory inference enables us to better study the potential dynamics of a query biological process, for example, cellular development, differentiation, and immune responses [78]. It can detect a graph-like structure existing in the dynamic process from the sampled cells. Properties of cells are compared over pseudotime [79] by mapping them to the captured structure. Trajectory inference allows us analyze how cells evolve from one cell state to another, and when and how should cells make cell fate decisions. In this section, the PAGA algorithm [80] in the SCANPY package [67] is employed to depict spatial trajectory. The obtained trajectory figures are visualized using the *scanpy.pl.paga_compare*() function.

Results

Experimental setting

In STMSGAL, both encoder and decoder with the activation function of exponential linear unit (ELU) [81] were neural networks with two graph attention layers, where the number of neurons is 512 and 30, respectively. The Adam optimizer [82] was employed to minimize their reconstruction loss. In the self-supervised module, the activation function was set to rectified linear units (ReLu) [83]. For Louvain clustering, the radius r was set to 50 when STMSGAL obtained the best clustering performance on the DLPFC dataset.

STMSGAL adopted the same data preprocessing as those of SCANPY. Both of them used log-normalized, constructed the nearest neighbor network. SCANPY obtained spatial clustering with the *scanpy.tl.louvain*() function. Table 1 shows parameter settings of STMSGAL on five ST datasets. For each dataset with labels, the resolution parameter was tuned manually to ensure the cluster number was equal to the ground truth. Thus, the cluster number in each method was set to the same as one of ground truth layers. For other clustering methods, we adopted their default settings.

Evaluation metrics

For three datasets with labels (Human Breast Cancer (Block A Section 1), DLPFC, and mouse visual cortex STARmap), we employed adjusted rand index (ARI) [84] to evaluate the performance of different spatial clustering algorithms. ARI computes the similarity between the predicted clustering labels and reference cluster labels by Eq. (19):

$$ARI = \frac{RI - E[RI]}{\max(RI) - E[RI]} \quad (19)$$

where the unadjusted rand index $RI = (a + b)/C_n^2$ where a and b indicate the number of pairs correctly labeled in the same dataset and not in the same dataset, respectively. C_n^2 indicates the total number of possible pairs. $E[RI]$ indicates the expected RI based on random labeling. A higher ARI score denotes better performance.

For two datasets whose spatial domain annotations are unavailable (Adult Mouse Brain (FFPE) and Human Breast Cancer (DCIS)), we evaluated the performance of spatial clustering algorithms based on three clustering metrics, that is, Davies-Bouldin (DB) score [85], Calinski-Harabasz (CH) score [86], and S_Dbw score [87, 88]. DB was computed by averaging all cluster similarities where the similarity between each cluster and its most similar cluster was taken as its cluster similarity. And the similarity was computed by the ratio of within-cluster distances to between-cluster distances. CH is used to measure the cluster validity by averaging the squares of within- and between-cluster distance sum of all spots. S_Dbw evaluate intraclass compactness and interclass density of each spot. Small DB and S_Dbw and large CH indicate the optimal cluster clustering.

Performance comparison of STMSGAL with six other methods on two datasets without labels

To investigate the clustering performance of STMSGAL, we compared it with five other clustering algorithms, that is, SCANPY [67], SEDR [45], CCST [46], STAGATE [47], DeepST [49], and GraphST [50] on two 10x Genomics Visium datasets without labels (i.e., Adult Mouse Brain (FFPE) and Human Breast Cancer (DCIS)). The former one method obtained broad applications in single-cell clustering, and the remaining five methods were widely applied to spatial clustering. Table 2 shows the DB, CH, and S_Dbw scores computed by STMSGAL and other methods on the above two datasets. The best performance in each column was denoted using the bold font. The results demonstrated that STMSGAL computed the smallest DB and S_Dbw and the highest CH on Adult Mouse Brain (FFPE), and the highest CH and the smallest S_Dbw on Human Breast Cancer (DCIS), suggesting its optimal clustering performance.

STMSGAL demonstrates robust clustering performance across ST datasets with different spatial resolutions

To evaluate the STMSGAL performance on spatial domain identification, we compared it with existing seven state-of-the-art methods on 4 DLPFC sections. Particularly, in complex networks, nodes are clustered into relatively dense communities through the clustering algorithm. Louvain clustering is a nonspatial clustering algorithm. It assigns each spot to significantly differential community and achieves the desired clusters by iteratively merging and splitting communities. It exhibits powerful clustering performance than spectral clustering when clustering ST data, such as DLPFC. Thus, we used the Louvian clustering for performing clustering again on DLPFC.

Moreover, the DLPFC dataset provides high resolution images and satisfies the need of spatial clustering methods including SiGra that must combine high resolution images for clustering ST data. The results elucidated that spatial domains captured by STMSGAL were consistent with manual annotation on human DLPFC sections and the definition of cortical stratification in neuroscience (Figure 2).

In addition, STMSGAL effectively captured the expected cortical layer structures and significantly improved spatial clustering performance in comparison with SCANPY, SEDR, CCST, STAGATE, SiGra, DeepST, and GraphST (Figure 2 and Supplementary Figure S1). For average ARIs, STMSGAL achieved the best performance (Figure 2B). In the DLPFC section 151509, STMSGAL clearly depicted the layer borders and obtained the best average ARI of 0.511. In the section, although the clustering results of SCANPY roughly adhered to the expected layer structures, its cluster boundary was discontinuous with many noises, which greatly influenced its clustering accuracy. Moreover, SCANPY is a non-spatial clustering algorithm, and SEDR, CCST, DeepST, STAGATE, SiGra, and GraphST are spatial clustering algorithms. Interestingly, the performance of the above six spatial clustering algorithms especially STMSGAL is better than the clustering method, elucidating STMSGAL's powerful spatial domain identification ability (Figure 2C).

STMSGAL manifested the distance between spatial domains and characterized the spatial trajectory in a UMAP plot [74] by integrating spatial contexts. For example, in the DLPFC section 151509, the UMAP plots delineated by STMSGAL embeddings elucidated well-organized cortical layers and consistent spatial trajectories, which was in accord with functional similarity between adjacent cortical layers and the chronological order [89]. Furthermore, in the UMAP plots delineated by SCANPY embeddings, spots that belong to different layers were not clearly divided while GraphST and STMSGAL could well divide most spots into different layers (Figure 2D). Finally, we used a trajectory inference approach named PAGA [80] to verify the inferred trajectory. The PAGA graphs depicted by both STMSGAL and GraphST embeddings had a approximately linear development trajectory from layer 1 to layer 6. In addition, the identified adjacent layers by STMSGAL and GraphST showed similarity while ones from SCANPY embeddings were mixed (Figure 2D).

We further evaluated the performance STMSGAL on the mouse visual cortex STARmap dataset, which is an image-based ST dataset at single-cell resolution and is generated by the STARmap technique [29]. mclust is a widely-used R package applied to model-based clustering through finite Gaussian mixture modelling. It is more suitable to single-cell resolution data with fewer samples, such as mouse visual cortex STARmap dataset. Thus, we used mclust for performing clustering again on STARmap. Using the gold standard annotated by experts, as shown in Figure 3, STMSGAL obtained the best ST clustering performance with ARI of 0.568 compared to SCANPY, SEDR, CCST, STAGATE, and GraphST, while STAGATE achieved the second-best ranking with ARI of 0.563 (Figure 3).

We also validated the performance of STMSGAL for identifying tissue structures on the Stereo-seq dataset from mouse embryos at E9.5. Tissue domain annotations of mouse embryos were obtained from Ref. [66].

We investigated the clustering results of STAGATE, GraphST, and STMSGAL on the E9.5_E1S1 embryo. As shown in Figure 4A, although the original annotation had 12 reference clusters, we set the number of clusters in our testing to 20 to acquire a higher resolution of tissue segmentation. The clusters identified by both STAGATE and STMSGAL matched the annotation well (Figure 4B). As shown in Table 3, however, compared to STAGATE, STMSGAL computed the smallest DB and S_Dbw and the highest CH.

Moreover, we compared the clustering results of STAGATE, GraphST, and STMSGAL on the E9.5_E2S2 mouse embryo. Here, we set the number of clusters to 13, matching the original annotation (Figure 4C). The results demonstrated that STMSGAL computed the smallest DB and S_Dbw and the highest CH (Table 3). STAGATE produced more smoother clusters but failed to reveal any fine-grained tissue complexity (Figure 4D). For example, STAGATE failed to identify cavity in the brain (domain 2). In contrast, STMSGAL's clusters better matched the annotated regions.

STMSGAL can accurately dissect spatial domains on two breast cancer tissues

Differed from the cerebral cortex with clear and known morphological boundaries, breast cancer tissues are remarkably heterogeneous and consist of complex tumor microenvironment. Consequently, manually labeling cancer data only via tumor morphology can not fully depict the complexity. Thus, we utilized STMSGAL to find spatial domains on two 10x Genomics Visium datasets with respect to Human Breast Cancer (Block A Section 1) and Human Breast Cancer (DCIS).

Particularly, Louvain clustering may produce arbitrarily badly-connected communities. In the worst case, the obtained communities may even be discontinuous, especially when performing clustering iteratively. Moreover, due to the limitation of resolution, smaller communities may be clustered into larger communities. That is, smaller communities may be hidden and cause that the obtained communities contain significant substructures.

Leiden clustering is a modified version of Louvain clustering and can yield well-connected communities based on the smart local move strategy. Cancer tissues with tumor heterogeneity contain many small substructures. Thus, we used the Leiden clustering for cancer tissues with tumor heterogeneity, such as Human Breast Cancer.

Human Breast Cancer (Block A Section 1) data have obvious intratumoral and intertumoral differences. It was manually annotated by SEDR [45] (Figure 5A) and was divided into 20 regions. It contains 4 main morphotypes: Ductal Carcinoma in Situ/Lobular Carcinoma in Situ (DCIS/LCIS), Invasive Ductal Carcinoma (IDC), tumor surrounding regions with low features of malignancy (Tumor edge), and healthy tissue (Healthy).

We compared the clustering accuracy of STMSGAL with SCANPY [67], SEDR [45], CCST [46], STAGATE [47], DeepST [49], and GraphST [50] in terms of average ARI. The results show that STMSGAL computed the best ARI, significantly outperforming five other clustering methods (Figure 5B).

Figure 5C shows spatial domains identified by SCANPY, SEDR, CCST, STAGATE, DeepST, GraphST and STMSGAL. The results demonstrate that the identified domains by STMSGAL were highly consistent with manual annotations in Figure 5A and had more regional continuity. In addition, compared with other methods, STMSGAL obtained the best clustering accuracy with ARI of 0.606. Furthermore, STMSGAL identified several sub-clusters within the tumor regions, such as spatial domains 4 and 13 (Figure 5D). Furthermore, STMSGAL identified some spatial domains with low heterogeneity (i.e., healthy regions) that were remarkably consis-

tent with the manual annotations in Figure 5A.

We also analyzed intratumoral transcriptional differences among domain 1 (DCIS/LCIS), 4, and 13 (IDC) based on differential expression analysis (Figure 4E). In domain 1, we identified three differentially expressed genes, that is, *CPB1*, *COX6X*, and *IL6ST*. *CPB1* can obviously differentiate DCIS from the other subtypes of breast cancer [90]. *COX6X* may help the differentiation between estrogen receptor-positive and estrogen receptor-negative subtypes [91]. The expression of *IL6ST* closely associates with a lower risk of invasion, metastasis and recurrence [92]. In domains 4 and 13, two differentially expressed genes *IGFBP5* and *CRISP3* have dense linkages with the treatment of mammary carcinoma [93, 94]. The knockdown of *CRISP3* can greatly inhibit the migration and invasion of mammary carcinoma cells and *ERK1/2 MAPK* signaling pathway. *CRISP3* was also considered a marker for clinical outcomes in the mammary carcinoma patients [93]. *IGFBP5* helps manage tamoxifen resistance in breast cancer [94]. The above results suggested that STMSGAL can accurately identify spatial regions with different biological functions.

We further investigated ST data on Human Breast Cancer (DCIS). Figure 6A gives its manually annotated areas. STMSGAL identified more fluent and continuous regions than other algorithms and better matched the annotated areas (Figure 6B, Supplementary Figure S2 and Table 2). Figure 6D lists the top 3 differentially expressed genes (i.e., *AZGP1*, *CD24*, and *ERBB2*) in domain 0 (Figure 6C). The expression of *AZGP1* determines the histologic grade of tumours in breast cancer [95]. *CD24* is a key indicator of triplenegative breast cancer [96, 97, 98]. In particular, the overexpression of *ERBB2* categorizes *ERBB2/HER2*-positive, a subclass of breast cancer. The subclass accounts for about 20-30% among all types of breast malignancies and is usually linked to poor prognosis [99]. Targeting *ERBB2* contributes to the treatment of *ERBB2*-positive breast cancers [100].

STMSGAL helps to better delineate the similarity between neighboring spots on Adult Mouse Brain (FFPE)

STMSGAL was still applied to provide insights into more complex tissues on a 10x Genomics Visium dataset from Adult Mouse Brain (FFPE) (Figure 7 and Supplementary Figure S3). Figure 7A shows spatial domains identified by SCANPY, DeepST, STAGATE, and STMSGAL. In the hippocampal region, the clustering results generated by SCANPY roughly separated the brain tissue structures composed of different cell types but failed to capture small spatial domains. SCANPY did not observe the “cord-like” structure (i.e., Ammon’s horn) and the “arrow-like” structure (i.e., dentate gyrus) within the hippocampus. DeepST only smoothed the spatial domain boundaries, but failed to delineate small spatial domains. STMSGAL without ctaSNN captured the Ammon’s horn, but did not characterize smaller spatial domains. However, STMSGAL with ctaSNN clearly identified both the Ammon’s horn and dentate gyrus structures in the hippocampus, in accord with annotations about the hippocampus structures from the Allen Reference Atlas [101] (Figure 7B). The above results suggested that STMSGAL significantly improved spatial domain identification. Furthermore, even for ST data composed of heterogeneous cell types with low spatial resolution, STMSGAL with ctaSNN can still accurately decipher the spatial similarity.

Additionally, the expressions of multiple known gene markers validated the cluster partitions of STMSGAL (Figure 7C and Supplementary Figure S4). For example, *C1ql2* was highly expressed on the identified DG-sg region [102]. *Hpca*, that mediates calcium-dependent translocation of brain-type creatine kinase in hippocampal neurons, was highly expressed in the Ammon’s horn region [103]. Notably, STMSGAL also captured several well-separated spatial domains and deciphered their spatial expression patterns based on differential expression analysis. Domain 15 within the hippocampus except for the “cord-like” and “arrow-like” structures delineated high expressions of two astrocytes gene markers *Mt2* and *Gfap* [104]. The spatial domain 14 surrounding the hippocampal expressed multiple oligodendrocytes-related gene markers including *Trf* and *Mbp* [105] (Supplementary Figure S4). The above results elucidated that STMSGAL can efficiently detect spatial heterogeneity and further decompose spatial expression patterns. Notably, the cell type-aware module obviously boosted the partition of tissue structures on Adult Mouse Brain (FFPE) based on its UMAP plot [74] while those of DeepST was more like a smooth version of the non-spatial method SCANPY (Figure 7D).

Finally, all attention layers of STMSGAL with ctaSNN were visualized. In each layer, nodes were arranged based on spot spatial locations, and edges were colored by corresponding weights. The results demonstrated that the combination of attention mechanism and ctaSNN boosted the characterization of the boundaries of main tissue structures on Adult Mouse Brain (FFPE) (such as the cortex, hippocampus, and midbrain) (Figure 7E). Collectively, attention mechanism and ctaSNN contributed to delineating the similarity between neighboring spots (Figure 7E).

Ablation study

In our STMSGAL method, the combination of graph attention autoencoder (GATE) and multi-scale deep subspace clustering aims to obtain multi-scale feature information of spots. The self-supervised module aims to learn robust latent features with clustering information for each spot.

To justify the contribution and necessity of these components, we conducted the ablation study to further investigate the effects of GATE, multi-scale deep subspace clustering, and the self-supervised module on spatial clustering performance on the DLPFC sections from 151507 to 151510. As shown in Table 4, $\mathcal{L}_1$ denotes the reconstruction loss of normalized expressions based on GATE. $\mathcal{L}_2$ denotes the loss of the multi-scale deep subspace clustering module, which contains regularization loss $\mathcal{L}_{reg}$ and multi-scale self-expression loss $\mathcal{L}_{mss}$, and $\mathcal{L}_3$ is the loss of the self-supervised module.

From Table 4, we found that both the multi-scale deep subspace clustering module and the self-supervised module cooperated well with GATE and greatly improved the clustering performance. The results demonstrated that the self-supervised module, which utilized the clustering labels to self-supervise the learning of spot embeddings, obtained more accurate clustering ability. And the multi-scale deep subspace clustering module fully utilized the embedded multi-scale information and manifested an obvious effect on spatial clustering, suggesting that a proper clustering-oriented loss function can efficiently enhance the clustering performance.

Moreover, to analyze the effect of the multi-scale strategy on spatial clustering performance, we compared the difference between individual self-expression layers and multi-scale self-expression layers. Table 5 gives the ARI values of STMSGAL with the multi-scale strategy or not on the DLPFC section from 151507 to 151510. We applied a controlled variable approach to make the rest modules the same. The results indicated that the performance of STMSGAL with the multi-scale strategy was better than one from single self-expression layer on the four DLPFC sections, verifying that the multi-scale strategy fully utilized the embedding features in different layers. In addition, the adaptive fusion method still significantly improved the spatial clustering performance.

Since some spots could be erroneously assigned to spatially diametrical domains and cause noises during spot embedding feature learning, we used an additional optimization step to further optimize spatial clustering results obtained from Louvain clustering

on the DLPFC dataset.

To further investigate the effect of the additional optimization step on the spatial clustering performance, we compared the performance of STMSGAL with the additional optimization step or not on Sections 151507 to 151510 of DLPFC. Table 6 gives the ARI values of STMSGAL with the additional optimization step or not on DLPFC. The results demonstrated that STMSGAL with the additional optimization step significantly outperformed STMSGAL without the step. Thus, the additional optimization step could help spatial clustering.

When performing clustering again, we used Louvian clustering on DLPFC, Leiden clustering on Human Breast Cancer, and mclust on STARmap. To analyze why to use different clustering algorithms on different datasets, we conducted ablation experiments on the above three datasets. Tables 7-8 demonstrated ablation analysis results based on different clustering methods when performing clustering again on DLPFC 10x Genomics Visium datasets, STARmap, and Human Breast Cancer (Block A Section 1), respectively. The results demonstrated that STMSGAL significantly improved ST clustering accuracy when using Louvian clustering on DLPFC, Leiden clustering on Human Breast Cancer, and mclust on STARmap.

Discussion

Accurately detecting spatial domains and identifying differentially expressed genes can greatly boost our understanding about tissue organization and biological functions. In this manuscript, we developed a spatial domain identification framework called STMSGAL based on GATE and multi-scale deep subspace clustering. STMSGAL can been accurately incorporated to the standard analysis pipeline by using the “anndata” object in the SCANPY package [67] as inputs.

Differed from classical autoencoders, STMSGAL utilized an attention mechanism in multiple hidden layers of the encoder and decoder. First, it constructed ctaSNN through Louvain clustering exclusively based on gene expression profiles. The weights of edges in the ctaSNN depicted the similarity between neighboring spots and were adaptively learned. Next, it integrated expression profiles and the constructed ctaSNN to form spot latent embedding representation based on GATE. It mainly includes spot embedding feature matrix construction, subspace clustering combining self-expression coefficient learning and affinity matrix construction, spot robust latent feature learning based on self-supervised learning. Finally, it implemented biological applications including spot clustering, differentially expression analysis, and trajectory inference.

In the STMSGAL method, the multi-scale self-expression module was used to fully explore the associations between spot representations in all encoder layers. The deep subspace clustering module was utilized to obtain the clustering labels for each spot through a clustering-oriented loss function. And the self-supervised module was introduced to effectively learn spot latent representation. The combination of the above three modules helps to learn more discriminative features with clustering information for each spot. And then the obtained more discriminative features with clustering information was used to be as the input of spectral clustering and conduct the final clustering.

Traditional subspace clustering mainly contains two procedures: constructing affinity matrix through representation learning and spectral clustering. However, the spectral clustering is sensitive to the construction of similarity matrix and the selection of various parameters. But the Leiden/Louvain/mclust clustering methods are more appropriate to biological data and exhibit powerful spatial clustering performance. Consequently, the Leiden/Louvain/mclust clustering has been widely used in the field of spatial clustering. Thus, our proposed STMSGAL framework used Leiden/Louvain/mclust for performing clustering again to identify spatial domains after obtaining more discriminative features with clustering information based on multi-scale deep subspace clustering.

We compared the performance of STMSGAL with seven other clustering methods on four 10x Genomics Visium datasets from Adult Mouse Brain (FFPE), Human Breast Cancer (DCIS), Human Breast Cancer (Block A Section 1), and the DLPFC tissues, as well as one mouse visual cortex STARmap dataset. The seven comparison methods include SCANPY, GraphST, SEDR, CCST, STAGATE, DeepST, and SiGra. The SCANPY has been widely applied to single-cell clustering. The remaining are state-of-the-art spatial clustering methods. The results demonstrated that our proposed STMSGAL method obtained impressive performance over other competing methods in terms of four evaluation metrics (i.e., DB, CH, S_Dbw, and ARI). STMSGAL significantly improved the identification of layer structures in four DLPFC sections, mouse visual cortex STARmap data, and mouse embryos data, accurately dissected spatial domains on two breast cancer tissues, and efficiently depicted the similarity between neighboring spots on Adult Mouse Brain (FFPE).

STMSGAL greatly boosted ST data analysis. It may be mainly attributed to the following features: First, although existing methods (such as stLearn) took histological images as inputs, they achieved limited performance. For example, stLearn adopted a pre-trained neural network to obtain spot features from images and further computed their morphological distances via cosine distance. However, the pre-defined strategy in stLearn was not flexible and resulted in its poor spatial clustering performance. In contrast, STMSGAL adopted an attention mechanism to adaptively integrate spatial locations and gene expression profiles.

Second, a multi-scale self-expression module was designed to train a self-expression coefficient matrix in different encoder layers. SEDR and CCST merely adopted the representations in the encoder final hidden layer for spatial clustering tasks, wasting much useful information embedded in its other layers. Comparatively, the multi-scale self-expression module fully explored the associations between node representations in all encoder layers. Thus, it fully adopted the embedded multi-scale information and obtained a more distinct self-expression coefficient matrix. Furthermore, it mapped these features into a more precise subspace for spatial clustering.

Finally, deep subspace clustering module was proposed to obtain the clustering labels with a clustering-oriented loss function. And a self-supervised module was introduced to effectively guide spot latent representation learning. Thus, the learned spot latent embedding representation greatly improved the clustering performance.

In summary, STMSGAL is a powerful spatial clustering framework that constructs an integrated representation for spots by aggregating both transcriptomic data and spatial context. STMSGAL derived low-dimensional embedding, enabling to conduct spatial clustering and trajectory inference more accurately. Moreover, STMSGAL facilitates to decipher new principles in spatially organized context.

Although STMSGAL achieved accurate spatial clustering performance, the deep subspace clustering algorithm can be further developed. In the near future, motivated by the linkages between spatial domain identification and single-cell segmentation used to image-based ST data, we anticipate that STMSGAL can be further extended for single-cell segmentation task applied to the subcellular resolution technologies. We also hope to enhance its applicability on other datasets generated by new sequencing technologies.

Moreover, self-supervised learning can effectively learn spot representations, but optimizing the spot representations by combining the pseudo labels can affect the convergence of the model. The contrastive learning algorithm is a promising paradigm of the self-supervised learning model. In the future, we will introduce

contrastive learning to facilitate spot representation learning and spatial clustering.

Finally, the accumulation of ST data generates spatial omics big data, which pose many technical challenges to data integration and analysis. To enable STMSGAL to deal with larger datasets, we will further alleviate the computational burden of STMSGAL using a graph convolutional network mini-batch or parallel techniques to construct large-scale graphs for spatial omics data.

Availability of source code and requirements

- Project name: STMSGAL
- Project home page: https://github.com/plhhnu/STMSGAL
- Operating system(s): Platform independent
- Programming language: Python
- License: MIT license for the code, Creative Commons CC0 1.0 Public Domain Dedication for the filtered spatial transcriptomic data
- RRID: SCR_025422
- biotools: stmsgal

Additional Files

Supplementary Figure S1. Comparison of spatial domains identified by SCANPY, SEDR, CCST, DeepST, STAGATE, SiGra, GraphST, and STMSGAL, and manual annotations in 3 sections of human DLPFC tissues.

Supplementary Figure S2. Cluster assignments generated by SCANPY, SEDR, CCST, STAGATE, DeepST, GraphST, and STMSGAL on Human Breast Cancer (Ductal Carcinoma In Situ (DCIS)).

Supplementary Figure S3. Cluster assignments generated by SCANPY, SEDR, CCST, STAGATE, DeepST, GraphST, and STMSGAL on Adult Mouse Brain (FFPE).

Supplementary Figure S4. Visualizations of spatial domains and expressions of the corresponding marker genes identified by STMSGAL with Louvain clustering on adult mouse hippocampus tissue.

Data availability

Source codes and datasets of STMSGAL are available in the GitHub repository [106]. Specifically, the DLPFC dataset is accessible within the spatialLIBD package [65]. The Adult Mouse Brain (FFPE), Human Breast Cancer (DCIS) and Human Breast Cancer (Block A Section 1) datasets are collected from the 10x Genomics website [62, 63, 64]. An archival copy of the code and supporting data is also available via the GigaScience repository, GigaDB [107]. DOME-ML (Data, Optimization, Model and Evaluation in Machine Learning) annotations are available via a link in GigaDB [107].

Declarations

Abbreviations

scRNA-seq: single-cell RNA sequencing; ST: spatial transcriptomics; NGS-based: next-generation sequencing-based; HDST: High-definition spatial transcriptomics; GATE: graph attention autoencoder; SNN: spatial neighbor network; DCIS: Ductal Carcinoma In Situ; ELU: exponential linear unit; ReLu: rectified linear units; ARI: adjusted rand index; DB: Davies-Bouldin; CH: Calinski-Harabasz; DCIS/LCIS: Ductal Carcinoma in Situ/Lobular Carcinoma in Situ; IDC: Invasive Ductal Carcinoma.

Competing Interests

G. Tian and J-L Yang are employees and shareholders of Geneis (Beijing) Co. Ltd. The remaining authors declare no competing interests.

Funding

Liqian Zhou was funded by National Natural Science Foundation of China under Grant No. 62072172 and Natural Science Foundation of Hunan province under Grant No. 2021JJ30219. Min Chen was supported by National Natural Science Foundation of China under Grant No. 62172158. Lihong Peng was supported by National Natural Science Foundation of China under Grant No. 61803151 and Natural Science Foundation of Hunan province under Grant No. 2023JJ50201.

Author's Contributions

Conceptualization: L.Q.Z., X.H.P., L.H.P., M.C., and J.L.Y. Methodology: L.Q.Z., X.H.P., X.Z.H., and G.T. Supervision: L.H.P., J.L.Y. Writing—original draft: L.Q.Z., L.H.P., and X.H.P. Writing—review and editing: L.Q.Z., X.H.P., L.H.P., M.C., and J.L.Y.

Acknowledgements

We really appreciate reviewers for their valuable comments. We would like to thank all authors of the cited references.

References

1. Hu J, Schroeder A, Coleman K, Chen C, Auerbach BJ, Li M. Statistical and machine learning methods for spatially resolved transcriptomics with histology. Computational and Structural Biotechnology Journal 2021;19:3829–3841.
2. Ren X, Wen W, Fan X, Hou W, Su B, Cai P, et al. COVID-19 immune features revealed by a large-scale single-cell transcriptome atlas. Cell 2021;184(7):1895–1913.
3. Yang W, Wang P, Luo M, Cai Y, Xu C, Xue G, et al. DeepCCI: a deep learning framework for identifying cell–cell interactions from single-cell RNA sequencing data. Bioinformatics 2023;39(10):btad596.
4. Qi R, Wu J, Guo F, Xu L, Zou Q. A spectral clustering with self-weighted multiple kernel learning method for single-cell RNA-seq data. Briefings in Bioinformatics 2021;22(4):bbaa216.
5. Xu J, Xu J, Meng Y, Lu C, Cai L, Zeng X, et al. Graph embedding and Gaussian mixture variational autoencoder network for end-to-end analysis of single-cell RNA sequencing data. Cell Reports Methods 2023;3(1):100382.
6. Peng L, Yuan R, Han C, Han G, Tan J, Wang Z, et al. Cellenboost: a boosting-based ligand-receptor interaction identification model for cell-to-cell communication inference. IEEE Transactions on NanoBioscience 2023;22(4):705–715.
7. Peng L, Tan J, Xiong W, Zhang L, Wang Z, Yuan R, et al. Deciphering ligand–receptor-mediated intercellular communication based on ensemble deep learning and the joint scoring strategy from single-cell transcriptomic data. Computers in Biology and Medicine 2023;163:107137.
8. Peng L, Xiong W, Han C, Li Z, Chen X. CellDialog: A Computational Framework for Ligand-receptor-mediated Cell-cell Communication Analysis. IEEE Journal of Biomedical and Health Informatics 2024;28(2):1–12.
9. Peng L, Gao P, Xiong W, Li Z, Chen X. Identifying potential ligand–receptor interactions based on gradient boosted

neural network and interpretable boosting machine for intercellular communication analysis. Computers in Biology and Medicine 2024;171:108110.
10. Jing J, Feng J, Yuan Y, Guo T, Lei J, Pei F, et al. Spatiotemporal single-cell regulatory atlas reveals neural crest lineage diversification and cellular function during tooth morphogenesis. Nature Communications 2022;13(1):4803.
11. Shang L, Zhou X. Spatially aware dimension reduction for spatial transcriptomics. Nature Communications 2022;13(1):7203.
12. Peng L, Huang L, Su Q, Tian G, Chen M, Han G. LDA-VGHB: identifying potential lncRNA-disease associations with singular value decomposition, variational graph auto-encoder and heterogeneous Newton boosting machine. Briefings In Bioinformatics 2024;5(1):bbad466.
13. Peng L, Ren M, Huang L, Chen M. GEnDDn: An lncRNA–Disease Association Identification Framework Based on Dual-Net Neural Architecture and Deep Neural Network. Interdisciplinary Sciences: Computational Life Sciences 2024;p. 1–21.
14. Svensson V, Vento-Tormo R, Teichmann SA. Exponential scaling of single-cell RNA-seq in the past decade. Nature protocols 2018;13(4):599–604.
15. Ding Q, Yang W, Luo M, Xu C, Xu Z, Pang F, et al. CBLRR: a cauchy-based bounded constraint low-rank representation method to cluster single-cell RNA-seq data. Briefings in Bioinformatics 2022;23(5):bbac300.
16. Wang P, Yao L, Luo M, Zhou W, Jin X, Xu Z, et al. Single-cell transcriptome and TCR profiling reveal activated and expanded T cell populations in Parkinson's disease. Cell Discovery 2021;7(1):52.
17. Wang J, Zou Q, Lin C. A comparison of deep learning-based pre-processing and clustering approaches for single-cell RNA sequencing data. Briefings in Bioinformatics 2022;23(1):bbab345.
18. Yan S, Si Y, Zhou W, Cheng R, Wang P, Wang D, et al. Single-cell transcriptomics reveals the interaction between peripheral CD4+ CTLs and mesencephalic endothelial cells mediated by IFNG in Parkinson's disease. Computers in Biology and Medicine 2023;158:106801.
19. Hu H, Feng Z, Lin H, Cheng J, Lyu J, Zhang Y, et al. Gene function and cell surface protein association analysis based on single-cell multiomics data. Computers in Biology and Medicine 2023;157:106733.
20. Tang X, Zhou C, Lu C, Meng Y, Xu J, Hu X, et al. Enhancing drug repositioning through local interactive learning with bilinear attention networks. IEEE Journal of Biomedical and Health Informatics 2023;.
21. Xu J, Xu J, Meng Y, Lu C, Cai L, Zeng X, et al. Graph embedding and Gaussian mixture variational autoencoder network for end-to-end analysis of single-cell RNA sequencing data. Cell Reports Methods 2023;p. 100382.
22. Yang Q, Xu Z, Zhou W, Wang P, Jiang Q, Juan L. An interpretable single-cell RNA sequencing data clustering method based on latent Dirichlet allocation. Briefings in Bioinformatics 2023;p. bbad199.
23. Zhang C, Gao J, Chen HY, Kong L, Cao G, Guo X, et al. STGIC: A graph and image convolution-based method for spatial transcriptomic clustering. PLOS Computational Biology 2024;20(2):e1011935.
24. Qiu Y, Yan C, Zhao P, Zou Q. SSNMDI: a novel joint learning model of semi-supervised non-negative matrix factorization and data imputation for clustering of single-cell RNA-seq data. Briefings in Bioinformatics 2023;24(3):bbad149.
25. Wang P, Luo M, Zhou W, Jin X, Xu Z, Yan S, et al. Global characterization of peripheral B cells in Parkinson's disease by single-cell RNA and BCR sequencing. Frontiers in Immunology 2022;13:814239.
26. Jiang J, Xu J, Liu Y, Song B, Guo X, Zeng X, et al. Dimensionality reduction and visualization of single-cell RNA-seq data with an improved deep variational autoencoder. Briefings in Bioinformatics 2023;24(3):bbad152.
27. Rao A, Barkley D, França GS, Yanai I. Exploring tissue architecture using spatial transcriptomics. Nature 2021;596(7871):211–220.
28. Moffitt JR, Hao J, Wang G, Chen KH, Babcock HP, Zhuang X. High-throughput single-cell gene-expression profiling with multiplexed error-robust fluorescence in situ hybridization. Proceedings of the National Academy of Sciences 2016;113(39):11046–11051.
29. Wang X, Allen WE, Wright MA, Sylwestrak EL, Samusik N, Vesuna S, et al. Three-dimensional intact-tissue sequencing of single-cell transcriptional states. Science 2018;361(6400):eaat5691.
30. Eng CHL, Lawson M, Zhu Q, Dries R, Koulena N, Takei Y, et al. Transcriptome-scale super-resolved imaging in tissues by RNA seqFISH+. Nature 2019;568(7751):235–239.
31. Larsson L, Frisén J, Lundeberg J. Spatially resolved transcriptomics adds a new dimension to genomics. Nature methods 2021;18(1):15–18.
32. Rodriques SG, Stickels RR, Goeva A, Martin CA, Murray E, Vanderburg CR, et al. Slide-seq: A scalable technology for measuring genome-wide expression at high spatial resolution. Science 2019;363(6434):1463–1467.
33. Stickels RR, Murray E, Kumar P, Li J, Marshall JL, Di Bella DJ, et al. Highly sensitive spatial transcriptomics at near-cellular resolution with Slide-seqV2. Nature biotechnology 2021;39(3):313–319.
34. Vickovic S, Eraslan G, Salmén F, Klughammer J, Stenbeck L, Schapiro D, et al. High-definition spatial transcriptomics for in situ tissue profiling. Nature methods 2019;16(10):987–990.
35. Liu Y, Yang M, Deng Y, Su G, Enninful A, Guo CC, et al. High-spatial-resolution multi-omics sequencing via deterministic barcoding in tissue. Cell 2020;183(6):1665–1681.
36. Xia K, Sun HX, Li J, Li J, Zhao Y, Chen R, et al. Single-cell Stereo-seq enables cell type-specific spatial transcriptome characterization in Arabidopsis leaves. bioRxiv;.
37. 10x Genomics;. https://www.10xgenomics.com/resources/datasets.
38. Maynard KR, Collado-Torres L, Weber LM, Uytingco C, Barry BK, Williams SR, et al. Transcriptome-scale spatial gene expression in the human dorsolateral prefrontal cortex. Nature neuroscience 2021;24(3):425–436.
39. Hartigan JA, Wong MA. Algorithm AS 136: A k-means clustering algorithm. Journal of the royal statistical society series c (applied statistics) 1979;28(1):100–108.
40. Blondel VD, Guillaume JL, Lambiotte R, Lefebvre E. Fast unfolding of communities in large networks. Journal of statistical mechanics: theory and experiment 2008;2008(10):P10008.
41. Elosua-Bayes M, Nieto P, Mereu E, Gut I, Heyn H. SPOTlight: seeded NMF regression to deconvolute spatial transcriptomics spots with single-cell transcriptomes. Nucleic acids research 2021;49(9):e50–e50.
42. Cable DM, Murray E, Zou LS, Goeva A, Macosko EZ, Chen F, et al. Robust decomposition of cell type mixtures in spatial transcriptomics. Nature Biotechnology 2022;40(4):517–526.
43. Zhao E, Stone MR, Ren X, Guenthoer J, Smythe KS, Pulliam T, et al. Spatial transcriptomics at subspot resolution with BayesSpace. Nature biotechnology 2021;39(11):1375–1384.
44. Pham D, Tan X, Balderson B, Xu J, Grice LF, Yoon S, et al. Robust mapping of spatiotemporal trajectories and cell–cell interactions in healthy and diseased tissues. Nature communications 2023;14(1):7739.
45. Fu H, Xu H, Chong K, Li M, Ang KS, Lee HK, et al. Unsupervised spatially embedded deep representation of spatial tran-

scriptomics. Biorxiv 2021; https://doi.org/10.1101/2021.06.15.448542.
46. Li J, Chen S, Pan X, Yuan Y, Shen Hb. CCST: Cell clustering for spatial transcriptomics data with graph neural network 2021;.
47. Dong K, Zhang S. Deciphering spatial domains from spatially resolved transcriptomics with an adaptive graph attention auto-encoder. Nature communications 2022;13(1):1–12.
48. Wang T, Sun J, Zhao Q. Investigating cardiotoxicity related with hERG channel blockers using molecular fingerprints and graph attention mechanism. Computers in Biology and Medicine 2023;153:106464.
49. Xu C, Jin X, Wei S, Wang P, Luo M, Xu Z, et al. DeepST: identifying spatial domains in spatial transcriptomics by deep learning. Nucleic Acids Research 2022;50(22):e131–e131.
50. Long Y, Ang KS, Li M, Chong KLK, Sethi R, Zhong C, et al. Spatially informed clustering, integration, and deconvolution of spatial transcriptomics with GraphST. Nature Communications 2023;14(1):1155.
51. Meng Y, Wang Y, Xu J, Lu C, Tang X, Peng T, et al. Drug repositioning based on weighted local information augmented graph neural network. Briefings in Bioinformatics 2024;25(1):bbad431.
52. Zeng P, Zhang B, Liu A, Meng Y, Tang X, Yang J, et al. Drug repositioning based on tripartite cross-network embedding and graph convolutional network. Expert Systems with Applications 2024;252:124152.
53. Zeng Y, Yin R, Luo M, Chen J, Pan Z, Lu Y, et al. Identifying spatial domain by adapting transcriptomics with histology through contrastive learning. Briefings in Bioinformatics 2023;24(2):bbad048.
54. Zhang C, Gao J, Liu W, et al. STGIC: a graph and image convolution-based method for spatial transcriptomic clustering. arXiv preprint arXiv:230310657 2023;.
55. Xu H, Lin J, Wang S, Fang M, Luo S, Chen C, et al. SPACEL: characterizing spatial transcriptome architectures by deep-learning 2023;.
56. Liu W, Liao X, Luo Z, Yang Y, Lau MC, Jiao Y, et al. Probabilistic embedding, clustering, and alignment for integrating spatial transcriptomics data with PRECAST. Nature Communications 2023;14(1):296.
57. Zhu J, Shang L, Zhou X. SRTsim: spatial pattern preserving simulations for spatially resolved transcriptomics. Genome Biology 2023;24(1):39.
58. Tang Z, Li Z, Hou T, Zhang T, Yang B, Su J, et al. SiGra: single-cell spatial elucidation through an image-augmented graph transformer. Nature Communications 2023;14(1):5618.
59. Yuan Z, Zhao F, Lin S, Zhao Y, Yao J, Cui Y, et al. Benchmarking spatial clustering methods with spatially resolved transcriptomics data. Nature Methods 2024;21(4):712–722.
60. Salehi A, Davulcu H. Graph attention auto-encoders. arXiv preprint arXiv:190510715 2019; https://doi.org/10.48550/arXiv.1905.10715.
61. Kou S, Xia W, Zhang X, Gao Q, Gao X. Self-supervised graph convolutional clustering by preserving latent distribution. Neurocomputing 2021;437:218–226.
62. Adult Mouse Brain (FFPE);. https://www.10xgenomics.com/datasets/adult-mouse-brain-ffpe-1-standard-1-3-0.
63. Human Breast Cancer (Block A Section 1);. https://www.10xgenomics.com/datasets/human-breast-cancer-block-a-section-1-1-standard-1-1-0.
64. Human Breast Cancer (DCIS);. https://www.10xgenomics.com/datasets/human-breast-cancer-ductal-carcinoma-in-situ-invasive-carcinoma-ffpe-1-standard-1-3-0.
65. Human dorsolateral prefrontal cortex dataset;. http://spatial.libd.org/spatialLIBD/.
66. Chen A, Liao S, Cheng M, Ma K, Wu L, Lai Y, et al. Spatiotemporal transcriptomic atlas of mouse organogenesis using DNA nanoball-patterned arrays. Cell 2022;185(10):1777–1792.
67. Wolf FA, Angerer P, Theis FJ. SCANPY: large-scale single-cell gene expression data analysis. Genome biology 2018;19(1):1–5.
68. Wang T, Wu J, Zhang Z, Zhou W, Chen G, Liu S. Multi-scale graph attention subspace clustering network. Neurocomputing 2021;459:302–314.
69. Veličković P, Cucurull G, Casanova A, Romero A, Lio P, Bengio Y. Graph attention networks. arXiv preprint arXiv:171010903 2017; https://doi.org/10.48550/arXiv.1710.10903.
70. Ji P, Zhang T, Li H, Salzmann M, Reid I. Deep subspace clustering networks. Advances in neural information processing systems 2017;30.
71. Ng A, Jordan M, Weiss Y. On spectral clustering: Analysis and an algorithm. Advances in neural information processing systems 2001;14.
72. Traag VA, Waltman L, Van Eck NJ. From Louvain to Leiden: guaranteeing well-connected communities. Scientific reports 2019;9(1):1–12.
73. Fraley C, Raftery AE, Murphy TB, Scrucca L. mclust version 4 for R: normal mixture modeling for model-based clustering, classification, and density estimation. Technical report; 2012.
74. McInnes L, Healy J, Melville J. Umap: Uniform manifold approximation and projection for dimension reduction. arXiv preprint arXiv:180203426 2018; https://doi.org/10.48550/arXiv.1802.03426.
75. Mou T, Deng W, Gu F, Pawitan Y, Vu TN. Reproducibility of methods to detect differentially expressed genes from single-cell RNA sequencing. Frontiers in genetics 2020;10:1331.
76. Vu TN, Wills QF, Kalari KR, Niu N, Wang L, Rantalainen M, et al. Beta-Poisson model for single-cell RNA-seq data analyses. Bioinformatics 2016;32(14):2128–2135.
77. Das S, Rai SN. SwarnSeq: An improved statistical approach for differential expression analysis of single-cell RNA-seq data. Genomics 2021;113(3):1308–1324.
78. Cannoodt R, Saelens W, Saeys Y. Computational methods for trajectory inference from single-cell transcriptomics. European journal of immunology 2016;46(11):2496–2506.
79. Trapnell C, Cacchiarelli D, Grimsby J, Pokharel P, Li S, Morse M, et al. The dynamics and regulators of cell fate decisions are revealed by pseudotemporal ordering of single cells. Nature biotechnology 2014;32(4):381–386.
80. Wolf FA, Hamey FK, Plass M, Solana J, Dahlin JS, Göttgens B, et al. PAGA: graph abstraction reconciles clustering with trajectory inference through a topology preserving map of single cells. Genome biology 2019;20:1–9.
81. Clevert DA, Unterthiner T, Hochreiter S. Fast and accurate deep network learning by exponential linear units (elus). arXiv preprint arXiv:151107289 2015; https://doi.org/10.48550/arXiv.1511.07289.
82. Kingma DP, Ba J. Adam: A method for stochastic optimization. arXiv preprint arXiv:14126980 2014; https://doi.org/10.48550/arXiv.1412.6980.
83. Nair V, Hinton GE. Rectified linear units improve restricted boltzmann machines. In: Icml; 2010. .
84. Hubert L, Arabie P. Comparing partitions. Journal of classification 1985;2(1):193–218.
85. Davies DL, Bouldin DW. A cluster separation measure. IEEE transactions on pattern analysis and machine intelligence 1979;(2):224–227.
86. Caliński T, Harabasz J. A dendrite method for cluster analysis. Communications in Statistics-theory and Methods 1974;3(1):1–27.
87. Halkidi M, Vazirgiannis M. Clustering validity assessment: Finding the optimal partitioning of a data set. In: Proceedings 2001 IEEE international conference on data mining IEEE; 2001. p. 187–194.

88. Peng L, He X, Peng X, Li Z, Zhang L. STGNNks: Identifying cell types in spatial transcriptomics data based on graph neural network, denoising auto-encoder, and k-sums clustering. Computers in Biology and Medicine 2023;166:107440.
89. Gilmore EC, Herrup K. Cortical development: layers of complexity. Current Biology 1997;7(4):R231–R234.
90. Kothari C, Clemenceau A, Ouellette G, Ennour-Idrissi K, Michaud A, Diorio C, et al. Is carboxypeptidase B1 a prognostic marker for ductal carcinoma in situ? Cancers 2021;13(7):1726.
91. Gruvberger S, Ringnér M, Chen Y, Panavally S, Saal LH, Borg A, et al. Estrogen receptor status in breast cancer is associated with remarkably distinct gene expression patterns. Cancer research 2001;61(16):5979–5984.
92. Martínez-Pérez C, Leung J, Kay C, Meehan J, Gray M, Dixon JM, et al. The signal transducer IL6ST (gp130) as a predictive and prognostic biomarker in breast cancer. Journal of personalized medicine 2021;11(7):618.
93. Wang Y, Sheng N, Xie Y, Chen S, Lu J, Zhang Z, et al. Low expression of CRISP3 predicts a favorable prognosis in patients with mammary carcinoma. Journal of Cellular Physiology 2019;234(8):13629–13638.
94. Ahn BY, Elwi AN, Lee B, Trinh DL, Klimowicz AC, Yau A, et al. Genetic Screen Identifies Insulin-like Growth Factor Binding Protein 5 as a Modulator of Tamoxifen Resistance in Breast CancerIGFBP5 Reverses Tamoxifen Resistance. Cancer research 2010;70(8):3013–3019.
95. Díez-Itza I, Sánchez LM, Allende MT, Vizoso F, Ruibal A, López-Otín C. Zn-α2-glycoprotein levels in breast cancer cytosols and correlation with clinical, histological and biochemical parameters. European Journal of Cancer 1993;29(9):1256–1260.
96. Zhang P, Zheng P, Liu Y. Amplification of the CD24 gene is an independent predictor for poor prognosis of breast cancer. Frontiers in Genetics 2019;10:560.
97. Kwon MJ, Han J, Seo JH, Song K, Jeong HM, Choi JS, et al. CD24 overexpression is associated with poor prognosis in luminal A and triple-negative breast cancer. PLoS One 2015;10(10):e0139112.
98. Sheridan C, Kishimoto H, Fuchs RK, Mehrotra S, Bhat-Nakshatri P, Turner CH, et al. CD44+/CD24-breast cancer cells exhibit enhanced invasive properties: an early step necessary for metastasis. Breast Cancer Research 2006;8:1–13.
99. Segovia-Mendoza M, González-González ME, Barrera D, Díaz L, García-Becerra R. Efficacy and mechanism of action of the tyrosine kinase inhibitors gefitinib, lapatinib and neratinib in the treatment of HER2-positive breast cancer: preclinical and clinical evidence. American journal of cancer research 2015;5(9):2531.
100. Rimawi MF, Schiff R, Osborne CK. Targeting HER2 for the treatment of breast cancer. Annual review of medicine 2015;66.
101. Sunkin SM, Ng L, Lau C, Dolbeare T, Gilbert TL, Thompson CL, et al. Allen Brain Atlas: an integrated spatio-temporal portal for exploring the central nervous system. Nucleic acids research 2012;41(D1):D996–D1008.
102. Iijima T, Miura E, Watanabe M, Yuzaki M. Distinct expression of C1q-like family mRNAs in mouse brain and biochemical characterization of their encoded proteins. European Journal of Neuroscience 2010;31(9):1606–1615.
103. Kobayashi M, Hamanoue M, Masaki T, Furuta Y, Takamatsu K. Hippocalcin mediates calcium-dependent translocation of brain-type creatine kinase (BB-CK) in hippocampal neurons. Biochemical and Biophysical Research Communications 2012;429(3-4):142–147.
104. Li D, Liu X, Liu T, Liu H, Tong L, Jia S, et al. Neurochemical regulation of the expression and function of glial fibrillary acidic protein in astrocytes. Glia 2020;68(5):878–897.
105. White R, Gonsior C, Krämer-Albers EM, Stöhr N, Hüttelmaier S, Trotter J. Activation of oligodendroglial Fyn kinase enhances translation of mRNAs transported in hnRNP A2–dependent RNA granules. The Journal of cell biology 2008;181(4):579–586.
106. GitHub repository;. https://github.com/plhhnu/STMSGAL.
107. Zhou L, Peng X, Chen M, He X, Tian G, Yang J, et al., Supporting data for "Unveiling Patterns in Spatial Transcriptomics Data: A Novel Approach Utilizing Graph Attention Autoencoder and Multi-Scale Deep Subspace Clustering Network". GigaScience Database; 2024. http://gigadb.org/dataset/102582.

Figures and Tables

Table 1. Parameter settings

Datasets	Parameter settings
DLPFC	rad_cutoff = 150<br>cost_ssc = 0.1<br>α = 0<br>method = 'Louvain'
Human Breast Cancer (Block A Section 1)	rad_cutoff = 300<br>cost_ssc = 1<br>α = 0.7<br>method = 'leiden'
Adult Mouse Brain (FFPE)	rad_cutoff = 300<br>cost_ssc = 0.1<br>α = 0.5<br>method = 'Louvain'
Human Breast Cancer (DCIS)	rad_cutoff = 300<br>cost_ssc = 1<br>α = 0.5<br>method = 'Louvain'
Mouse visual cortex	rad_cutoff = 400<br>cost_ssc = 0.1<br>α = 0<br>method = 'mclust'
Stereo-seq mouse embryo	rad_cutoff = 3<br>cost_ssc = 0.1<br>α = 0<br>method = 'Louvain'

Table 2. Performance comparison of STMSGAL with six other clustering methods on Adult Mouse Brain (FFPE) and Human Breast Cancer (Ductal Carcinoma In Situ (DCIS)).

Datasets	Methods	Metrics		
		DB	CH	S_dbw
Adult Mouse Brain (FFPE)	SCANPY	1.442	358.67	0.481
	SEDR	1.951	84.569	0.652
	CCST	1.173	507.421	0.453
	DeepST	1.166	842.033	0.328
	STAGATE	1.467	495.547	0.427
	GraphST	1.470	310.860	0.501
	STMSGAL	**1.155**	**1010.724**	**0.311**
Human Breast Cancer (DCIS)	SCANPY	2.069	379.084	0.593
	SEDR	2.627	54.778	0.742
	CCST	1.469	507.421	0.453
	DeepST	**1.263**	611.567	0.48
	STAGATE	1.916	430.630	0.587
	GraphST	1.951	369.594	0.610
	STMSGAL	1.451	**1190.850**	**0.332**

** The bold font indicates the best performance in each column. Lower Davies-Bouldin (DB) and S_Dbw and higher Calinski-Harabasz (CH) denote better performance.*

Table 3. Performance comparison of STMSGAL with STAGATE and GraphST on the Stereo-seq mouse embryos.

Datasets	Methods	Metrics		
		DB	CH	S_dbw
E9,5_E1S1	STAGATE	1.579	582.733	0.585
	GraphST	**1.396**	686.177	0.549
	STMSGAL	1.957	**1915.695**	**0.355**
E9,5_E2S2	STAGATE	1.708	603.290	0.608
	GraphST	**1.686**	563.371	0.632
	STMSGAL	1.861	**1171.402**	**0.488**

** The bold font indicates the best performance in each column. Lower Davies-Bouldin (DB) and S_Dbw and higher Calinski-Harabasz (CH) denote better performance.*

Table 4. Ablation study on different loss terms.

Datasets	Loss Function			ARI
	$\mathcal{L}_1$	$\mathcal{L}_2$	$\mathcal{L}_3$	
151507	○	×	×	0.508
	○	○	×	0.518
	○	○	○	**0.533**
151508	○	×	×	0.405
	○	○	×	0.444
	○	○	○	**0.473**
151509	○	×	×	0.392
	○	○	×	0.447
	○	○	○	**0.511**
151510	○	×	×	0.437
	○	○	×	0.442
	○	○	○	**0.452**

** The bold type indicates the best performance in each column.*

Table 5. Ablation study on the multi-scale strategy.

Datasets	Strategy	ARI
151507	Without the multi-scale strategy	0.485
	With the multi-scale strategy	**0.533**
151508	Without the multi-scale strategy	0.394
	With the multi-scale strategy	**0.473**
151509	Without the multi-scale strategy	0.437
	With the multi-scale strategy	**0.511**
151510	Without the multi-scale strategy	0.407
	With the multi-scale strategy	**0.452**

** The bold type indicates the best performance in each column.*

Table 6. Ablation study on the additional optimization step.

Datasets	Strategy	ARI
151507	Without the additional optimization step	0.509
	With the additional optimization step	**0.533**
151508	Without the additional optimization step	0.450
	With the additional optimization step	**0.473**
151509	Without the additional optimization step	0.484
	With the additional optimization step	**0.511**
151510	Without the additional optimization step	0.430
	With the additional optimization step	**0.452**

** The bold type indicates the best performance in each column.*

Table 7. Ablation analysis under different clustering methods on DLPFC 10x Genomics Visium datasets.

Methods	Datasets				Average ARI
	151507	151508	151509	151510	
Louvain clustering	**0.533**	0.473	**0.511**	**0.452**	**0.492**
mclust	0.520	0.475	0.354	0.403	0.438
Leiden clustering	0.511	**0.489**	0.471	0.393	0.469
subspace clustering	0.216	0.325	0.395	0.284	0.294

** The bold type indicates the best performance in each column.*

Table 8. Ablation analysis under different clustering methods on STARmap and Human Breast Cancer (Block A Section 1).

Datasets	Methods	ARI
STARmap	Louvain clustering	0.282
	mclust	**0.568**
	Leiden clustering	0.273
	subspace clustering	0.067
Human Breast Cancer (Block A Section 1)	Louvain clustering	0.534
	mclust	0.512
	Leiden clustering	**0.606**
	subspace clustering	0.588

** The bold type indicates the best performance in each column.*

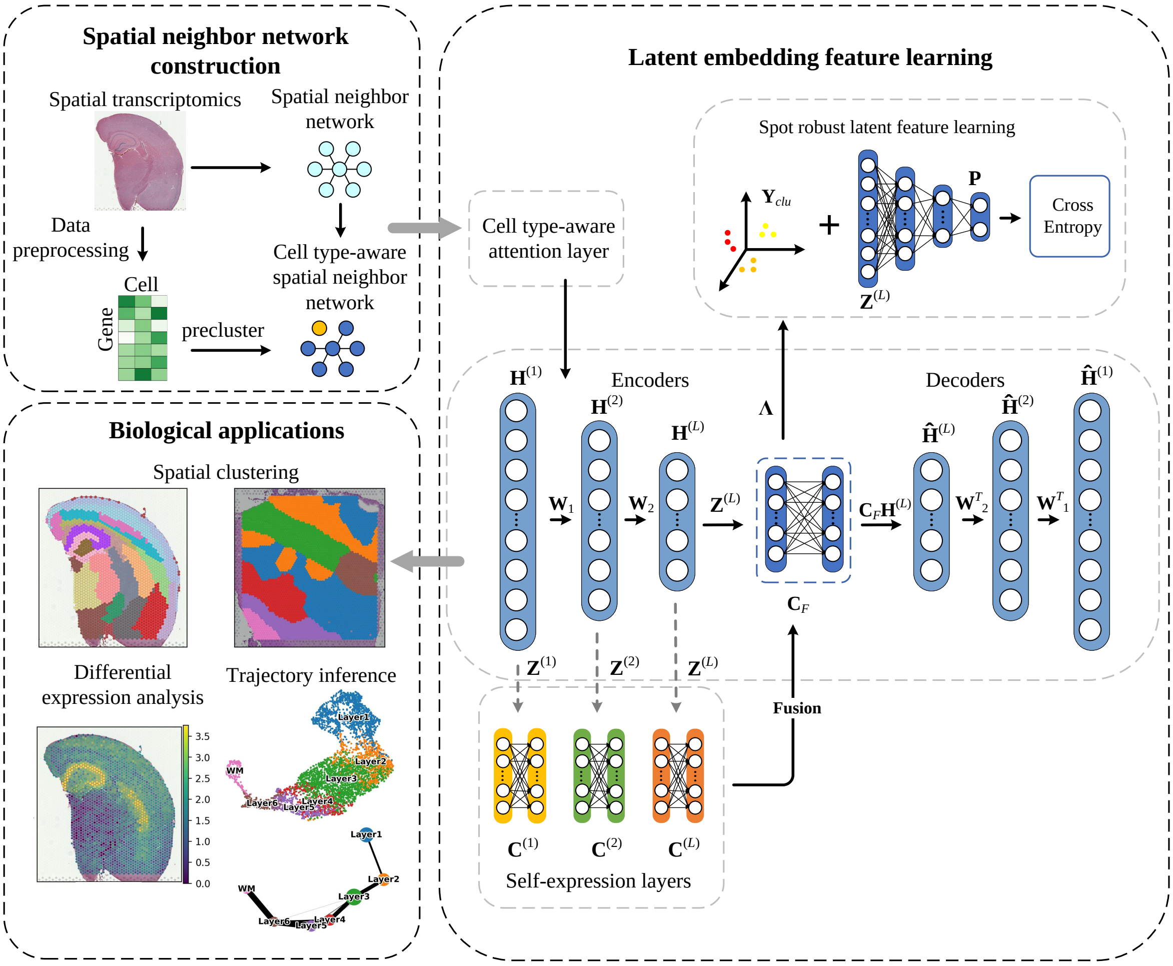

Figure 1. Pipeline for clustering ST data based on GATE and deep subspace clustering network. (i) Spatial neighbor network construction. (ii) Latent embedding feature learning. (iii) Biological applications.

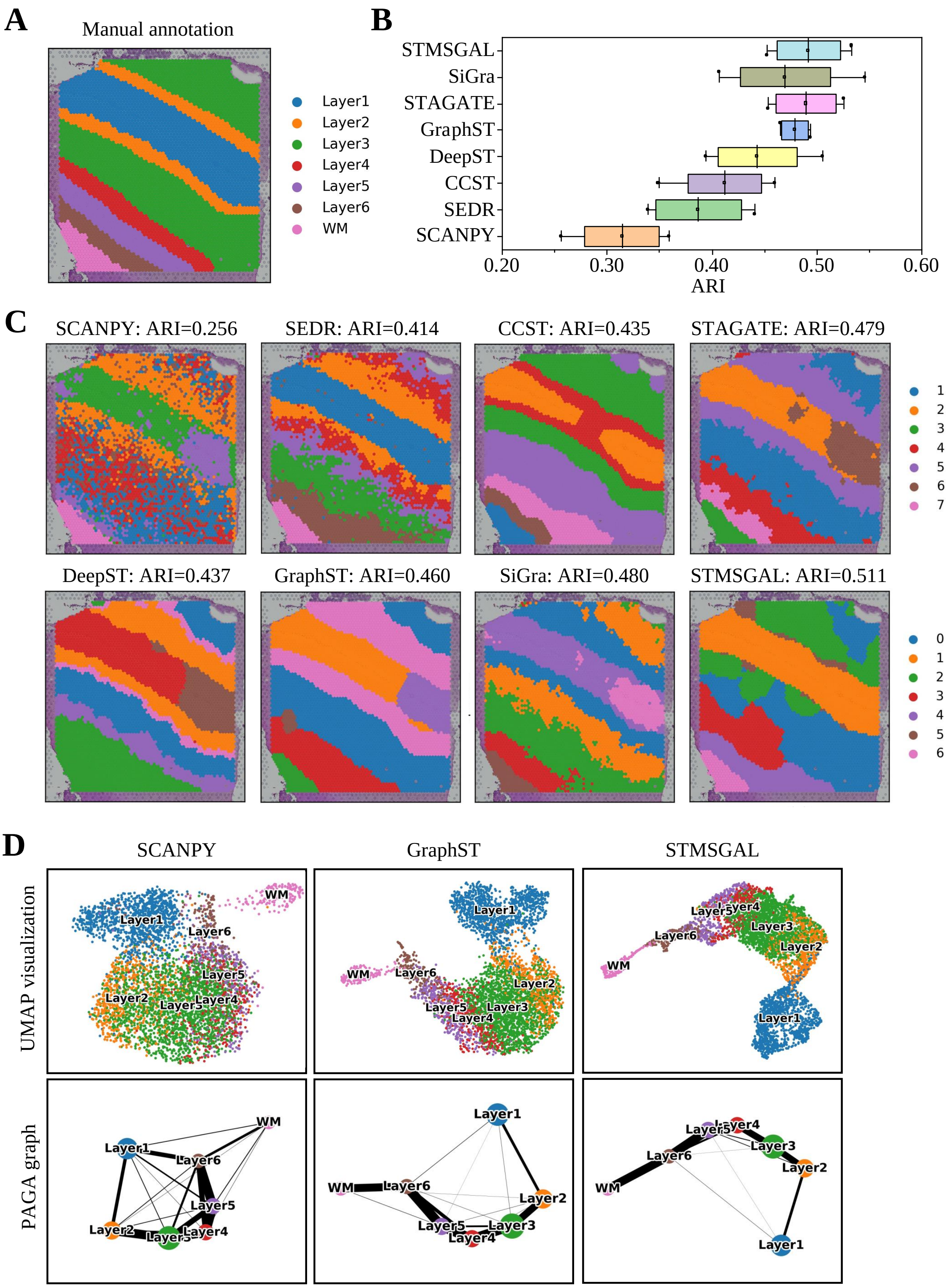

Figure 2. STMSGAL improves the identification of layer structures in the DLPFC tissue. **(A)** Ground-truth segmentation of 6 cortical layers and one white matter layer in the DLPFC section 151509. **(B)** Boxplots of ARI computed by STMSGAL and other seven methods in the DLPFC sections, from 151507 to 151510. **(C)** Cluster assignments generated by SCANPY, SEDR, CCST, STAGATE, DeepST, GraphST, SiGra and STMSGAL in the DLPFC section 151509. **(D)** UMAP visualizations and PAGA graphs generated by SCANPY, GraphST, and STMSGAL embeddings in the DLPFC section 151509.

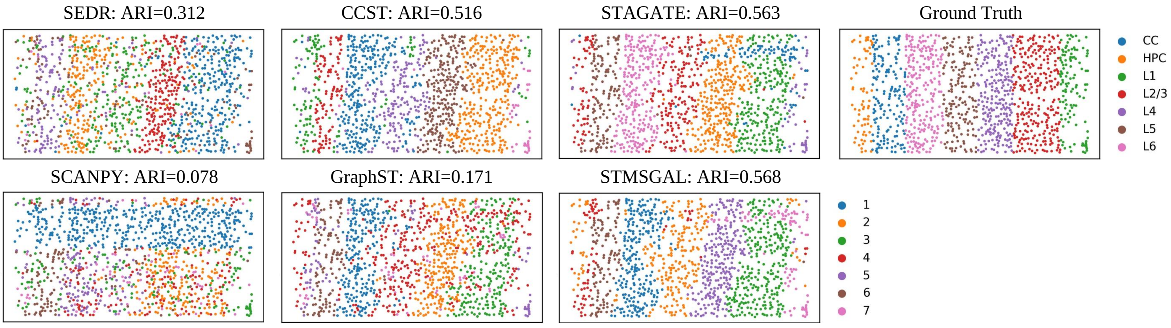

Figure 3. Spatial domains identified by SCANPY, SEDR, CCST, STAGATE, GraphST, STAGATE, and STMSGAL in the mouse visual cortex STARmap dataset.

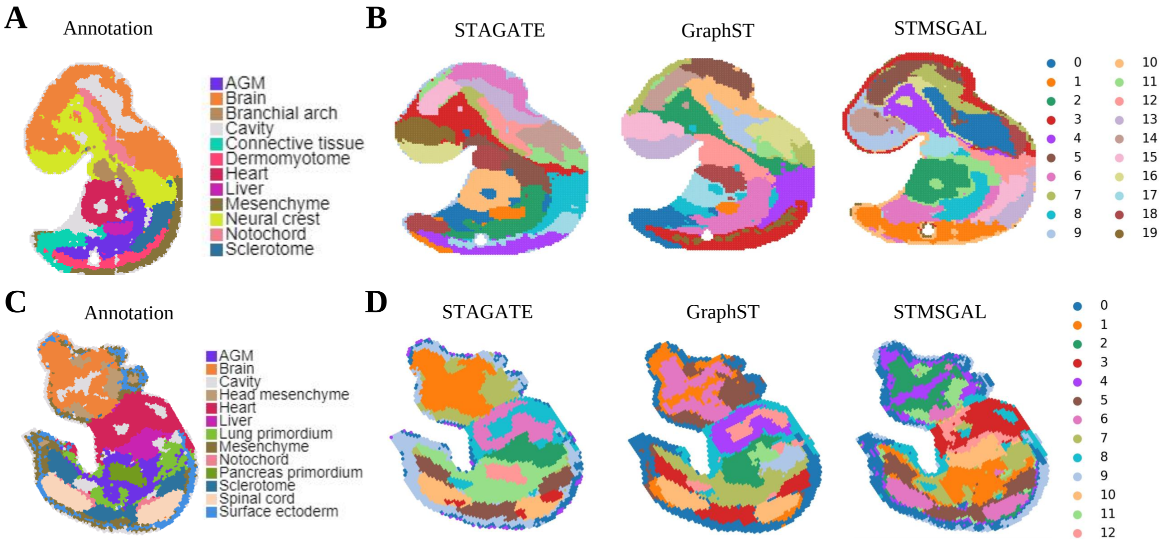

Figure 4. STMSGAL improves accurately identification of different organs in the Stereo-seq mouse embryo. **(A)** Tissue domain annotations of the E9.5_E1S1 mouse embryo data. **(B)** Cluster assignments generated by STAGATE, GraphST, and STMSGAL on E9.5_E1S1 mouse embryo data. **(C)** Tissue domain annotations of the E9.5_E2S2 mouse embryo data. **(D)** Cluster assignments generated by STAGATE, GraphST, and STMSGAL on the E9.5_E2S2 mouse embryo data.

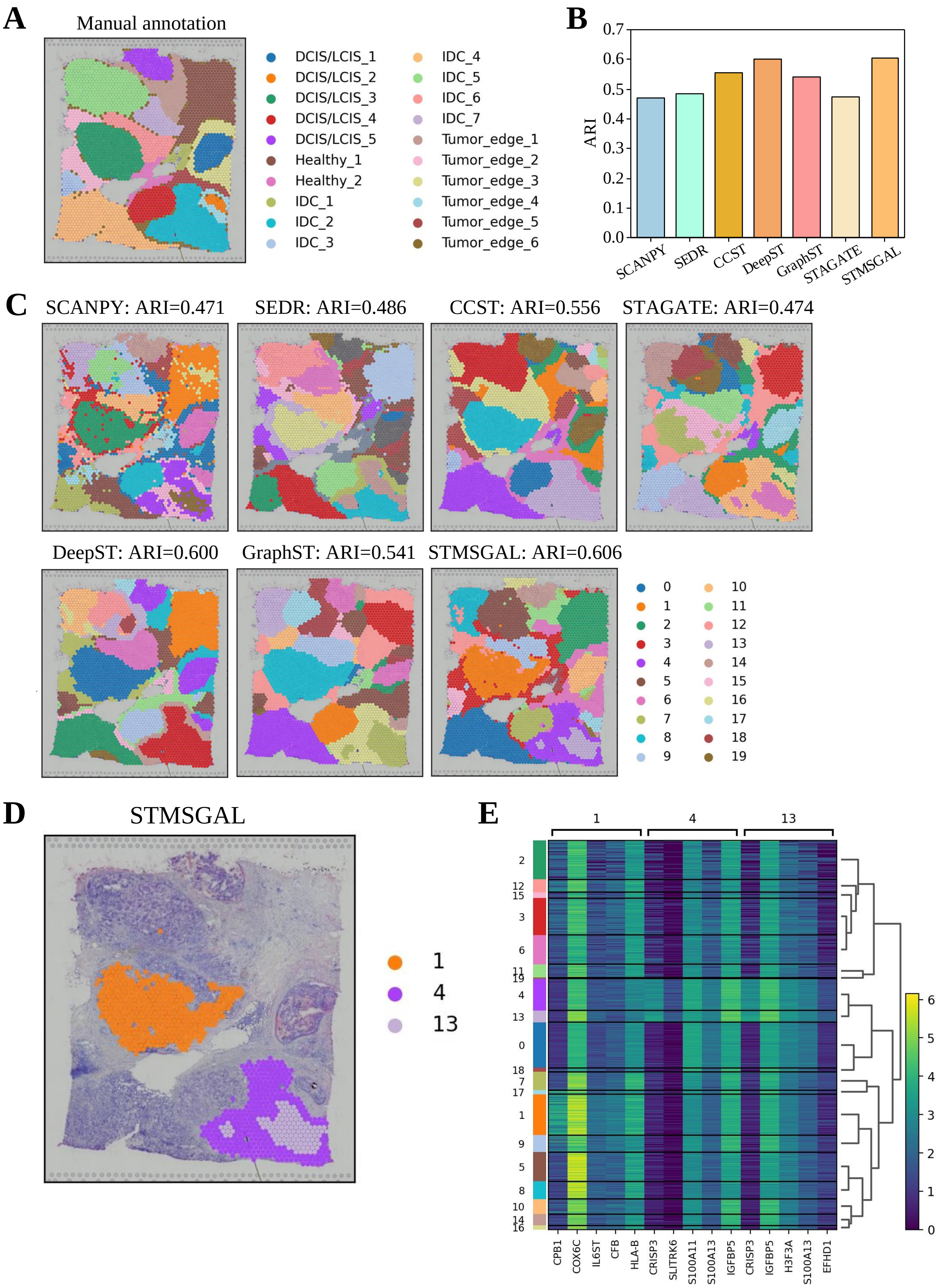

Figure 5. STMSGAL can accurately dissect spatial domains on Human Breast Cancer (Block A Section 1). **(A)** Manual pathology labeling via the H&E staining. **(B)** The average ARI values computed by SCANPY, SEDR, CCST, STAGATE, DeepST, GraphST, and STMSGAL on Human Breast Cancer (Block A Section 1). **(C)** Cluster assignments generated by SCANPY, SEDR, CCST, STAGATE, DeepST, GraphST, and STMSGAL on Human Breast Cancer (Block A Section 1). **(D)** Spatial domains identified by STMSGAL. **(E)** Heatmap of the top 5 differentially expressed genes of domains 1, 4, and 13 on Human Breast Cancer (Block A Section 1).

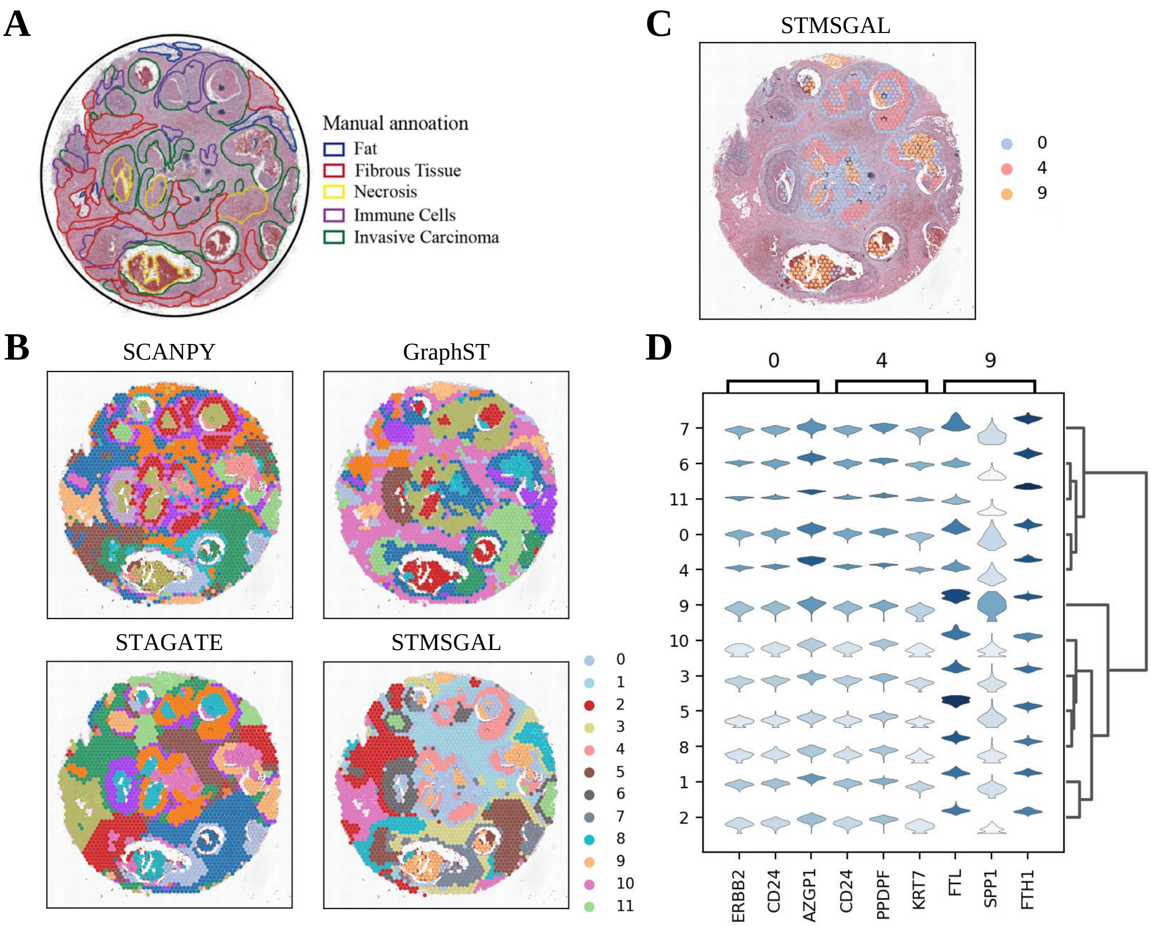

Figure 6. STMSGAL can accurately dissect spatial domains on Human Breast Cancer (DCIS). **(A)** H&E staining figures annotated by Agoko's telepathology platform on Human Breast Cancer (DCIS). **(B)** Spatial domains identified by SCANPY, GraphST, STAGATE, and STMSGAL on Human Breast Cancer (DCIS). **(C)** Spatial domains 0, 4, and 9 identified by STMSGAL. **(D)** Stacked violin plots illustrate the top 3 differentially expressed genes on spatial domains 0, 4 and 9, and their expressions on all spatial domains.

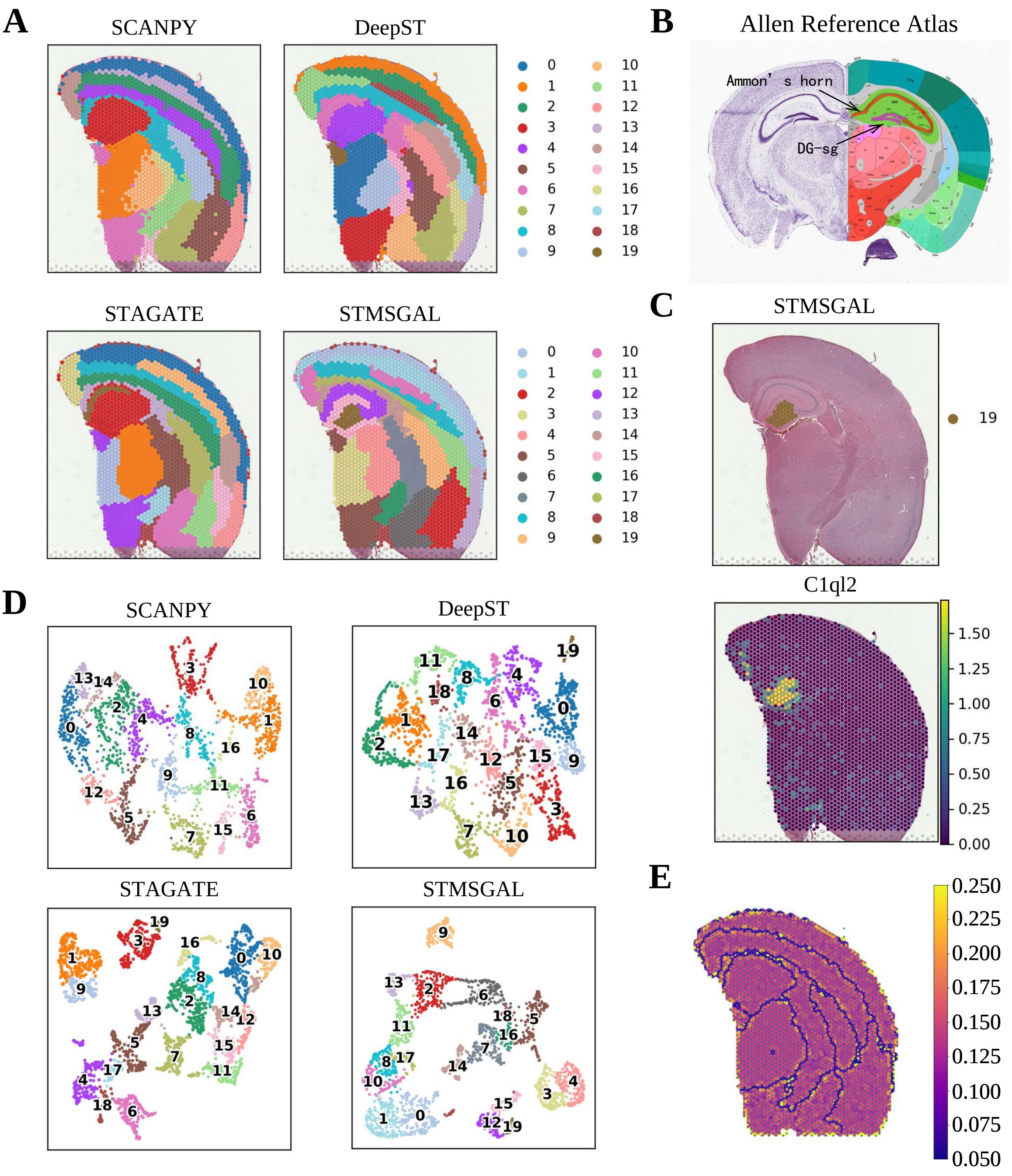

Figure 7. STMSGAL reveals spatial domains on Adult Mouse Brain (FFPE). **(A)** Spatial domains identified by SCANPY, DeepST, STAGATE, and STMSGAL. **(B)** The annotation of hippocampus structures from the Allen Reference Atlas on mouse brain. **(C)** Visualization of domains identified by STMSGAL and the corresponding marker genes. **(D)** UMAP visualization generated by SCANPY, DeepST, STAGATE, and STMSGAL embeddings, respectively. **(E)** Visualization of all attention layers of STMSGAL with the ctaSNN module. In each attention layer, nodes were arranged based on spatial contexts of spots, and edges were colored by corresponding weights.

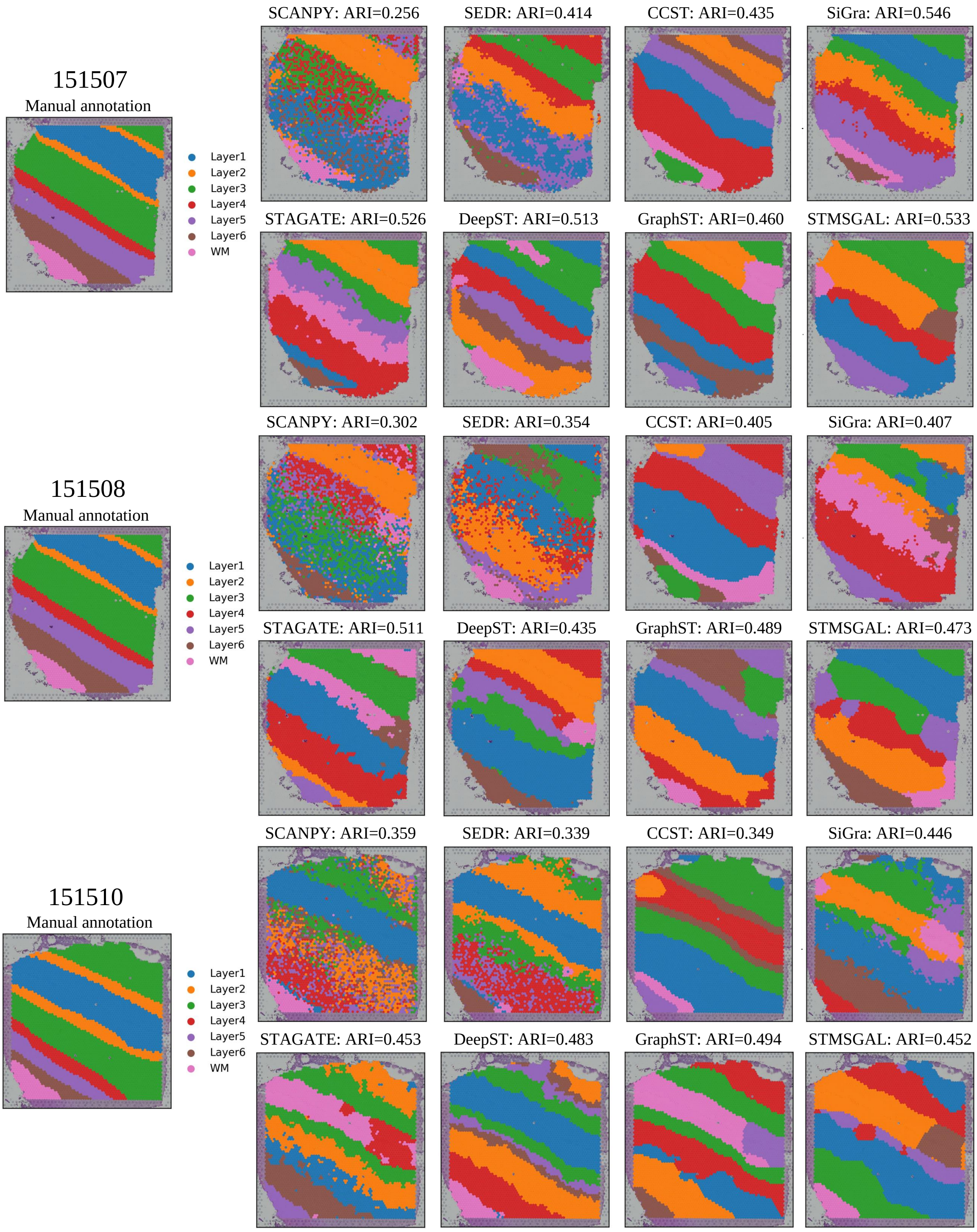

Figure S1. STMSGAL improves the identification of layer structures in the DLPFC tissue. Comparison of spatial domains identified by SCANPY, SEDR, CCST, DeepST, STAGATE, SiGra, GraphST, and STMSGAL, and manual annotations in 3 sections of human DLPFC tissues.

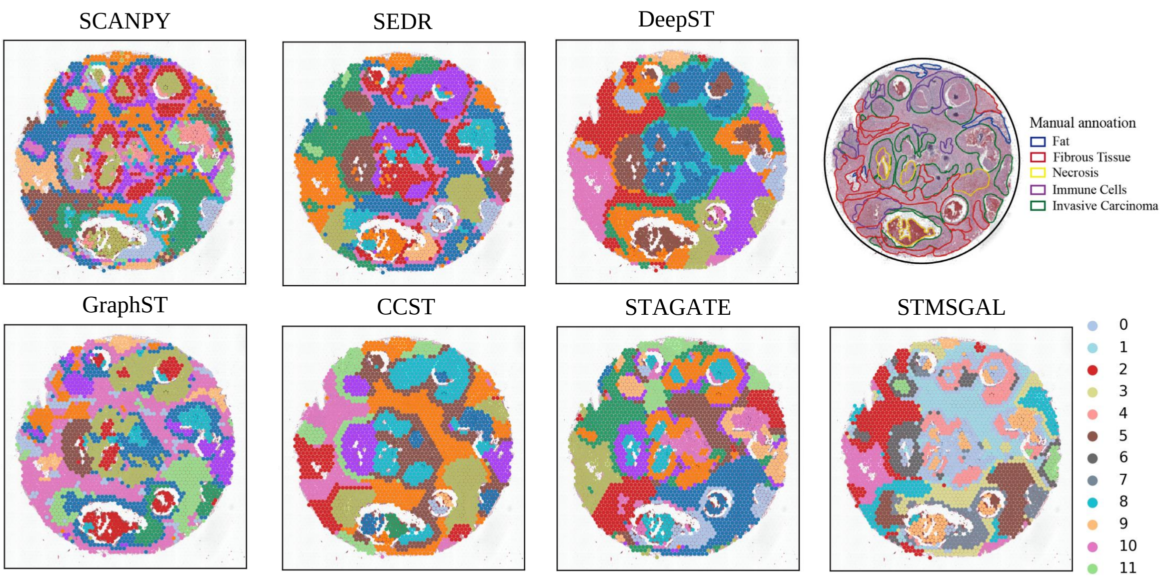

Figure S2. Cluster assignments generated by SCANPY, SEDR, CCST, STAGATE, DeepST, GraphST, and STMSGAL on Human Breast Cancer (DCIS).

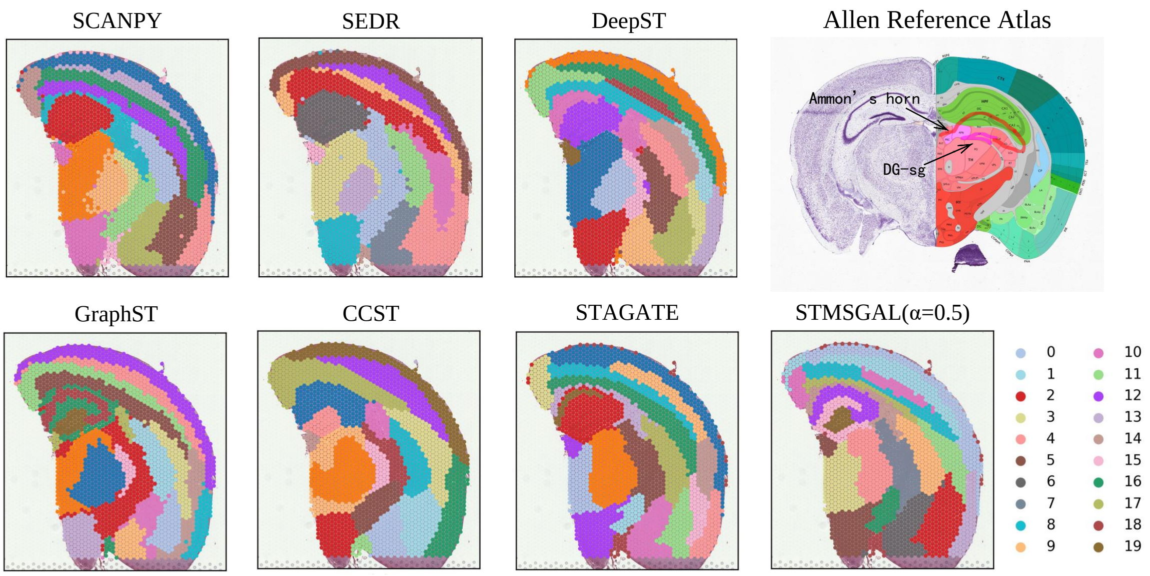

Figure S3. Cluster assignments generated by SCANPY, SEDR, CCST, STAGATE, DeepST, GraphST, and STMSGAL on Adult Mouse Brain (FFPE).

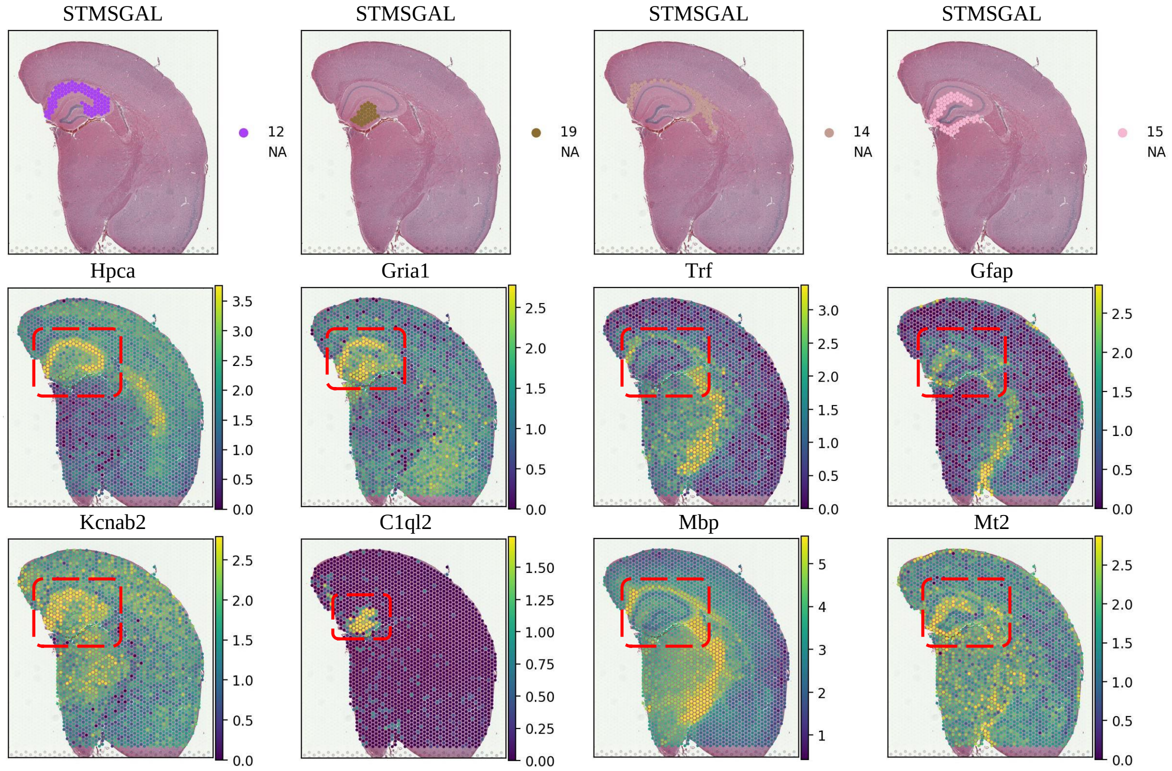

Figure S4. STMSGAL reveals spatial domains on Adult Mouse Brain (FFPE). Visualizations of spatial domains and expressions of the corresponding marker genes identified by STMSGAL with Louvain clustering on adult mouse hippocampus tissue.

Click here to access/download;Figure;STMSGAL2.pdf

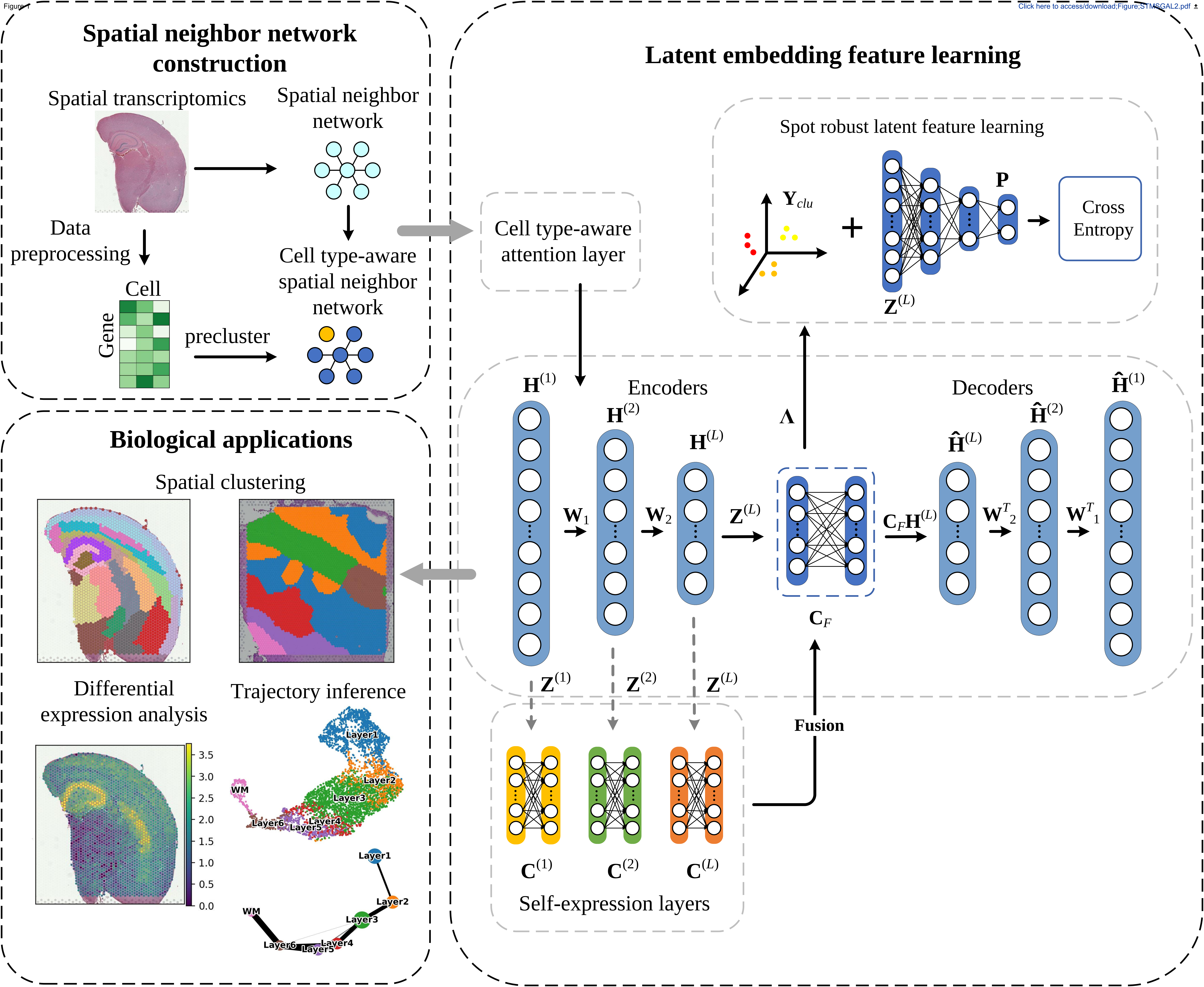

Figure 2

Click here to access/download;Figure;DLPFC.pdf

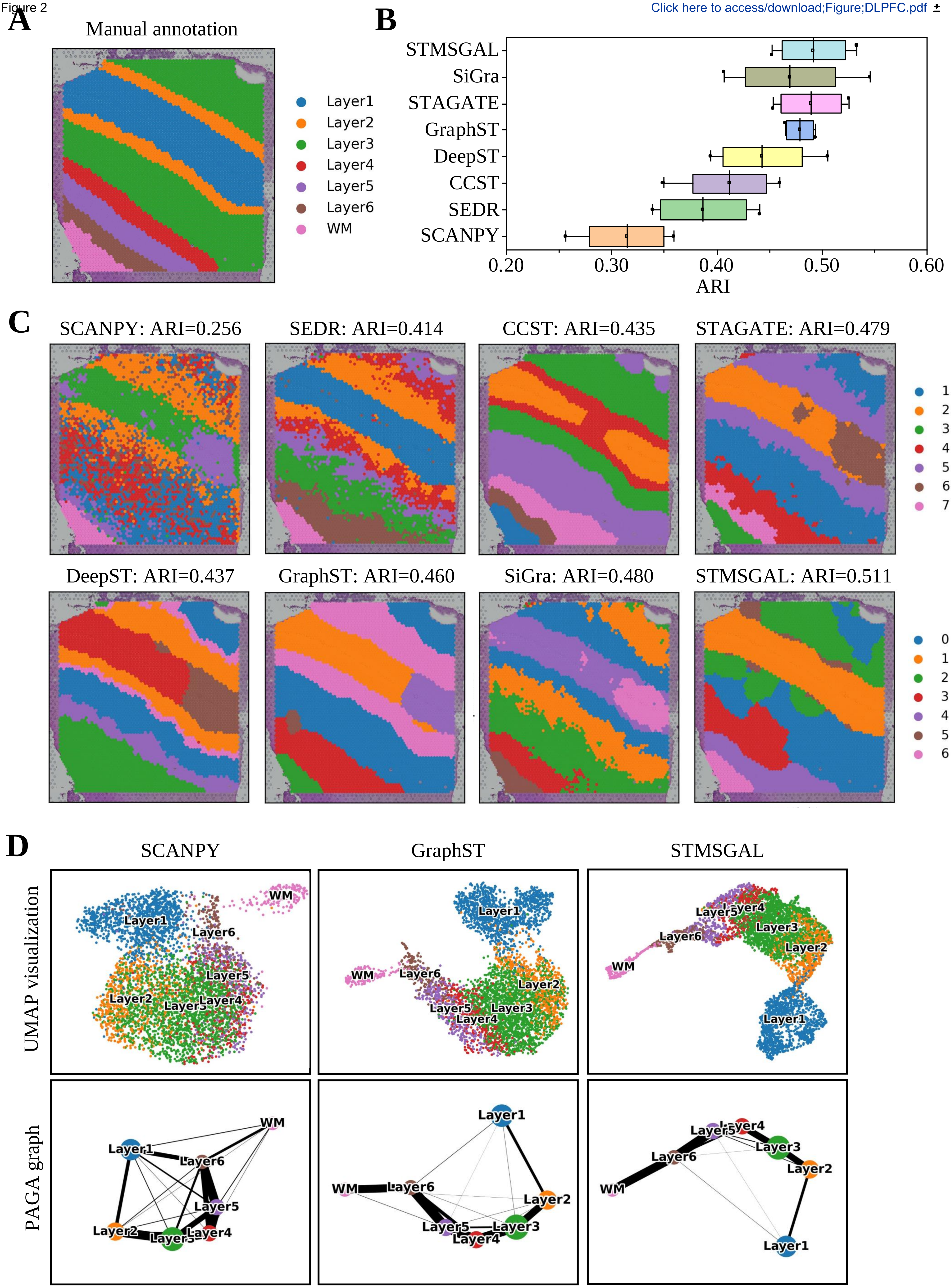

Figure 3

Click here to access/download;Figure;STARmap.pdf

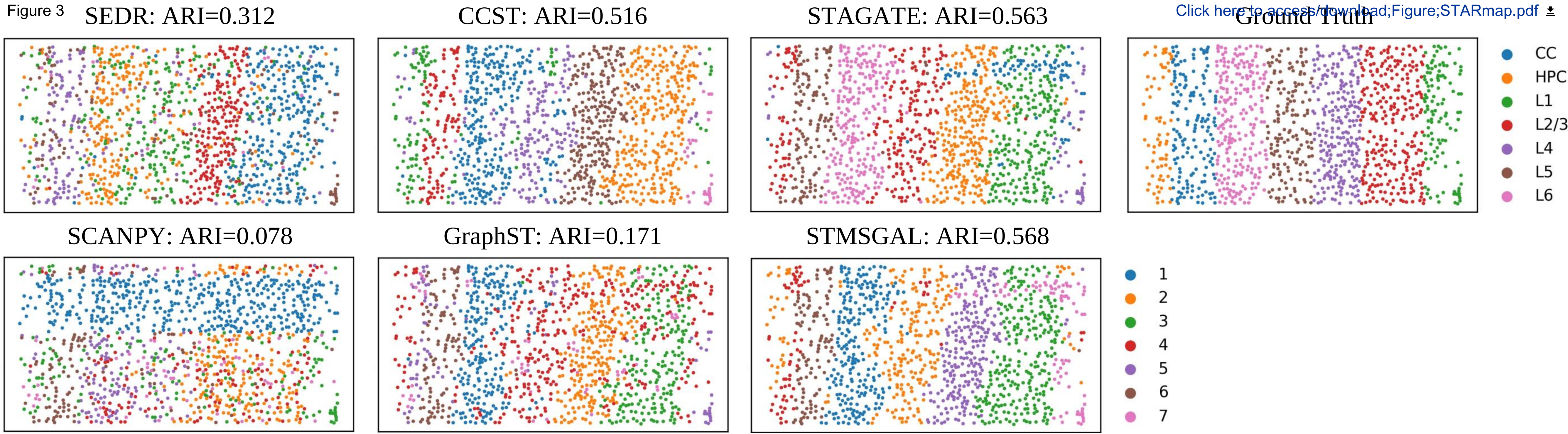

Figure 4

Click here to access/download;Figure;E9.5.pdf

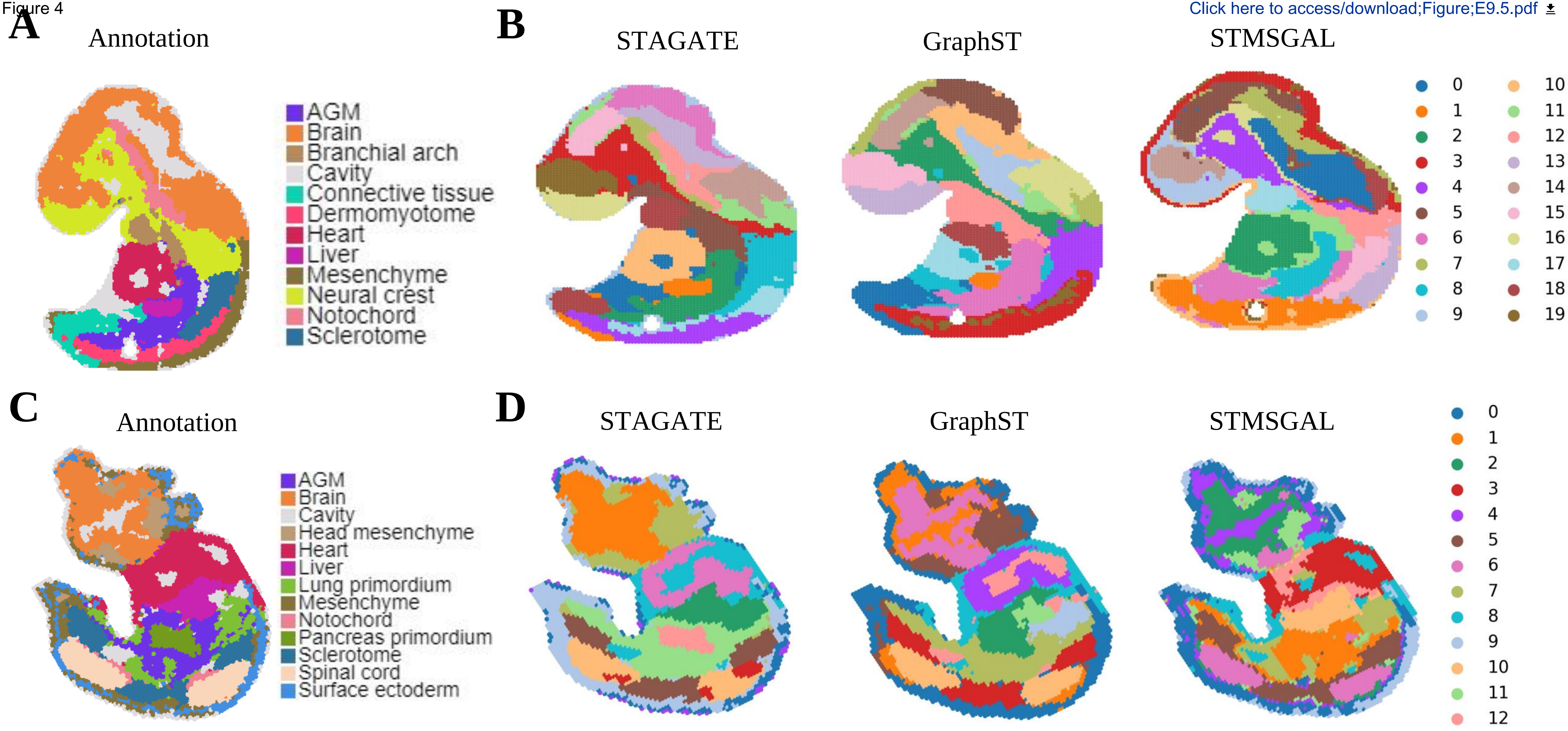

Figure 5

Click here to access/download;Figure;breast1.pdf

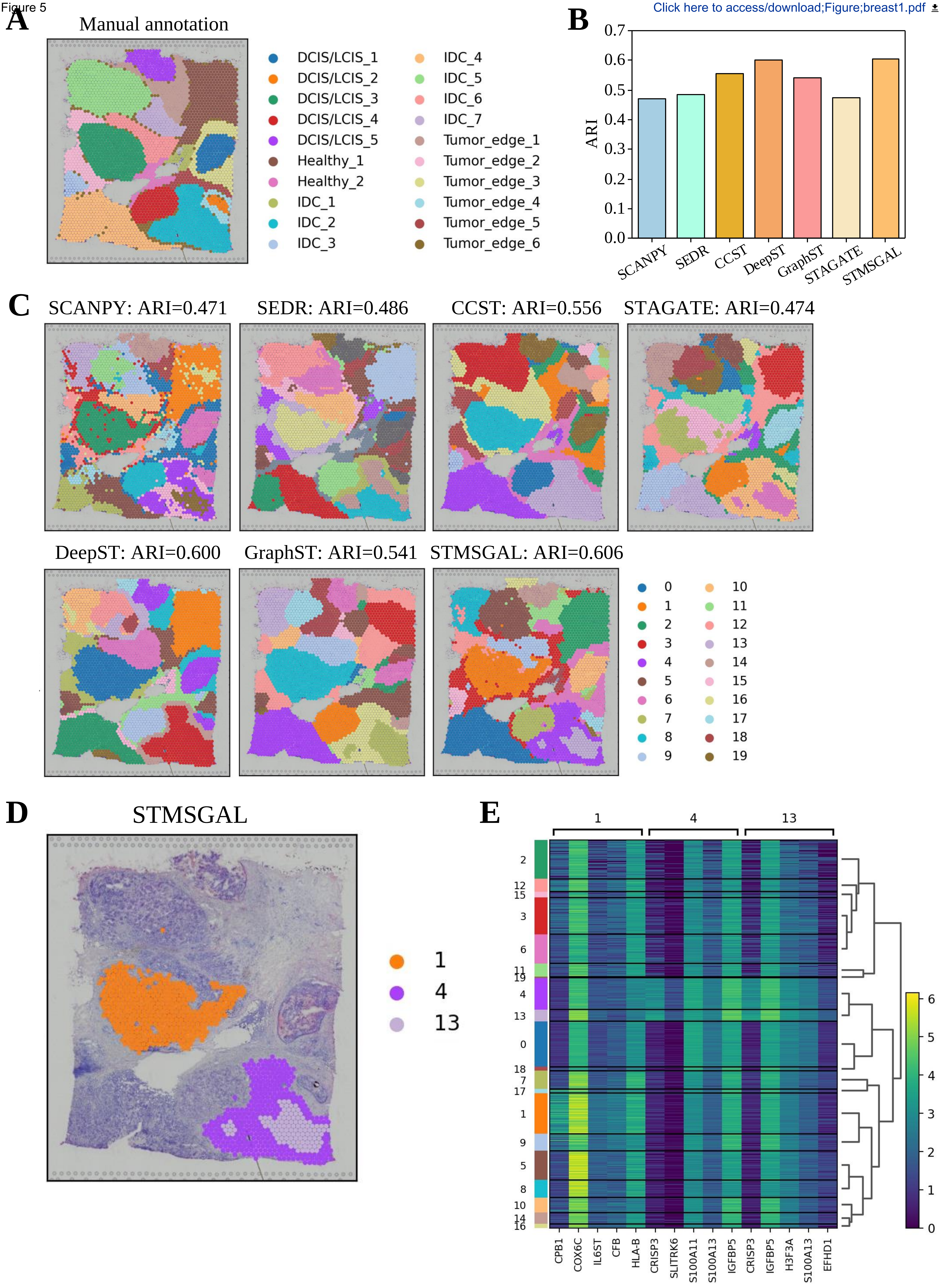

Figure 6
Click here to access/download;Figure;breast2.pdf

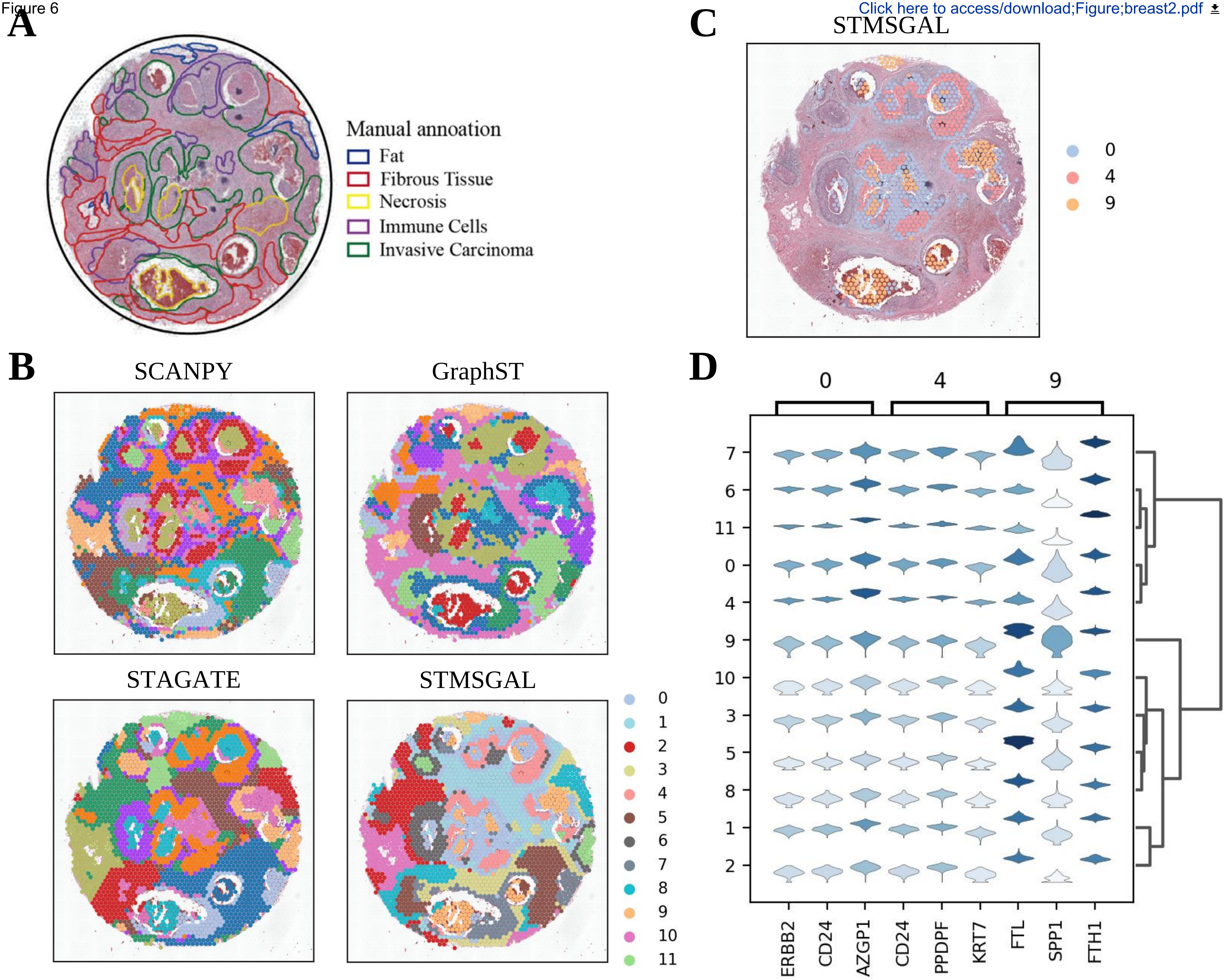

Figure 7

Click here to access/download;Figure;brain.pdf

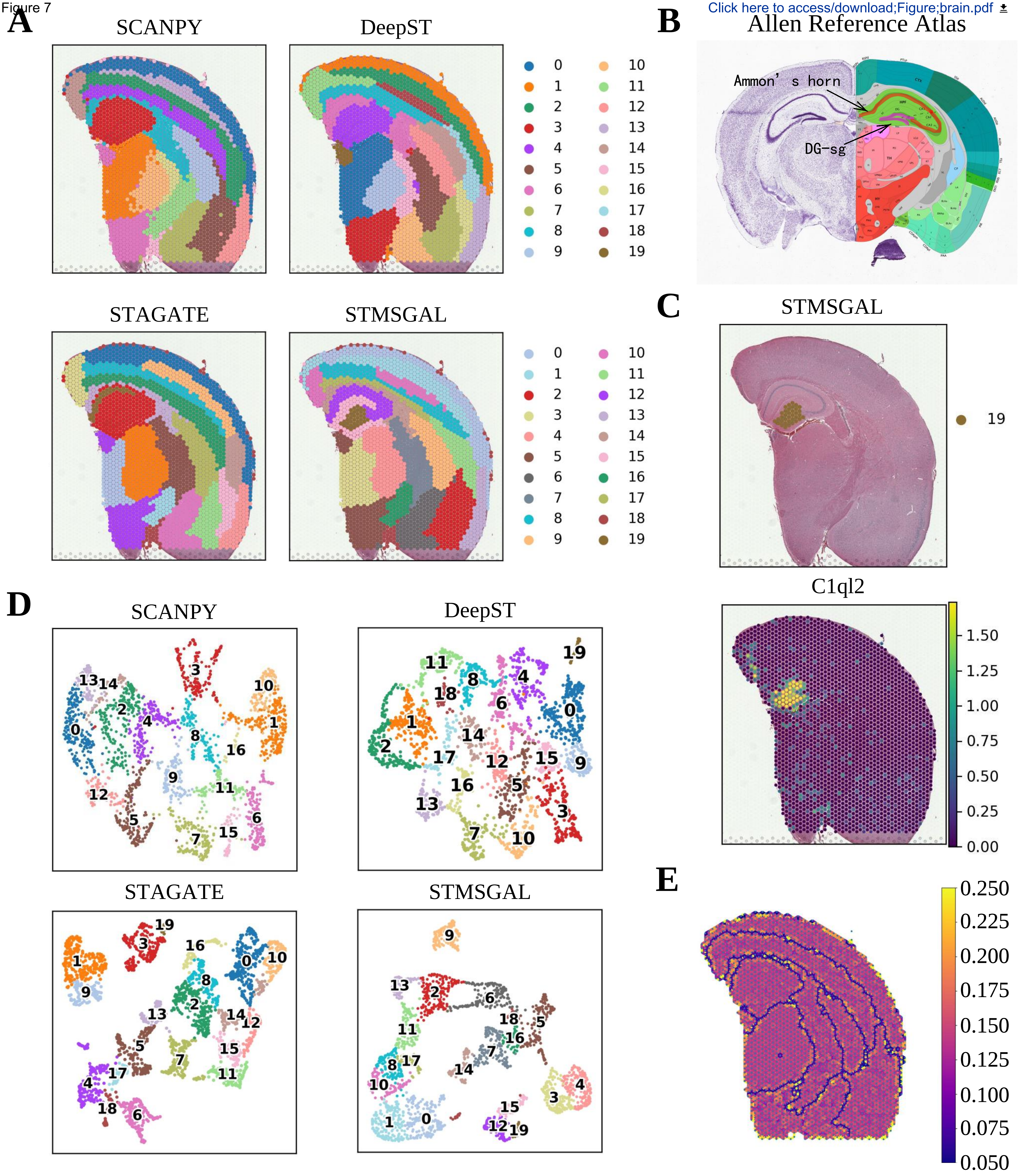

Click here to access/download;Figure;supDLPFC.pdf

Figure S1

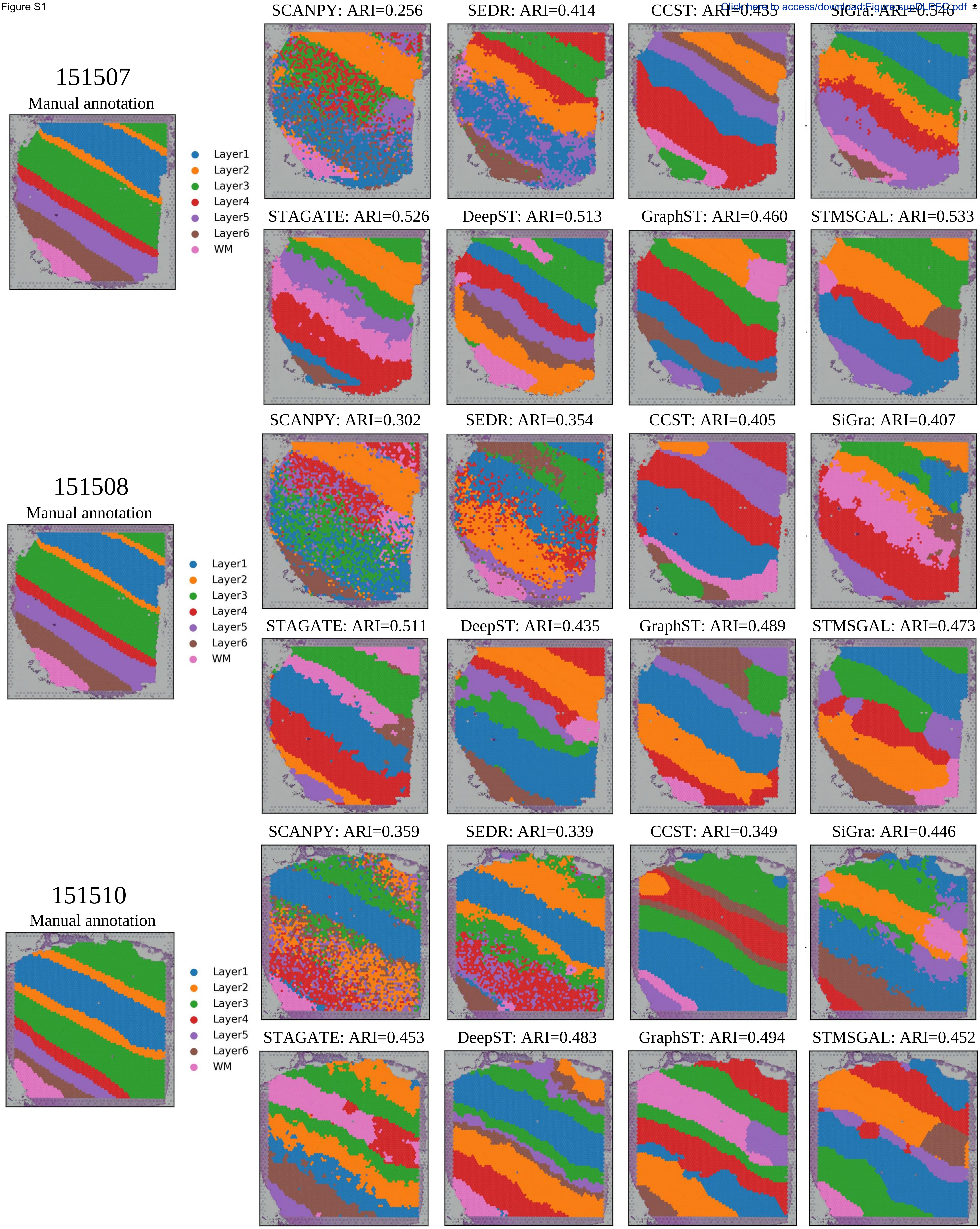

Figure S2

Click here to access/download;Figure;supbreast.pdf

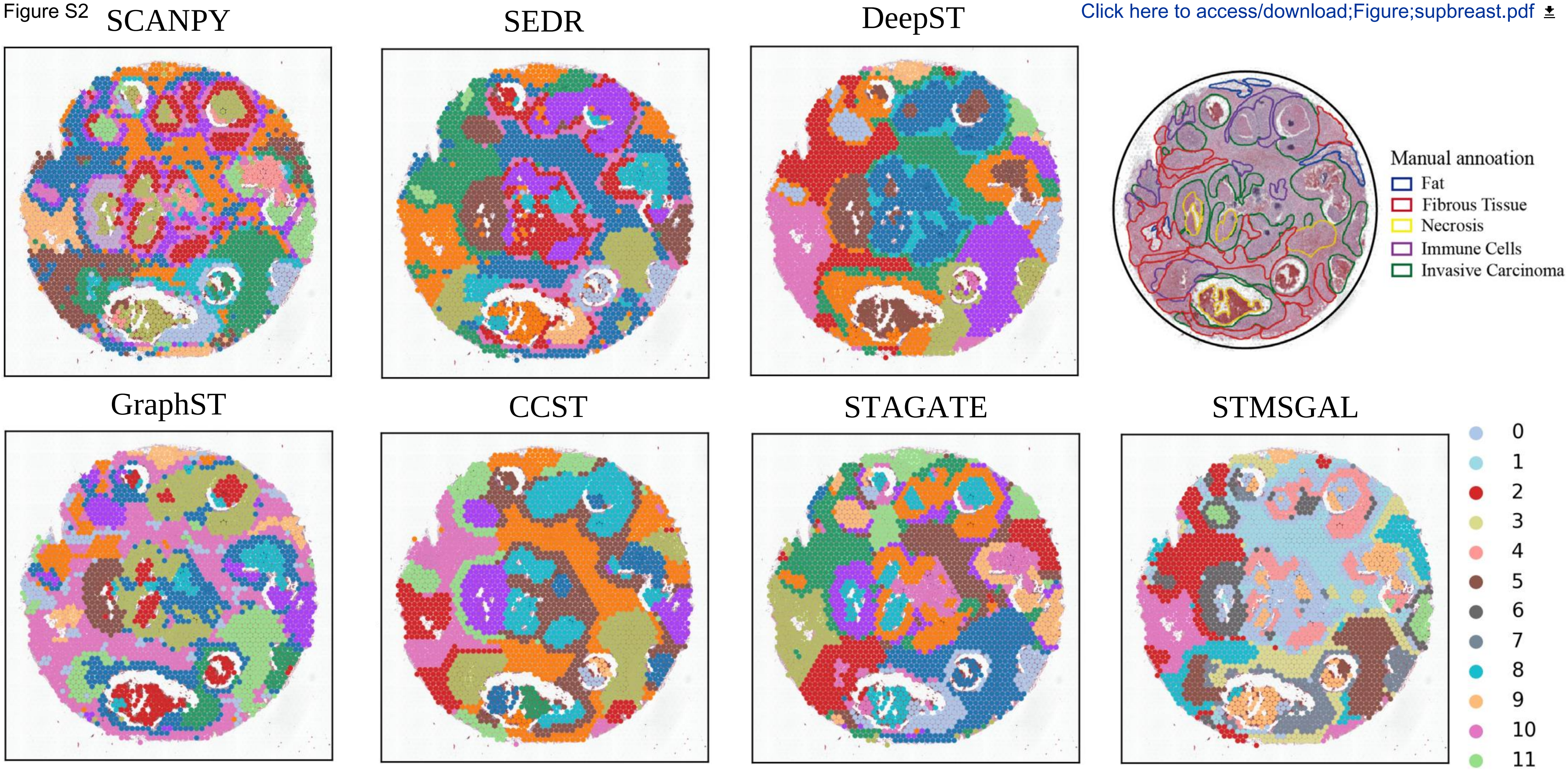

Figure S3

Click here to access/download;Figure;supbrain.pdf

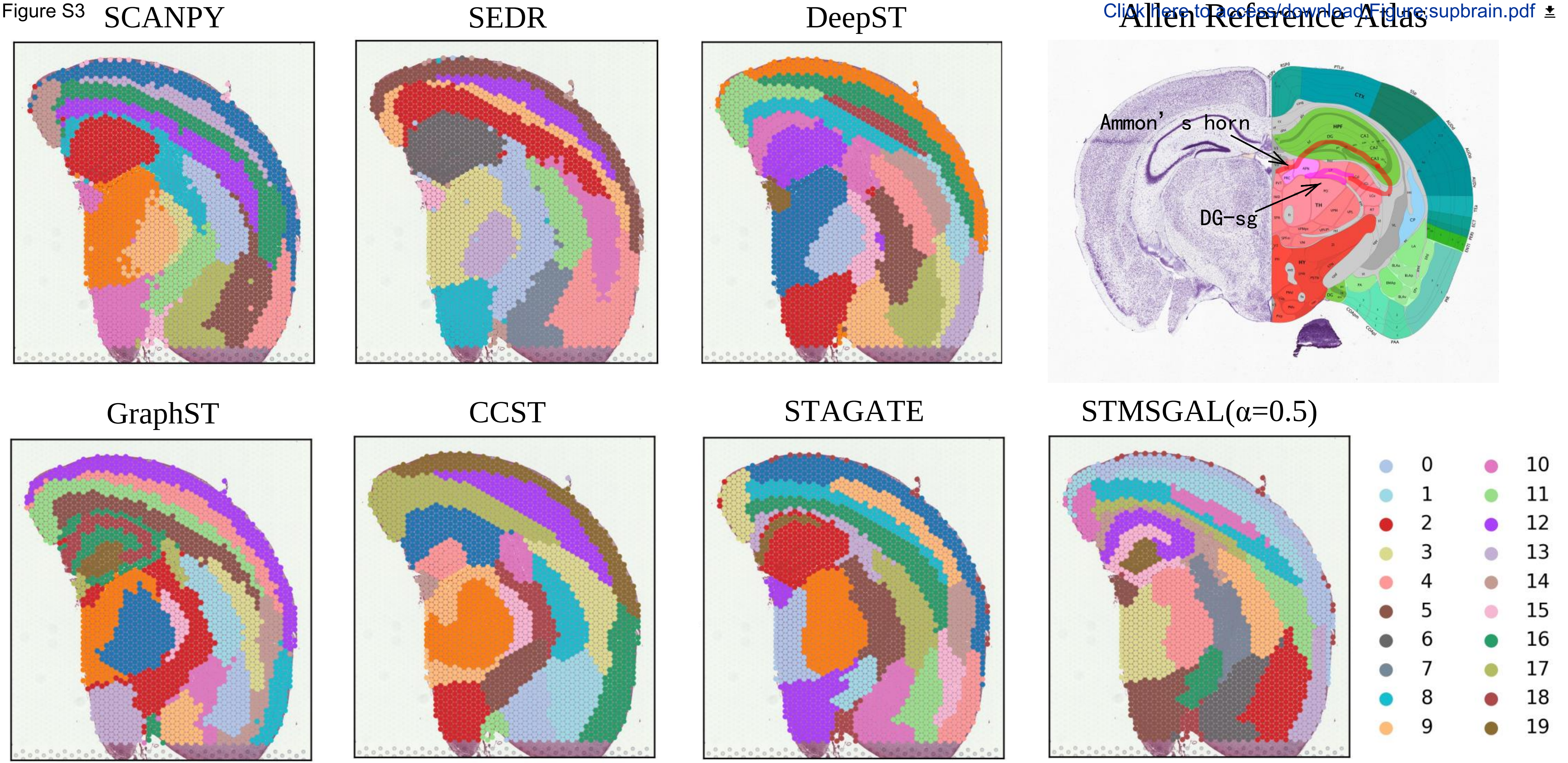

Figure S4

Click here to access/download;Figure;supbrain-expression.pdf

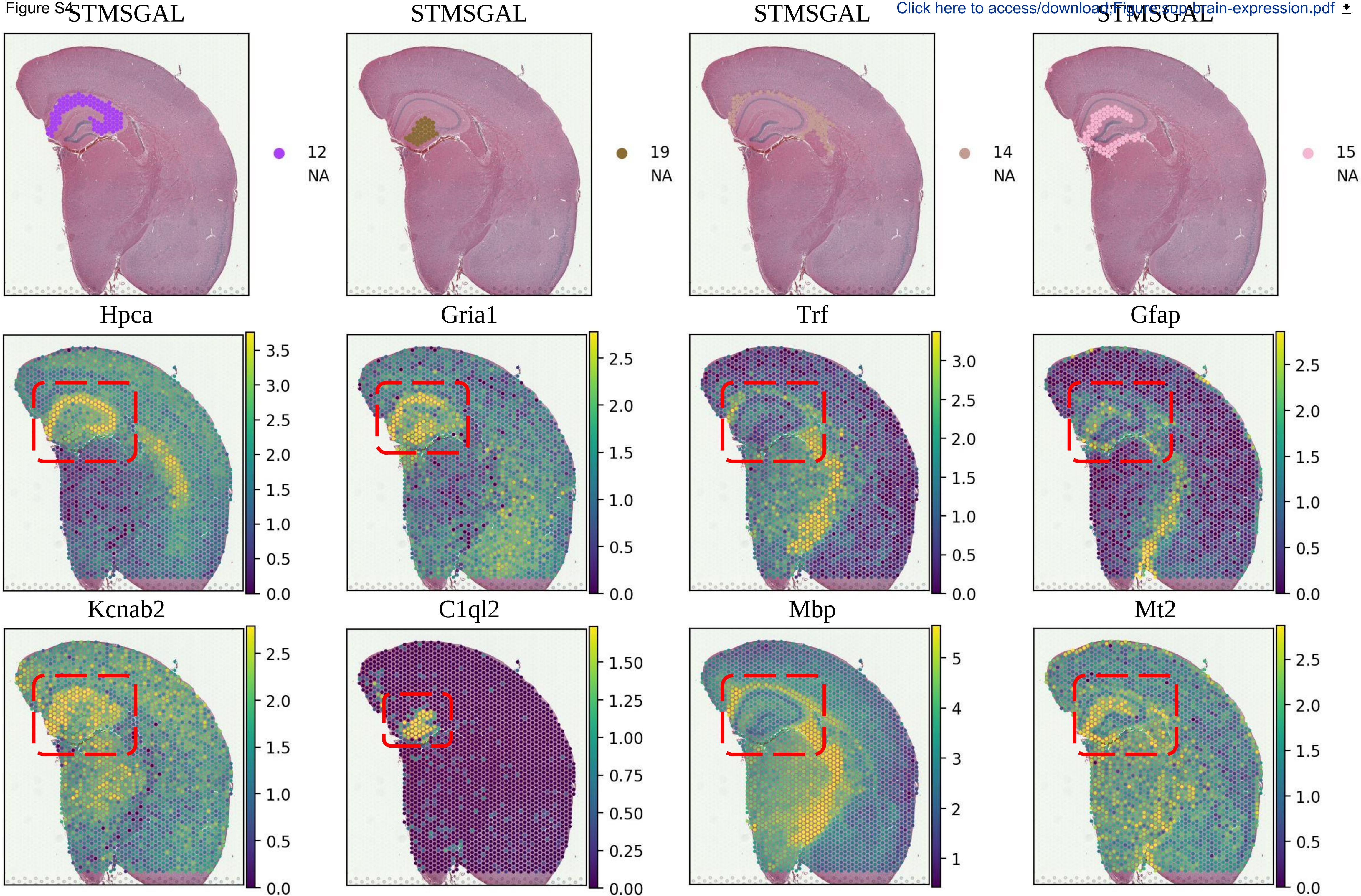

LaTex - Other (clo, cls, sty etc.)

Click here to access/download
Supplementary Material
vancouver-authoryear.bst

LaTex - Other (clo, cls, sty etc.)

Click here to access/download
Supplementary Material
oup-contemporary.cls

Bibliography files

Click here to access/download
Supplementary Material
STMSGAL2.bib

Primary manuscript file (.tex)

Click here to access/download
Supplementary Material
STMSGAL-GigaScience-11-5.tex
