# Supplementary material for: Unveiling patterns in spatial transcriptomics data: a novel approach utilizing graph attention autoencoder and multiscale deep subspace clustering network
Source: Gigascience. 2025 Jan 13;14:giae103. doi: 10.1093/gigascience/giae103 (PMC11727722; doi:10.1093/gigascience/giae103)
Supplement: giae103_Response_to_Reviewer_Comments_Original_Submission [file giae103_response_to_reviewer_comments_original_submission.pdf]

Author's Response To Reviewer Comments

Dear editor and reviewers,

We're very grateful to your patient, meticulous, and professional review work with the aim of improving the quality of our manuscript.

We need to upload our file to respond to your reviews by point-by-point because our response file contains many figures.

However, we can not upload our reponses to Reviewers in the form of file. So we had to upload our repsonses to Reviewers in "Personal cover".

Could you like to refer to "Personal cover" for our responses to Reviewers, please?

We're very thankful for your help!

With best regards!

Sincerely yours,

Lihong Peng

Hunan University of Technology
