# Supplementary material for: Unveiling patterns in spatial transcriptomics data: a novel approach utilizing graph attention autoencoder and multiscale deep subspace clustering network
Source: Gigascience. 2025 Jan 13;14:giae103. doi: 10.1093/gigascience/giae103 (PMC11727722; doi:10.1093/gigascience/giae103)
Supplement: giae103_Response_to_Reviewer_Comments_Revision_1 [file giae103_response_to_reviewer_comments_revision_1.pdf]

Author's Response To Reviewer Comments

We are very grateful to the editor and reviewers for patient, meticulous, and professional review work with the aim of improving the quality of our manuscript. We have addressed the concerns of the editor and reviewers in the revised manuscript. In particular, the following revisions have been made.

- I see you indicate three "corresponding" authors. Please note that we can allow no more than 2 authors with this role. To indicate a supervisor role, you may also use an indication of "senior author" instead.
Response: We're very thankful for your comment! We have revised them and removed "Min Chen" as the co-first author. Currently, there are only two "corresponding" authors.

- in the "code availability" section, you indicate both MIT and CCo licences - I assume the MIT licence refers to the code, and cc0 to the data in the github repo? If so, please clarify in the "availability" section what the licences refer to.
Response: Thanks a lot! We have revised it in the revised manuscript as follows: MIT license for the code, Creative Commons CC0 1.0 Public Domain Dedication for the filtered spatial transcriptomic data.

- Please move all in-text URLs/DOIs to the bibliography and cite them by reference number, rather than including the URL directly (also in the data availability section). We treat online sources as citable items. Please refer to the citation guidelines on our homepage for details regarding the reference format.
Response: Thank you very much! We have revised them in the manuscript.

- At this stage, you may remove any highlighting/tracking of changes in color that was made for the purpose of peer review.
Response: Thank you very much! We have updated the manuscript.

Reviewer #1: I have reviewed the response letter and appreciate that the authors have conducted additional experiments based on my comments. I have no further concerns. I recommend that the experiments detailed in the response letter should be included in the revised manuscript.
Response: Thank you very much! We have updated the manuscript.
