# Supplementary material for: Unveiling patterns in spatial transcriptomics data: a novel approach utilizing graph attention autoencoder and multiscale deep subspace clustering network
Source: Gigascience. 2025 Jan 13;14:giae103. doi: 10.1093/gigascience/giae103 (PMC11727722; doi:10.1093/gigascience/giae103)
Supplement: giae103_Response_to_Reviewer_Comments_Revision_2 [file giae103_response_to_reviewer_comments_revision_2.pdf]

Author's Response To Reviewer Comments

We are very grateful to the chief editor and reviewers for patient, meticulous, and professional review work with the aim of improving the quality of our manuscript. The font in the modified area is highlighted in blue. Significantly, Liqian Zhou is the first author, Xinhuai Peng and Liqian Zhou have contributed equally to this work and share first authorship.
1) Please include a citation to your new GigaDB dataset (including the DOI link) to your reference list, and cite this in the data availability section.
Response: Thank you very much! We have updated it.

2) Likewise, please also add other data sources to the bibliography as individual numbered items. Please refer to our citation guidelines on the journal homepage for details on the correct format for URL/doi citations. Please cite these datasets from the "data availability" section and also from the section "datasets" (page 3).
Response: Thank you very much! We have cite these datasets from the "data availability" section and also from the section "datasets".

3) In the data availability section, please add a line regarding DOME-ML annotations (e.g. "DOME-ML (Data, Optimization, Model and Evaluation in Machine Learning) annotations are available via a link in GigaDB[xx]."
Response: Thank a lot! We have add a line regarding DOME-ML annotations.

4) Please add ORCIDs for all authors to the title page, where available. We have the following on our files: ORCID iDs: Lihong Peng [0000-0002-2321-3901]; Liqian Zhou; Xinhuai Peng; Min Chen; Xianzhi He; Geng Tian [0000-0001-5752-4436]; Jialiang Yang [0000-0003-4689-8672].
Response: Thank you very much! We have updated it.

5) Minor point: You thank "anonymous" referees in the acknowledgements - please note that GigaScience has named, non-anonymous peer review, you may delete the word "anonymous".
Response: Thank you very much! We have deleted it.
6) You cite several preprints (e.g. nr 45, 46, 60, 70, 77, 78). Please check if there is meanwhile a published journal version for any of these preprints, and if there is, please cite the journal version instead. (If there is only a preprint version citing the preprint is totally fine though).
Response: Thank you very much! We have updated it.

7) Please upload editable source files for the text to EM - LaTex or WORD, whatever you used. (currently we only have a PDF).
Response: Thank you very much! We have uploaded them.

8) Please also ensure that your revised manuscript conforms to the journal style, which can be found in the Instructions for Authors on the journal homepage.
Response: Thank you very much! We have revised manuscript.
