# Supplementary material for: Unveiling patterns in spatial transcriptomics data: a novel approach utilizing graph attention autoencoder and multiscale deep subspace clustering network
Source: Gigascience. 2025 Jan 13;14:giae103. doi: 10.1093/gigascience/giae103 (PMC11727722; doi:10.1093/gigascience/giae103)
Supplement: giae103_Response_to_Reviewer_Comments_Revision_4 [file giae103_response_to_reviewer_comments_revision_4.pdf]

Author's Response To Reviewer Comments

We are very grateful to the chief editor and reviewers for patient, meticulous, and professional review work with the aim of improving the quality of our manuscript. Significantly, Liqian Zhou is the first author, Xinhuai Peng and Liqian Zhou have contributed equally to this work and share first authorship.
1) Please use the GigaDB DOI link in reference [107] instead of the URL: The link to use is: https://doi.org/10.5524/102582 (the URL you used is less stable long-term than a doi)
Response: Thank you very much! We have revised the link.

2) We have received the unique identifier for your DOME-ML annotations, which will be released after publication. Please revise the sentence in the data availability section as follows:
DOME-ML (Data, Optimization, Model and Evaluation in Machine Learning) annotations are available via a link in GigaDB [107] and via accession cji1mirt7b in the DOME registry [108]. Please add this reference:
[108] DOME registry. https://registry.dome-ml.org/search
Response: Thank you very much! We have revised the sentence in the data availability section.

3) At this point, you may remove and highlighting / tracking of changes in blue and submit a "clean" version for production.
Response: Thank a lot! We have removed highlighting / tracking of changes in blue.
