# Supplementary material for: Unveiling patterns in spatial transcriptomics data: a novel approach utilizing graph attention autoencoder and multiscale deep subspace clustering network
Source: Gigascience. 2025 Jan 13;14:giae103. doi: 10.1093/gigascience/giae103 (PMC11727722; doi:10.1093/gigascience/giae103)
Supplement: giae103_Reviewer_2_Report_Original_Submission — Zixuan Cang -- 4/8/2024 [file giae103_reviewer_2_report_original_submission.pdf]

Reviewer Report

Title: Unveiling Patterns in Spatial Transcriptomics Data: A Novel Approach Utilizing Graph Attention Autoencoder and Multi-Scale Deep Subspace Clustering Network

Version: Original Submission **Date:** 4/8/2024

Reviewer name: Zixuan Cang

Reviewer Comments to Author:

This paper introduces a tool, STMSGAL that generates spatial context-aware embeddings for spots in spatial transcriptomics data. The tool mainly contains three components, a cell type-aware neighborhood graph, a graph attention network, and a subspace clustering network. The tool is tested on some 10X Visium and STARmap data and compared to several existing methods.Specific points:1. Many parts of the proposed method, except the multi-scale deep subspace clustering part, seem to largely resemble STAGATE, which uses a cell type-aware spatial graph and a graph attention autoencoder. While STAGATE is briefly mentioned in Introduction, the differences and relations should be clarified in detail in the Methods section as well.2. The neural network part looks very similar to the method described in reference [51]. It is mentioned in "Latent embedding feature learning" section, that this approach is inspired by [51]. It should be clarified if the approach utilizes the method described in [51] or is it a novel extension inspired by [51].3. Due to the previous concerns, the contribution of STMSGAL is rather unclear given the current benchmark and comparison results. On the benchmark side, there are many different technologies but only two (Visium and STARmap) are used here. Regarding comparison, STAGATE should at least be added to the compared methods due to the similarity in approaches. A recent work (Yuan, Zhiyuan, et al. "Benchmarking spatial clustering methods with spatially resolved transcriptomics data." Nature Methods (2024): 1-11.) systematically benchmarked a collection of clustering methods for spatial transcriptomics data. I suggest the authors to use some of the datasets in this benchmark paper to extend the current benchmark to more technologies, and add comparisons to the top performers according to the benchmark paper.4. Why the clustering results from the multi-scale deep subspace clustering is not directly used for identifying spatial domains instead of performing clustering again using Leiden/Louvain/mclust? Also, could the authors explain the rationale of using different clustering methods for different datasets?5. It would be helpful to perform some ablation analysis to clarify what are the major contributors to the better performance compared to other methods. I suggest adding results 1) without the additional optimization step on DLPFC dataset and 2) without the clustering module in the graph neural network.

Level of Interest

Please indicate how interesting you found the manuscript: Choose an item.

Quality of Written English

Please indicate the quality of language in the manuscript: Choose an item.

Declaration of Competing Interests

Please complete a declaration of competing interests, considering the following questions:

- Have you in the past five years received reimbursements, fees, funding, or salary from an organisation that may in any way gain or lose financially from the publication of this manuscript, either now or in the future?
- Do you hold any stocks or shares in an organisation that may in any way gain or lose financially from the publication of this manuscript, either now or in the future?
- Do you hold or are you currently applying for any patents relating to the content of the manuscript?
- Have you received reimbursements, fees, funding, or salary from an organization that holds or has applied for patents relating to the content of the manuscript?
- Do you have any other financial competing interests?
- Do you have any non-financial competing interests in relation to this paper?

If you can answer no to all of the above, write 'I declare that I have no competing interests' below. If your reply is yes to any, please give details below.

I declare that I have no competing interests

I agree to the open peer review policy of the journal. I understand that my name will be included on my report to the authors and, if the manuscript is accepted for publication, my named report including any attachments I upload will be posted on the website along with the authors' responses. I agree for my report to be made available under an Open Access Creative Commons CC-BY license (http://creativecommons.org/licenses/by/4.0/). I understand that any comments which I do not wish to be included in my named report can be included as confidential comments to the editors, which will not be published.

Choose an item.

To further support our reviewers, we have joined with Publons, where you can gain additional credit to further highlight your hard work (see: https://publons.com/journal/530/gigascience). On publication of this paper, your review will be automatically added to Publons, you can then choose whether or not to claim your Publons credit. I understand this statement.

Yes Choose an item.
